# Microbial life in deep-seated selenide veins reflected by extreme δ^34^S fractionation of framboidal pyrite

**DOI:** 10.1038/s41598-026-59857-1

**Published:** 2026-06-26

**Authors:** Stephanie Lohmeier, Alexandre Raphael Cabral, Michael Wiedenbeck, Armin Zeh, Graciela M. Sosa, Alfons M. van den Kerkhof, Bodo-Carlo Ehling

**Affiliations:** 1 Institute of Geotechnology and Mineral Resources, Clausthal University of Technology, Adolph-Roemer Straße 2A, 38678 Clausthal-Zellerfeld, Germany; 2 Instituto de Geociências, Universidade Federal de Uberlândia, Campus Monte Carmelo, Rodovia LMG-746, Monte Carmelo, 38500-000 MG Brazil; 3 GFZ – Helmholtz-Zentrum für Geoforschung, Telegrafenberg, 14473 Potsdam, Germany; 4 Karlsruhe Institute of Technology, Institute of Applied Geoscience, Mineralogy and Petrology, Adenauerring 20b, 76131 Karlsruhe, Germany; 5 Department of Structural Geology and Geothermics, Georg-August University Göttingen, Geoscience Center, Goldschmidtstr. 3, 37077 Göttingen, Germany; 6 Landesamt für Geologie und Bergwesen Sachsen-Anhalt, Abteilung Geologischer Dienst, An der Fliederwegkaserne 13, 06130 Halle (Saale), Germany

**Keywords:** Microbial life, Selenide mineralization, Tilkerode (Harz Mountains, Germany), δ^34^S fractionation, Framboidal pyrite, Microbial sulfate reduction, Biogeochemistry, Environmental sciences, Solid Earth sciences

## Abstract

Microbial life can be found in the continental subsurface down to depths of several kilometers, where it may affect mineral-forming and reaction processes. Sulfur isotopes provide an important tool for discriminating between deep microbial activity and abiotic processes. Here, we report the sulfur isotopic composition (δ^34^S) of pyrite in selenide-rich domains of hematite-carbonate ± selenide veins hosted in black shales from Tilkerode, Harz Mountains (Germany). Framboidal pyrite (PyII) occurs in voids in clausthalite (PbSe) formed by hematite and carbonate dissolution, but is also enclosed in anhedral pyrite (PyIII). Results of secondary-ion mass spectrometry yielded extreme δ^34^S values ranging from ‒8.5 to + 92.5‰ for PyII, and from ‒40.1 to + 79.1‰ for PyIII. Extreme positive values suggest microbial sulfate reduction (MSR) under closed-system conditions with limited sulfate availability and slow metabolism, and negative values a switch to open-system conditions. All MSR processes most likely occurred after fast uplift of the vein system from about 5 km to 1–2 km depth during Cretaceous-Tertiary reverse faulting, accompanied by cooling from 220 °C to 30–60 °C, as indicated by fluid-inclusion microthermometry and U‒Pb carbonate dating. The results demonstrate that microbial activity at great depth of 0.8 to 1.8 km is possible even in normally toxic Se-Pb-Ag-Hg-rich environments.

**Supplementary Information:**

The online version contains supplementary material available at 10.1038/s41598-026-59857-1.

## Introduction

Microbial life is known to thrive in the deep biosphere at depths of several kilometers (km) from the continental subsurface^1,2^. It is commonly limited to a depth less than about 5 km^3^, below which the maximum temperature for cell proliferation 122 °C^4^ is exceeded. Many hydrothermal ore vein systems worldwide are commonly formed at or below this temperature limit^5^, meaning that microbial life and related metabolism can potentially be involved in mineral-forming and replacement processes^6–8^. Such processes are commonly reflected by typical microfabrics in sulfide minerals, such as framboidal pyrite^6^. Additionally, bio-remediation processes are recorded by the presence of significant variations in sulfur isotopic ratios^7,8^.

Microbial sulfate reduction (MSR) by sulfate-reducing bacteria and abiotic thermochemical sulfate reduction (TSR) are almost mutually exclusive as MSR is mainly restricted to low-temperature environments (≤ 85 °C; up to 110 °C^9^, whereas TSR occurs at > 120 °C^10^ with maximum temperatures of 180–200 °C^11–15^. Such elevated temperatures are typical of deep burial diagenetic settings at depths of ≤ 6 km, which contrasts with formation depths of ≤ 2.5–3 km for low-temperature settings^13,16^. There is no defined lower limit for the onset of TSR, but reaction kinetics demand elevated temperatures^17,18^ and the availability of major reactants. Therefore, TSR is mostly inferred in Mississippi Valley-type (MVT) and sedimentary exhalative type (SEDEX) deposits, but also in (sour) hydrocarbon reservoirs with long formation times^13–15,19–26^. The main organic reactants for MSR are organic acids and other products of biodegradation, e.g., methane, while sulfur (S) as dissolved sulfate can be derived from any gypsum/anhydrite source at or near the redox-reaction site^13^. In particular iron-bearing sulfides, and to a lesser extent other sulfides, can form as by-products of hydrogen sulfide generation, provided that metals are present or transported to the reaction site^13,14,16^. Discrimination between MSR and TSR is commonly ambiguous. However, petrographic observations ‒ i.e., framboidal and finely disseminated pyrite, in or close to kerogen-rich beds, commonly points to MSR, and prismatic pyrite, close to oil/gas deposits, to TSR^14^‒ and in particular S isotopic compositions (δ^34^S)^27^ as well as carbon and oxygen isotope ratios of (by-)products can provide clear criteria, permitting discrimination of microbial from abiotic thermochemical origin^14^. Results of experiments and field observations suggest that δ^34^S values depleted between 15 and 65‰ relative to the source sulfate result from MSR in open systems with unlimited sulfate supply^7,16,28–30^, whereas thermochemical limitations permit only a smaller kinetic depletion of 15‒20‰ at 100 °C^14,16,17^. In contrast, positive δ^34^S values are more characteristic for partially to completely closed systems, in which Rayleigh fractionation can occur^8,30,31^‒ i.e., due to the loss of the lighter isotopes from the sulfate reservoir, MSR-produced sulfide shows steady increases in δ^34^S, becoming even heavier as the parental sulfate^7,32^.

Recently, framboidal pyrite aggregates were discovered in selenide-rich domains of the hematite-carbonate vein system at Tilkerode in the Harz Mountains, Germany. This system is hosted by graptolite black shales and keratophyre, which underwent exhumation as part of the Harz Mountains in the Late Cretaceous and Tertiary, when more than 2 km of Paleozoic and Mesozoic cover was removed^33–36^. The pyrite framboids are a minor component in domains of abundant selenide minerals, which contain high concentrations of toxic metals, such as silver (Ag), mercury (Hg), and lead (Pb). Considering this metal association, it would seem questionable whether microbial life could exist in such a toxic ecosystem, despite the presence of framboidal microfabrics that are indicative of a deep biosphere origin^37–41^. Here, we report on Tilkerode’s pyrite framboids and their S isotopic compositions (δ^34^S), and present results of fluid-inclusion microthermometric measurements placing constraints on temperature and pressure conditions before the time of framboid formation.

## Tilkerode’s selenide-bearing vein system

**Fig. 1:**
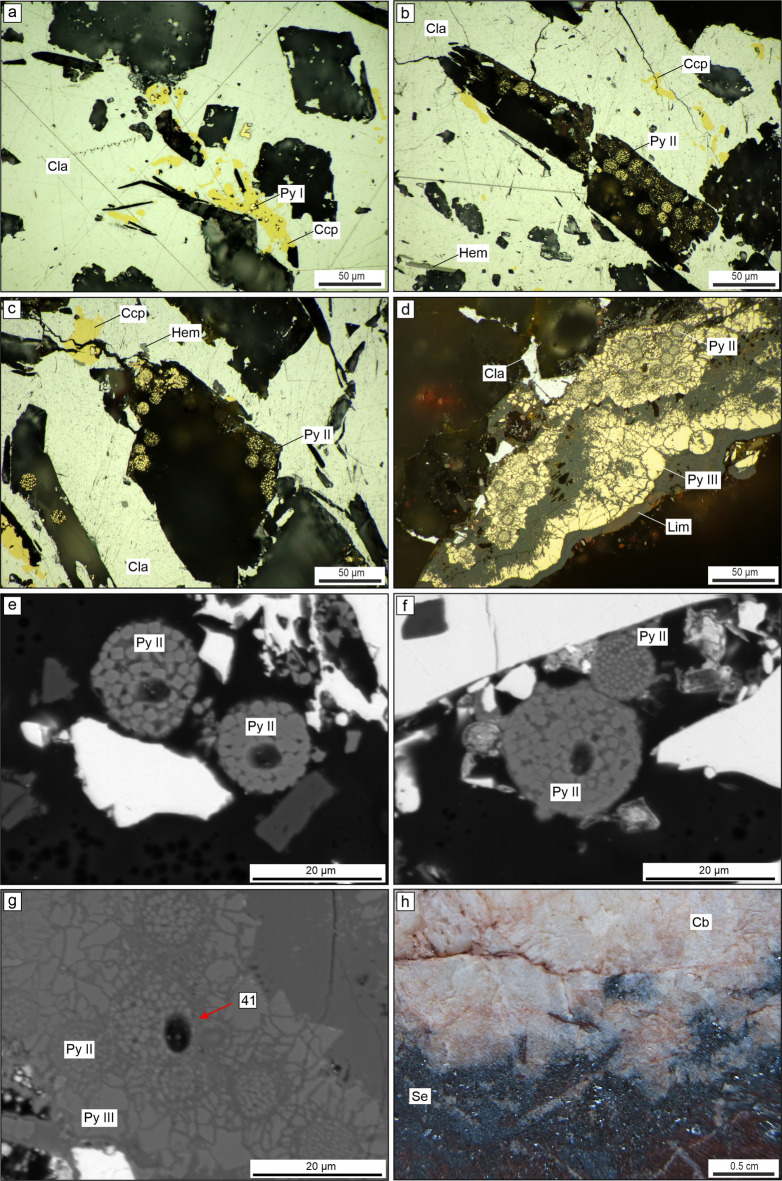
Pyrite in Tilkerode’s carbonate-hematite ± selenide vein. **a**: Subhedral to euhedral PyI crystals, spatially associated with chalcopyrite in clausthalite. **b**: PyII aggregates of framboids, < 1 μm size in diameter. The framboids display pyritohedron and octahedron morphologies. **c**: Clusters of aggregates of framboidal PyII in carbonate-dissolution voids. **d**: Framboidal PyII aggregates of different sizes overgrown by massive, subhedral to anhedral PyIII. The gray material with brownish internal reflections is limonite, formed during late-stage weathering. **e**, **f**: Clusters of aggregates of framboidal PyII in hematite-dissolution voids. Round holes in PyII aggregates are SIMS spots. **g**: Framboidal PyII surrounded by subhedral PyIII. **h**: Carbonate-hematite ± selenide vein-type mineralization. The whitish mineral is carbonate; the reddish color indicates hematite; selenides appear dark gray. **a‒d**: Photomicrographs, reflected light, oil immersion. **e**‒**g**: BSE images showing SIMS spot positions. **h**: Photograph. Abbreviations: Cb – carbonate; Cla – clausthalite; Ccp – chalcopyrite; Hem – hematite; Lim – limonite; Py – pyrite; Se – selenides.

The historical mining district of Tilkerode hosts a low-temperature, hydrothermal, carbonate-hematite ± selenide vein-type mineralization (Fig. 1h). The deposit was intermittently mined for iron from the mid-18th century until 1938, and for gold starting in 1825^42^. Selenides form ore shoots, commonly a few centimeters across, within carbonate-hematite veins that truncate tectonically displaced blocks of hydrothermally reddened and/or bleached Silurian graptolite shale, as well as Devonian keratophyre and diabase of the Eastern Harz anticline^42^. A total of 18 selenide minerals have been reported from Tilkerode^43^, the type locality of naumannite [Ag_2_Se], eskebornite [CuFeSe_2_], tischendorfite [Pd_8_Hg_3_Se_9_] and tilkerodeite [Pd_2_HgSe_3_]^42–45^.

These selenide minerals are spatially associated with ankerite that postdates the predominant calcite- hematite mineralization. Both carbonate and hematite represent ~ 98% of the vein system^42^. According to Tischendorf^42^, there are two stages of selenide deposition: an early stage, characterized by berzelianite [Cu_2−x_Se], umangite [Cu_3_Se_2_], klockmannite [CuSe], and trogtalite [CoSe_2_]; and a late stage, characterized by tiemannite [HgSe], naumannite and clausthalite [PbSe]. Sulfide minerals, mostly pyrite and minor chalcopyrite, postdate early-stage but also late-stage selenide formation. All selenide and sulfide minerals are overprinted by native selenium, cerussite and iron-oxyhydroxide aggregates (limonite), attributed to late-stage weathering^42^.

## Samples and methods

Nine samples from Tilkerode’s selenide-bearing carbonate-hematite vein were obtained from the collection stored at, and with permission of, the ‘Landesamt für Geologie und Bergwesen Sachsen-Anhalt’. Sample aliquots provided material for reconnaissance whole-rock chemical analyses (for details see^46^, and polished thin and thick sections for reflected-light microscopy. In four of the polished thick sections (samples TK4 and TK6), framboidal aggregates of pyrite were identified using reflected-light microscopy. The pyrite composition of these aggregates, i.e., their Fe: S ratios (ESM Table 1), was determined by means of a Cameca SX FIVE field-emission electron microprobe at the Clausthal University of Technology, Germany. Subsequently, the framboidal aggregates and the surrounding subhedral pyrite were measured in situ for ^34^S/^32^S ratios at GFZ (Geoforschungszentrum Potsdam, Germany), using secondary ion mass spectrometry (SIMS; 1280-HR instrument). Fluid inclusions hosted in carbonate, spatially associated with selenide minerals, were investigated by Raman spectroscopy and microthermometry at the Georg-August University Göttingen, Germany. To place additional constraints on the timing of sulfide formation, carbonate from a highly oxidized calcite vein, post-dating pyrite formation, was dated in situ by laser ablation ‒ inductively coupled plasma ‒ mass spectrometry (LA‒ICP‒MS) at Karlsruhe Institute of Technology, Germany. Details about instrument conditions and analytical protocols are presented as Electronic Supplementary Material (ESM) together with detailed documentation of the SIMS spots and laser spots.

## Results

### Sulfur isotopic composition of framboidal aggregates and anhedral pyrite

**Fig. 2:**
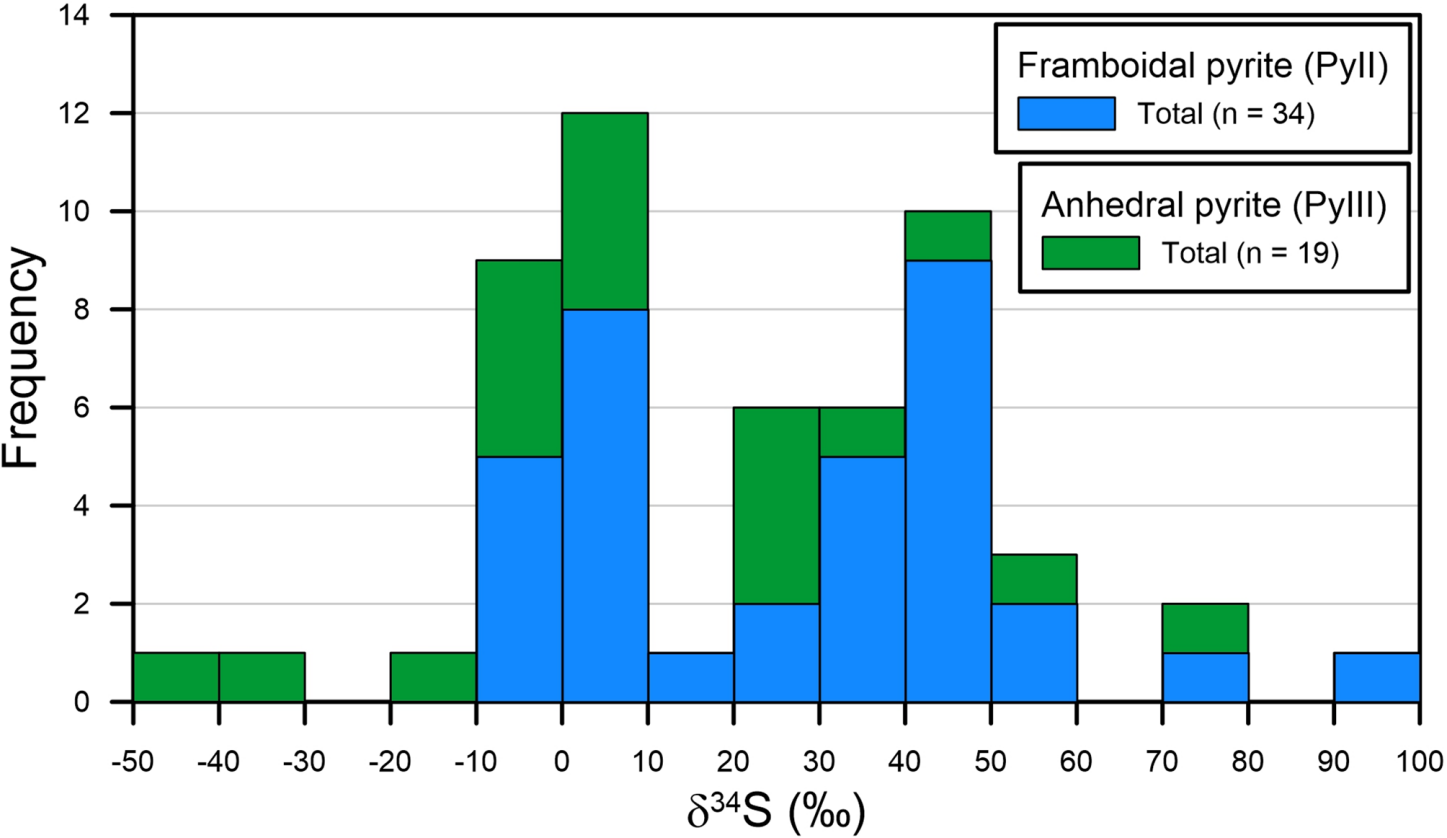
Histogram of ion-microprobe analyses of S isotopic compositions of framboidal PyII and anhedral PyIII.

Three generations of pyrite have been distinguished within the investigated samples. Pyrite I (PyI) occurs as massive crystals, < 50 μm across, included in chalcopyrite, commonly surrounded by clausthalite (Fig. 1a). Pyrite II (PyII) forms framboidal aggregates, < 20 μm across, in tabular voids of dissolved hematite (Fig. 1b, e, f), and in rhomboidal voids of dissolved carbonate (Fig. 1c). The aggregates are made up of densely packed framboids, each < 1 μm in diameter, with interstitial cement^6^. Pyrite III (PyIII) consists of massive/anhedral crystals and aggregates, up to > 100 μm across, commonly enclosing framboidal PyII, and locally embedded in a late, supergene Fe-oxyhydroxide-rich, limonite-like groundmass (Fig. 1d, g). SIMS analyses of framboidal PyII from two samples show extreme variations in S isotopes from ‒8.5 ± 0.6‰ to + 92.5 ± 3.9‰ δ^34^S (*n* = 34), with a total difference of Δ^34^S = 101‰. SIMS analyses of PyIII vary from ‒40.2 ± 0.5‰ to + 79.1 ± 0.3‰ δ^34^S (*n* = 19), with a total difference of Δ^34^S = 119‰ (Fig. 2; ESM Table S2).

### Carbonate-hosted fluid inclusions

**Fig. 3:**
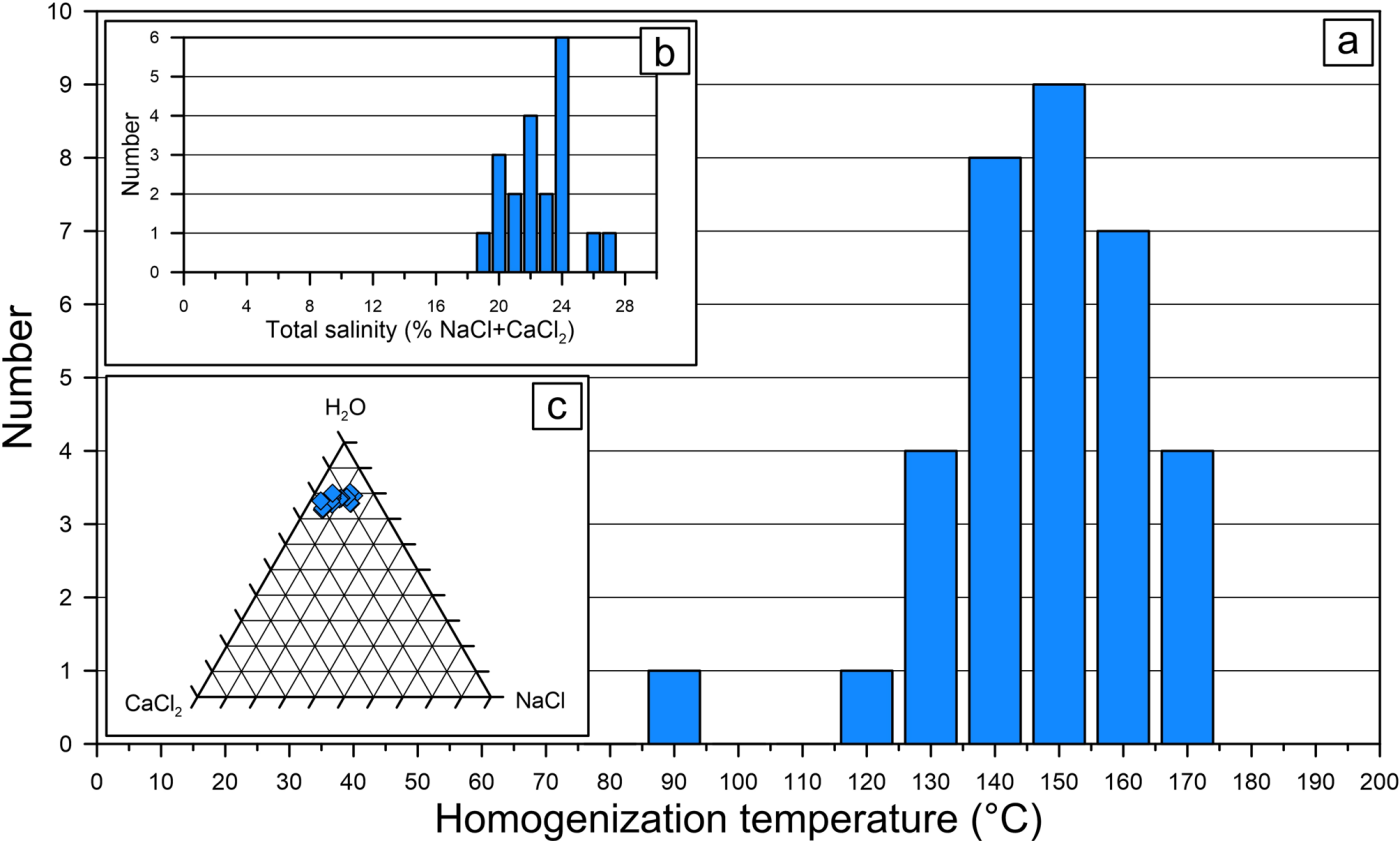
Microthermometric results of fluid inclusions in carbonate (ankerite). **a**: Histogram of homogenization temperature (T_h total_) of fluid inclusions. **b**: Histogram of total salinity of fluid inclusions. **c**: Brine H_2_O‒NaCl‒CaCl_2_ composition of fluid inclusions.

**Fig. 4:**
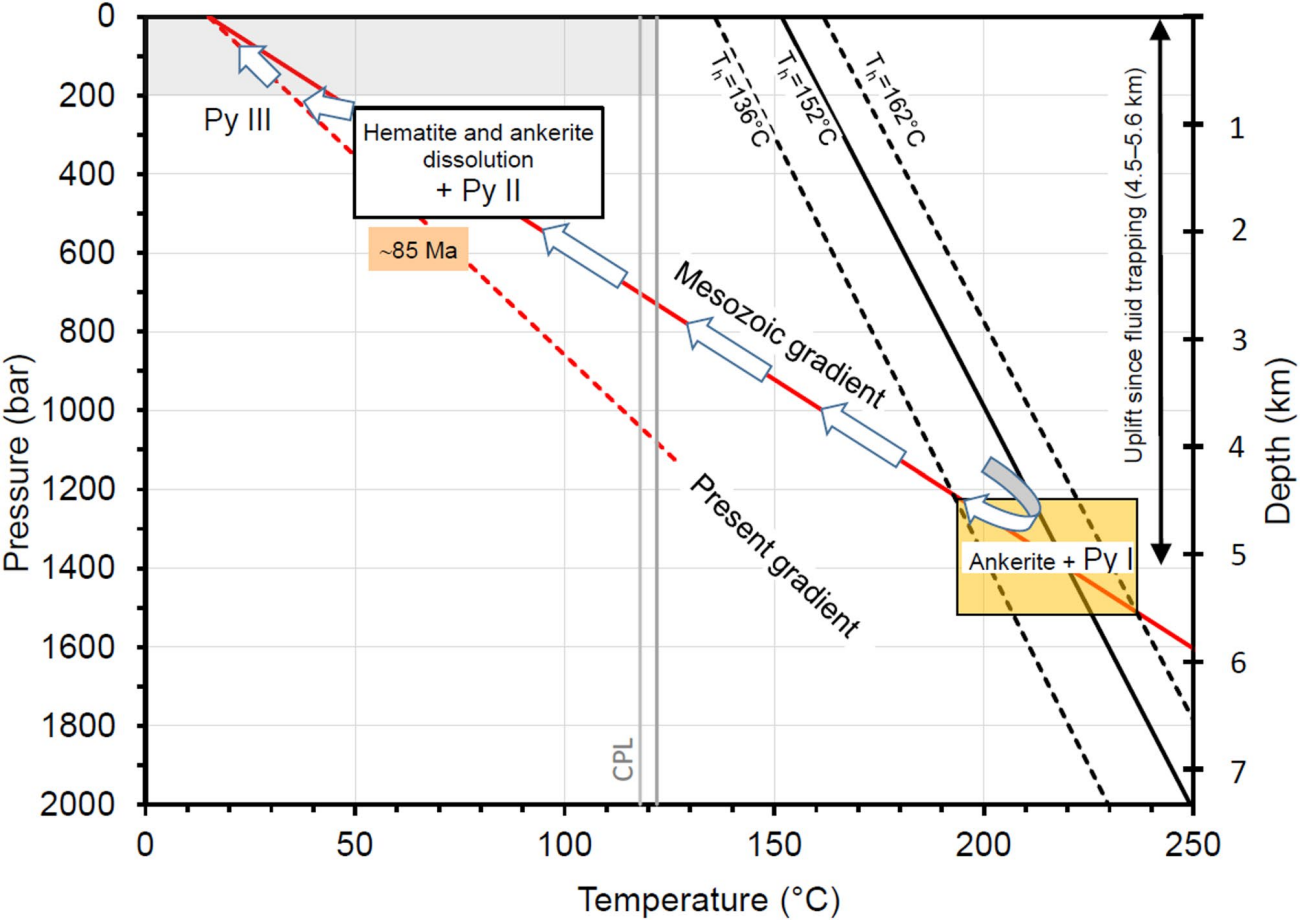
Diagram of temperature vs. pressure (depth), showing the intersection of isochores, calculated from microthermometric measurements of fluid inclusions in ankerite, with a Mesozoic lithostatic geothermal gradient of 40 °C/km and a present lithostatic gradient of 27 °C, calculated for a density of 2.73 g/cm^3^ (graptolite shale). The gray vertical lines marked with CPL indicate the high-temperature and high-pressure limit for possible cell proliferation (after^4^.

Transmitted-light microscopy and Raman spectroscopy results reveal that the white carbonate closely intergrown with clausthalite is ankerite, forming clear, non-luminescent crystals, as well as polycrystalline aggregates. The ankerite crystals commonly host two-phase (liquid+vapor) fluid inclusions at room temperature. Together, Raman spectroscopy and microthermometric measurements indicate a complex brine composition. Eutectic melting temperatures (T_e_) from − 53 to -51 °C point to CaCl_2_–NaCl-dominated solutions. Ice-melting temperatures (T_m ice_) indicate total salinity between 19 and 27% NaCl_eq_ (mass-percent equivalent in NaCl; Fig. 3b). The homogenization temperatures range from 89 to 174 °C (*n* = 34), though most analyses fall in a narrow range from 136 to 162 °C (*n* = 24; Fig. 3a). A list of all relevant temperatures for eutectic melting (T_e_), hydrohalite melting (T_m HH_), final ice melting (T_m ice_), and total homogenization (T_h total_) is presented in ESM Table S3. Combined data indicate a brine composition of 73–80% H_2_O, 3–14% NaCl and 7–21% CaCl_2_ (Fig. 3c). Isochores calculated for the fluid inclusions indicate fluid entrapment conditions of 200–250 °C at 1.4‒1.6 kbar (0.14‒0.16 GPa), which corresponds to crustal depths of 4.8 to 5.6 km by applying a lithostatic geothermal gradient of 40 °C/km (Fig. 4).

### Uranium-Pb dating of carbonate

**Fig. 5:**
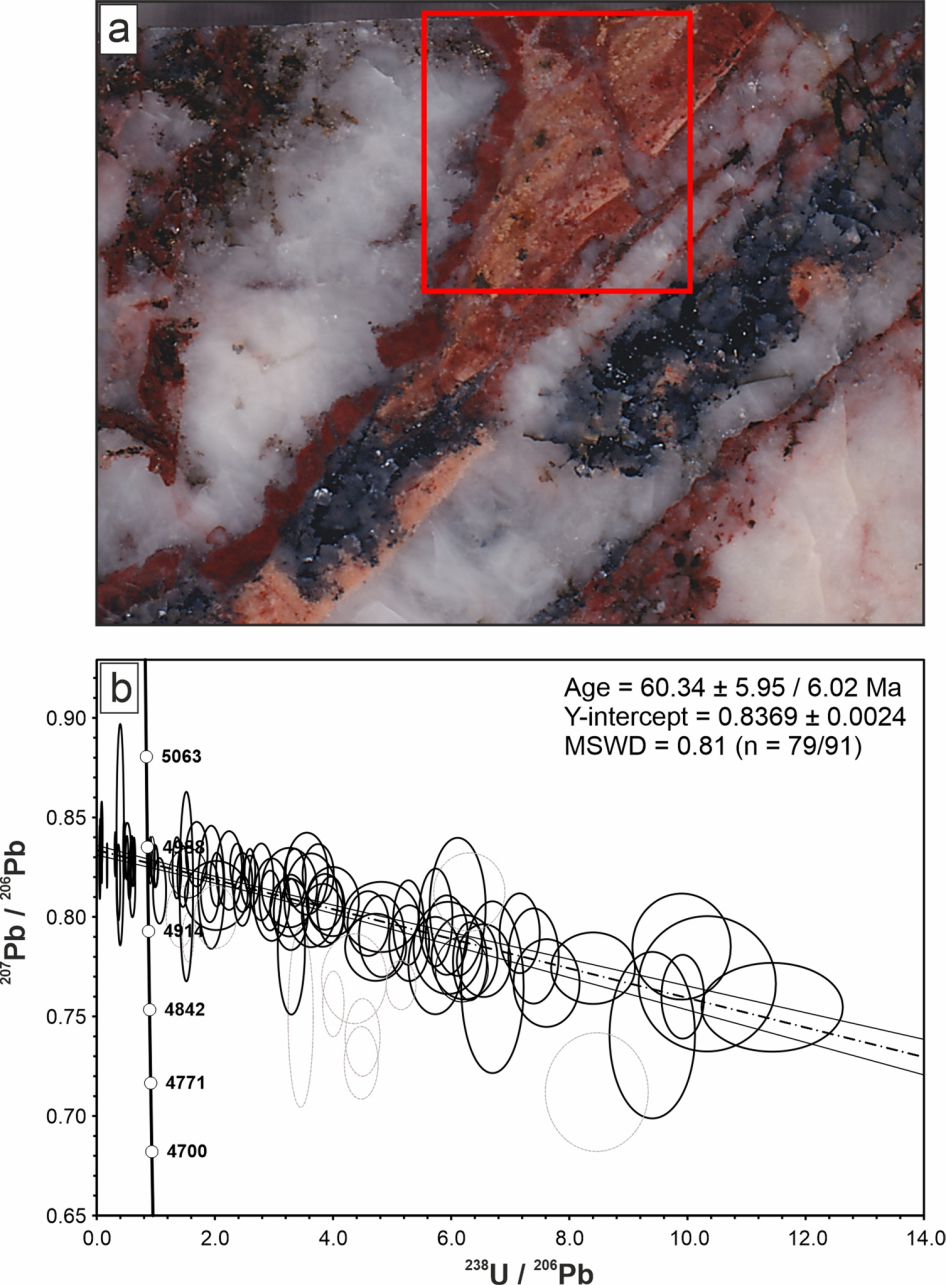
Results of U‒Pb carbonate dating by LA‒ICP‒MS. (a) Laser spot positions - indicated by the red square - within a highly oxidized carbonate domain, red dyed by iron oxide. The domain occurs in a typical white carbonate-hematite vein. (b) Tera-Wasserburg diagram with dating results.

To place constraints on the age of pyrite formation, calcite was dated from an oxidized domain of a carbonate vein, where the commonly white calcite has been dyed red by fine dispersed iron-oxide minerals (Fig. 5a). In situ U‒Pb analyses of 91 spots of this domain yielded an age of 60.3 ± 6.0 Ma (MSWD = 0.81; *n* = 79 of 91; Fig. 5b), indicating equilibration of the U‒Pb system under oxidizing conditions during the Early Tertiary.

## Discussion

The microthermometry results provide evidence that ankerite, coexisting with clausthalite, formed at temperatures of 200–250 °C (Fig. 4), well compatible with the conditions under which abiotic TSR operates, but at odds with the presence of the selenium-mineral umangite. This mineral was reported in the early-stage selenide assemblage as defined by Tischendorf^42^; however, it cannot be formed at temperatures above 112 °C^46^. We further note that TSR commonly results in δ^34^S shifts of less than 15‒20‰ relative to the source sulfate^14,16,17^, whereas the analyzed framboidal pyrite (PyII) and the anhedral pyrite (PyIII) from Tilkerode record an extreme range covering 133‰ δ^34^S. This suggests that both generations were mainly formed as a result of MSR rather than abiotic TSR. Extremely positive δ^34^S values, as analyzed from PyII (up to + 92.5 ± 3.9‰), but also from PyIII (up to + 79.1 ± 0.3‰), can only be reached by MSR under closed system conditions, when a limited amount of sulfate was progressively depleted at a slow cell-specific rate^7^. The δ^34^S values down to ‒8.5 ± 0.6‰ (PyII) and ‒40.2 ± 0.5‰ (PyIII) suggest that the environment changed during MSR, perhaps from closed- to more open-system conditions, and accompanied by an increase in sulfate availability.

We note that extreme S isotopic fractionation has already been recorded from different settings in the deep biosphere. For example, a total δ^34^S range from − 54‰ to + 132‰ δ^34^S, was found for pyrite (both with and without framboidal habit) in fractures transecting granitic rocks of the Baltic shield in Sweden^47^. These, currently most extreme ^34^S enrichment of fracture-hosted pyrite, sampled from the surface down to 1,660 m below sea level, has been attributed to a slow MSR rate in response to a combination of factors, comprising shortage of nutrient and/or electron donor, and Rayleigh fractionation of limited sulfate^47^. The latter has also been invoked to account for a similar ^34^S enrichment in the range of 70 to 100‰, found in framboidal pyrite in a low-temperature hydrothermal system of the Taupo Volcanic zone in New Zealand^48^, although temperature and depth for that vein system are not well established.

**Fig. 6:**
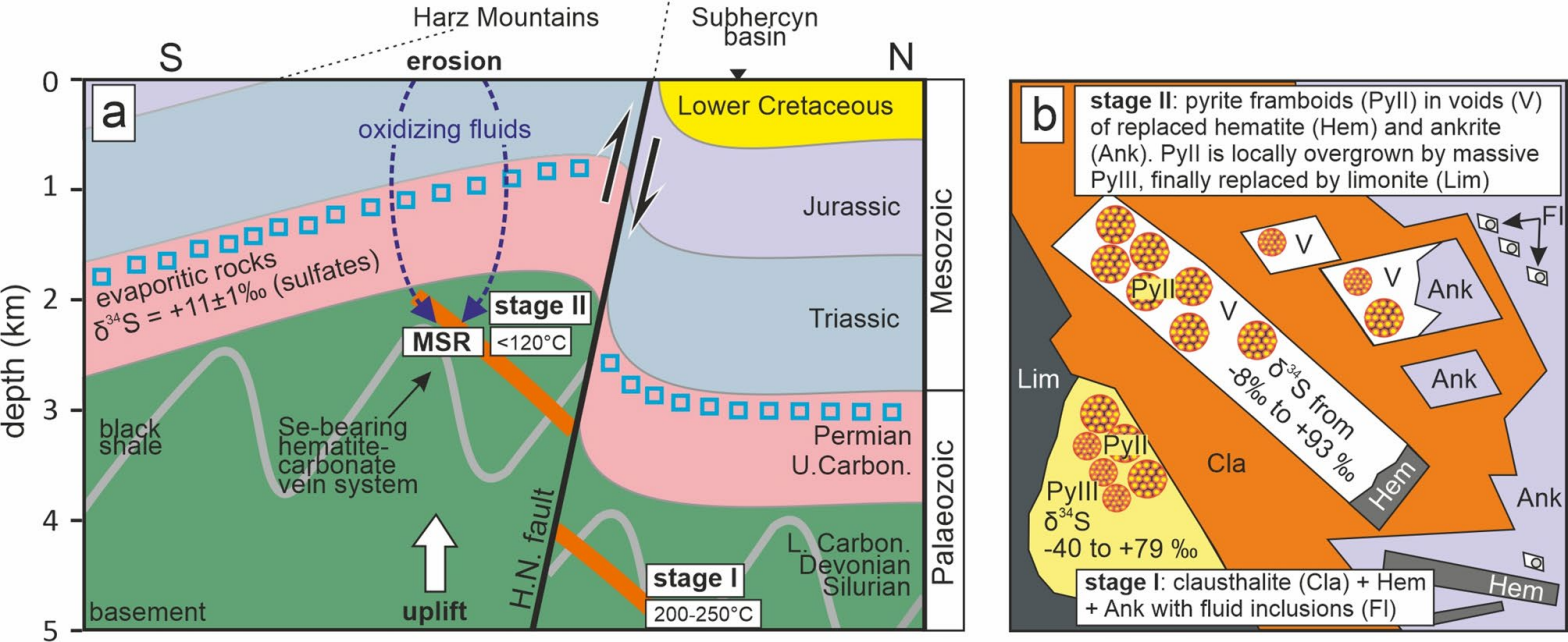
Two-stage model explaining the formation of framboidal pyrite with extreme S isotopic fractionation in Se-bearing hematite-carbonate veins of the Harz Mountains. **a**: Late Cretaceous reverse faulting along the Harz-Nordrand (H.N.) fault caused uplift and cooling of the Tilkerode vein system from **stage I** (~ 5 km depth, T = 200–250 °C) to **stage II** (~ 2 km depth, T < 120 °C), and downward percolation of oxidizing, sulfate-bearing fluids into the Paleozoic basement, resulting in microbial sulfate reduction (MSR). **b**: Stage I hematite and ankerite enclosed in clausthalite were partially dissolved during stage II and the voids filled with framboidal pyrite (PyII), showing extreme variations in δ^34^S values typical for MSR under closed-system conditions with very limited sulfate availability and slow metabolism. Subsequently, PyII was overgrown by anhedral PyIII, also showing extreme δ^34^S, albeit with more negative values, perhaps reflecting MSR under successively more open-system conditions. Late-stage limonite replacing PyIII indicates the final oxidation stage. Fluid inclusions in ankerite reflect conditions during stage I. Abbreviations: Ank – ankerite; Cla – clausthalite; Hem – hematite; Lim – limonite; Py – pyrite.

For the Tilkerode vein system neither the depth nor the temperature exceeded approximately 5 km and 200–250 °C, as indicated by our fluid inclusion data from ankerite (Fig. 4). These represent boundary conditions for the deep biosphere. However, the upper temperature limit for microbial life is 122 °C as determined by experiments^4^. This apparent discrepancy is solved by taking the tectono-thermal evolution of the Harz Mountains, hosting the Tilkerode vein system, into account. Results of apatite fission track and U‒Th(He) dating^49^ indicate rapid uplift and erosion of the Harz Mountains of about 2–3 km during the Late Cretaceous at ca. 85 Ma, traversing quickly through the partial annealing zone from > 120 °C to < 60 °C. Taking this into account, the fluid inclusion data from ankerite represent the P‒T conditions prior to Late Cretaceous uplift caused by reverse faulting along the “Harznordrand” fault (stage I in Fig. 6). In contrast, the framboidal PyII and anhedral PyIII were formed during a syn- to post-uplift period (stage II). Fast uplift not only caused rapid cooling from approximately 250 °C down to < 120 °C, but also the reactivation of existing vein systems. Furthermore, it enabled oxygenated, surface-derived aqueous fluids to percolate from overlying Paleozoic and Mesozoic cover rocks, including sulfate successions of the Zechstein sulfates with δ^34^S of 11 ± 1‰^50^, into the underlying crystalline basement, where these interacted with the pre-existing selenide-bearing carbonate-hematite vein system (Fig. 6). Coarse-grained laths of hematite, exceeding 50 μm in length, would then have been dissolved, resulting in tabular voids filled with framboidal PyII (Fig. 1b, c). In this context, it is worthwhile noting that coarse-grained hematite is chemically stable at near-surface conditions, but becomes increasingly soluble at temperatures above 50 °C in the presence of organic acids^51^, which also could account for the dissolution of ankerite, now forming voids filled with framboidal PyII (Fig. 1c). A potential source for the organic acids, but also for the methane needed to drive MSR at temperatures < 120 °C^28^, could have been the wall-rock graptolite shale^42^. In addition, it is likely that hematite, as a potential iron donator, was directly replaced during MSR, leading to the formation of framboidal PyII.

The sulfate needed for MSR was either transported with downward percolating oxidizing surface fluids, e.g., from overlying Zechstein sulfate layers, or resulted from the interaction of Se-bearing oxidizing fluids with existing sulfides, such as PyI, chalcopyrite, or even galena [PbS]. Galena perhaps was extensively replaced by clausthalite [PbSe]^52^, and the released sulfate then available for metabolization by microorganisms.

Microorganisms in the deep biosphere are expected to metabolize 10^4^- to 10^6^-fold slower than those in nutrient-rich environments^53^. Such slow MSR rates, in addition to a limited sulfate availability, are ideal conditions for MSR that yields framboidal pyrite with extremely positive δ^34^S values. Lower δ^34^S down to ‒8‰ measured on the framboidal PyII could be explained by temporal and spatial variations in bacterial activity and sulfate availability in the Tilkerode vein system. The lowest δ^34^S of ‒41‰ estimated on PyIII perhaps also resulted from MSR, but in an open-system environment, which was reached during ongoing uplift and erosion of the Harz Mountains, allowing for a more voluminous infiltration of surface fluids at lower temperatures, and higher bacterial activity. We note that the lowest δ^34^S values overlap those measured on pyrite in marine black shales of the Lower Permian Kupferschiefer^54^. The high and variable δ^34^S values of the anhedral PyIII might also result from partial reworking of the pre-existing framboidal PyII, in line with observed overgrowth microfabrics (Fig. 1d). Overgrowths relationships further indicate, that all MSR activities occurred prior to the formation of limonite, which represents a very late-stage phase of fluid alteration related to the final exhumation of the Harz Mountains during the Cenozoic^55^. Our new results of carbonate dating suggest that formation of framboidal PyII and anhedral PyIII occurred prior to the Early Tertiary at 60 ± 6 Ma, when the U‒Pb system of carbonate veins became equilibrated under oxidizing conditions (Fig. 5). At this time, the Tilkerode vein system was still at 1–2 km depth (T = 30–60 °C), as is constrained by U‒Th(He) modelling results carried out for the Paleozoic basement of the Eastern Harz Mountains^49^.

## Conclusion

Tilkerode’s framboidal pyrite shows an extreme diversity in δ^34^S values, extending from ‒ 8‰ to + 93‰ δ^34^S. The extreme ^34^S enrichment is attributed to MSR in an essentially closed, albeit toxic system of limited sulfate availability. Pyrite framboids (PyII) formed during hematite dissolution at depths of 0.8–1.8 km, after rapid uplift and cooling of the Tilkerode vein system from ~ 5 to ~ 2 km depth, and from 220 °C to < 120 °C during the Cretaceous. Anhedral pyrite (PyIII), also with extreme δ^34^S values from ‒ 40 to + 79‰ formed afterwards under successively more open-system conditions, related to ongoing uplift and cooling until the Early Tertiary, but prior to highly oxygenated conditions reflected by late-stage limonite. Tilkerode’s vein system records the deep-biosphere transition from low-temperature hydrothermal conditions at < 120 °C to a near-surface weathering environment.

## Supplementary Information

Below is the link to the electronic supplementary material.

Supplementary Material 1

Supplementary Material 2

Supplementary Material 3

Supplementary Material 4

Supplementary Material 5

## Data Availability

The authors declare that the data supporting the findings in this study are available within the paper and its supplementary information files.

## References

[1] Moser, D. P. et al. Temporal shifts in the geochemistry and microbial community structure of an ultradeep mine borehole following isolation. *Geomicrobiol. J.* **20**, 517–548 (2003).

[2] Li, L. et al. Sulfur mass-independent fractionation in subsurface fracture waters indicates a long-standing sulfur cycling in Precambrian rocks. *Nat. Commun.* **7**, 13252. 10.1038/ncomms13252 (2016). 27807346 10.1038/ncomms13252 PMC5095282

[3] Parnell, J. & McMahon, S. Physical and chemical controls on habitats for life in the deep subsurface beneath continents and ice. *Phil Trans. R Soc. A*. **374**, 20140293. 10.1098/rsta.2014.0293 (2016). 26667907 10.1098/rsta.2014.0293 PMC4685966

[4] Takai, K. Cell proliferation at 122°C and isotopically heavy CH_4_ production by a hyperthermophilic methanogen under high-pressure cultivation. *Proc. Natl. Acad. Sci. U.S.A.* **105**, 10949–10954 (2008). 10.1073/pnas.0712334105 PMC2490668 18664583

[5] Simon, G., Kesler, S. E. & Essene, E. J. Phase relations among selenides, sulfides, tellurides, and oxides: II. Applications to selenide-bearing ore deposits. *Econ. Geol.* **92**, 468–484 (1997).

[6] Yue, L., Jiao, Y., Fayek, M., Wu, L. & Rong, H. Micromorphologies and sulfur isotopic compositions of pyrite in sandstone-hosted uranium deposits: a review and implications for ore genesis. *Ore Geol. Rev.* **139**, 104512 (2021).

[7] Hough, G., Swapp, S., Frost, C. & Fayek, M. Sulfur isotopes in biogenically and abiogenically derived uranium roll-front deposits. *Econ. Geol.* **114**, 353–372 (2019).

[8] Bonnetti, C., Zhou, L., Riegler, T., Brugger, J. & Fairclough, M. Large S isotope and trace element fractionations in pyrite of uranium roll front systems result from internally-driven biogeochemical cycle. *Geochim. Cosmochim. Acta*. **282**, 113–132 (2020).

[9] Jørgensen, B. B., Isaksen, M. F. & Jannasch, H. W. Bacterial sulfate reduction above 100°C in deep-sea hydrothermal vent sediments. *Science* **258**, 1756–1757. 10.1126/science.258.5089.175 (1992). 17831655 10.1126/science.258.5089.1756

[10] Cai, C., Li, H., Li, K. & Wang, D. Thermochemical sulfate reduction in sedimentary basins and beyond: a review. *Chem. Geol.* **607**, 121018 (2022).

[11] Machel, H. G. Some aspects of diagenetic sulphate-hydrocarbon redox reactions. *Geol. Soc. Spec. Publ.* **36**, 15–28 (1987).

[12] Machel, H. G. Relationships between sulphate reducing and oxidation of organic compounds to carbonate diagenesis, hydrocarbon accumulations, salt domes, and metal sulphide deposits. *Carbonates Evaporites*. **4**, 137–151 (1989).

[13] Machel, H. G. Bacterial and thermochemical sulfate reduction in diagenetic settings – old and new insights. *Sediment. Geol.* **140**, 143–175 (2001).

[14] Machel, H. G., Krouse, H. R. & Sassen, R. Products and distinguishing criteria of bacterial and thermochemical sulfate reduction. *Appl. Geochem.* **10**, 373–389 (1995).

[15] Nöth, S. High H_2_S contents and other effects of thermochemical sulfate reduction in deeply buried carbonate reservoirs: a review. *Geol. Rundsch*. **86**, 275–287 (1997).

[16] Machel, H. G. &amp; Foght, J. Products and depth limits of microbial activity in petroliferous subsurface settings. In *Microbial Sediments* (eds. Riding, R. E. &amp; Awramik, S. M.) 105–120 (Springer, 2000).

[17] Kiyosu, Y. & Krouse, H. R. The role of organic acid in the abiogenic reduction of sulfate and the sulfur isotope effect. *Geochem. J.* **24**, 21–27 (1990).

[18] Trudinger, P. A., Chambers, L. A. & Smith, J. W. Low-temperature sulphate reduction: biological versus abiological. *Can. J. Earth Sci.* **22**, 1910–1918 (1985).

[19] Kesler, S. E. et al. Role of crude oil in the genesis of Mississippi Valley-type deposits: evidence from the Cincinnati arch. *Geol* **22**, 609–612 (1994).

[20] Spangenberg, J. E., Fontboté, L. & Macko, S. A. An evaluation of the inorganic and organic geochemistry of the San Vicente Mississippi Valley-type zinc-lead district, Central Peru: implications for ore fluid composition, mixing processes, and sulfate reduction. *Econ. Geol.* **94**, 1067–1092 (1999).

[21] Ireland, T., Large, R. R., McGoldrick, P. & Blake, M. Spatial distribution patterns of sulfur isotopes, nodular carbonate, and ore textures in the McArthur River (HYC) Zn-Pb-Ag deposit, Northern Territory, Australia. *Econ. Geol.* **99**, 1687–1709 (2004).

[22] Huston, D. L., Stevens, B., Southgate, P. N., Muhling, P. & Wyborn, L. Australian Zn-Pb-Ag ore-forming systems: a review and analysis. *Econ. Geol.* **101**, 1117–1157 (2006).

[23] Basuki, N. I., Taylor, B. E. & Spooner, E. T. C. Sulfur isotope evidence for thermochemical reduction of dissolved sulfate in Mississippi Valley-type zinc-lead mineralization, Bongara area, northern Peru. *Econ. Geol.* **103**, 783–799 (2008).

[24] Henjes-Kunst, E., Raith, J. G. & Boyce, A. J. Micro-scale sulfur isotope and chemical variations in sphalerite form the Bleiberg Pb-Zn depsit, Eastern Alps, Austria. *Ore Geol. Rev.* **90**, 52–62 (2017).

[25] Zhou, J. X. et al. New insights into the metallogeny of MVT Zn-Pb deposits: a case study from the Nayongzhi in South China, using field data, fluid compositions, and in situ S-Pb isotopes. *Am. Mineral.* **103**, 91–108 (2018).

[26] Zhu, C. et al. Variations in Zn and S isotope chemistry of sedimentary sphalerite, Wusihe Zn-Pb deposit, Sichuan province, China. *Ore Geol. Rev.* **95**, 639–648 (2018).

[27] Kaplan, I. R. & Hulston, J. R. The isotopic abundance and content of sulfur in meteorites. *Geochim. Cosmochim. Acta*. **30**, 479–496 (1966).

[28] Canfield, D. E. Biogeochemistry of sulfur isotopes. *Rev. Mineral. Geochem.* **43**, 607–636 (2001).

[29] Canfield, D. E. Isotope fractionation by natural populations of sulfate-reducing bacteria. *Geochim. Cosmochim. Acta*. **65**, 1117–1124 (2001).

[30] Gomes, M. L., Fike, D. A., Bergmann, K. D., Jones, C. & Knoll, A. H. Environmental insights from high-resolution (SIMS) sulfur isotope analyses of sulfides in Proterozoic microbialites with diverse mat textures. *Geobiol* **16**, 17–34 (2016). 10.1111/gbi.12265 29047210

[31] Ohmoto, H. & Goldhaber, M. B. Sulfur and carbon isotopes. In *Geochemistry of Hydrothermal Ore Deposits* (ed. Barnes, H. L.) 517–611 (Wiley, 1997).

[32] Lin, Z. et al. How sulfate-driven anaerobic oxidation of methane affects the sulfur isotopic composition of pyrite: a SIMS study from the South China Sea. *Chem. Geol.* **440**, 26–41 (2016).

[33] von Eynatten, H., Kley, J., Dunkl, I., Hoffmann, V. E. & Simon, A. Late Cretaceous to Paleogene exhumation in central Europe – localized inversion vs. large-scale domal uplift. *Solid Earth*. **12**, 935–958 (2021).

[34] Kley, A. Timing and spatial patterns of Cretaceous and Cenozoic inversion in the Southern Permian basin in Mesozoic resource potential in the Southern Permian Basin. *Geol. Soc. Spec. Publ.* **469**, 19–31 (2018).

[35] Brink, H. J. The crustal structure around the Harz Mountains (Germany): review and analysis. *Z. Ges Geowiss.* **162/3**, 235–250 (2011).

[36] von Eynatten, H., Voigt, T., Meier, A., Franzke, H. J. & Gaupp, R. Provenance of Cretaceous clastics in the Subhercynian Basin: constraints to exhumation of the Harz Mountains and timing of inversion tectonics in Central Europe. *Int. J. Earth Sci. (Geol Rund)*. **97**, 1315–1330 (2008).

[37] MacLean, L. C. W. et al. A high-resolution chemical and structural study of framboidal pyrite formed within a low-temperature bacterial biofilm. *Geobiol* **6**, 471–480 (2008). 10.1111/j.1472-4669.2008.00174.x 19076638

[38] Harithsa, S., Kerkar, S. & Bharathi, P. A. L. Mercury and lead tolerance in hypersaline sulfate-reducing bacteria. *Mar. Pollut. Bull.* **44**, 726–732 (2002). 12269474 10.1016/s0025-326x(02)00174-1

[39] Barton, L. L., Tomei-Torres, F. A., Xu, H. &amp; Zocco, T. Metabolism of metals and metalloids by the sulfate-reducing bacteria in Bacteria-metal interactions (ed Saffarini, D.) 57–83 (Springer, 2015).

[40] Golysheva, A. A., Litvinenko, L. V. & Ivshina, I. B. Diversity of mercury-tolerant microorganisms. *Microorganisms* **13**, 1350 (2016). 10.3390/microorganisms13061350 PMC12195606 40572238

[41] Pant, R., Dhyani, N., Arya, P., Tripathy, S. & Gupta, A. Molecular mechanisms of mercury toxicity and tolerance in microbes in Mercury toxicity mitigation: sustainable nexus approach (ed. Kumar N). 10.1007/978-3-031-48817-7_7 (2024).

[42] Tischendorf, G. Zur Genesis einiger Selenidvorkommen, insbesondere von Tilkerode im Harz. *Freiberger Forschungshefte C*. **69**, 1–168 (1959).

[43] Ma, C., Förster, H. J. & Grundmann, G. Tilkerodeite Pd_2_HgSe_3_, a new platinum-group mineral from Tilkerode, Harz Mountains. *Ger. Crystals*. **10**, 687. 10.3390/cryst10080687 (2020).

[44] Ramdohr, P., Neue Erzmineralien *Fortschr. Min.* **28**, 69–70 (1949).

[45] Stanley, C. J., Criddle, A. J., Förster, H. J. & Roberts, A. C. Tischendorfite Pd_8_Hg_3_Se_9_, a new mineral species from Tilkerode, Harz Mountains, Germany. *Can. Mineral.* **40**, 739–745 (2002).

[46] Lohmeier, S., Cabral, A. R., Ehling, B. C. & Zeh, A. Germanium and precious metals (Ag-Au-Pt-Pd) at low temperature: the hematite-carbonate-selenide vein system of Tilkerode, Harz Mountains, Germany. *Min. Deposita*. **58**, 1371–1379 (2023).

[47] Drake, H. et al. Unprecedented ^34^S-enrichment of pyrite formed following microbial sulfate reduction in fractured crystalline rocks. *Geobiol* **16**, 556–574 (2018). 10.1111/gbi.12297 29947123

[48] Wang, L. et al. Extreme sulfur isotope fractionation of hydrothermal auriferous pyrites from the SW fringe of the Taupo Volcanic Zone, New Zealand: implications for epithermal gold exploration. *Results Geochem.* **3**, 100009. 10.1016/j.ringeo.2021.100009 (2021).

[49] von Eynatten, K. et al. Late Cretaceous exhumation and uplift of the Harz Mountains, Germany: a multi-method thermochronological approach. *Int. J. Earth Sci. (Geol Rundsch)*. **108**, 2097–2111 (2019).

[50] Kampschulte, A., Buhl, D. & Strauss, H. The sulfur and strontium isotopic composition of Permian evaporites from the Zechstein basin, northern Germany. *Geol. Rundschau*. **87**, 192–199 (1998).

[51] Taxiarchou, M., Panias, D., Douni, I., Paspaliaris, I. & Kontopoulos, A. Dissolution of hematite in acidic oxalate solutions. *Hydrometallurgy* **44**, 287–299 (1997).

[52] Förster, H. J. Mineralogy of the U–Se-polymetallic deposit Niederschlema–Alberoda, Erzgebirge, Germany. IV. The continuous clausthalite–galena solid-solution series. *N. Jb. Miner. Abh.* **181**, 125–134 (2005).

[53] Hoehler, T. M. & Jørgensen, B. B. Microbial life under extreme energy limitation. *Nat. Rev.* **11**, 83–94 (2013). 10.1038/nrmicro2939 23321532

[54] Magnall, J., Liu, Y., Gleeson, S. & Rocholl, A. The origin of the Kupferschiefer mineralization (eastern Germany): constraints from petrography and analysis of stable sulfur isotopes of pyrite and chalcopyrite. *EGU General Assembly* Vienna, Austria, EGU-24-10634. 10.5194/egusphere-egu24-10634 (2024).

[55] König, W., Köthe, A. & Bitz, I. The marine influence of the Subhercynian basin and the high level planation surfaces in the central Harz Mountains in the Oligocene – stratigraphical and sedimentological analysis of Tertiary sand deposits. *Z. Geol. Wiss* **39**, 387–431 (2011).

