# Supplementary material for: Microbial life in deep-seated selenide veins reflected by extreme δ34S fractionation of framboidal pyrite
Source: Sci Rep. 2026 Jun 26;16:19627. doi: 10.1038/s41598-026-59857-1 (PMC13309615; doi:10.1038/s41598-026-59857-1)
Supplement: Supplementary file 1 — Supplementary Material 1 [file 41598_2026_59857_MOESM1_ESM.pdf]

Electronic Supplementary Material A – Analytical Details

Samples

Nine samples of selenide-bearing carbonate-hematite vein were taken for reconnaissance whole-rock chemical analyses (for details see [47]) of which polished thin and thick sections have been prepared. In two of the polished thick sections framboidal aggregates of pyrite were observed using reflected-light microscopy. Sample TK4 is from the southern winze and sample TK6 from the 'Goldschacht' (gold shaft) of the Eskeborner Berg underground workings. Subsequently, remains of these two samples were crushed and selenide concentrates were hand-picked and then mounted in epoxy resin.

Electron probe microanalyses (EPMA)

Pyrite aggregates have been measured by means of a Cameca SX FIVE field-emission electron microprobe, housed at Clausthal University of Technology (Germany; **ESMA-Table S1**). The instrument was operated at 15 kV and 30 nA in the wavelength-dispersive mode using the following X-ray lines, spectrometer crystals and reference materials (in parentheses): Co $K\alpha$ LLIF (pentlandite), Fe $K\alpha$ LIF (chalcopyrite), Ni $K\alpha$ LLIF (pentlandite), S $K\alpha$ PET (sphalerite), Sb $L\alpha$ LPET (stibnite), Se $L\alpha$ LTAP (BiSe). Beam diameter was fully focused. Peak measuring times were 10 s for Fe, S, Sb, Se, 20 s for Ni, and 30 s for Co; single-sided background side measuring times were 5 s for Fe, S, Sb, Se, 10 s for Ni, and 15 s for Co. Background measurements were as follows: Co (-/500), Fe (-500/-), Ni (-/500), S (-700/600), Sb (-800/-), Se (-/800). This analytical setting was tested on the reference materials used for calibration, the measurements of which gave no detectable X-ray line interference that could have influenced the output of quantitative measurement.

ESMA-Table S1. Results of electron probe microanalyses (EPMA) of framboidal pyrite II.

No.	S [wt%]	Fe [wt%]	Sb [wt%]	Se [wt%]	Co [wt%]	Ni [wt%]	Total [wt%]
1	52.09	46.27	0.19	<0.07	<0.05	<0.08	98.55
2	52.66	47.40	0.16	<0.07	<0.05	<0.08	100.21
3	52.83	46.86	<0.08	<0.07	<0.05	<0.08	99.70
4	52.62	46.57	0.20	<0.07	<0.05	<0.08	99.39
5	53.17	47.37	0.09	<0.07	<0.05	<0.08	100.63
6	52.78	46.49	0.18	<0.07	<0.05	<0.08	99.45
7	52.23	46.55	0.10	<0.07	<0.05	<0.08	98.87

Secondary-ion mass spectrometry (SIMS)

SIMS analyses on four selected samples from the Tilkerode carbonate-hematite vein mineralization were carried out at Geoforschungszentrum Potsdam (Germany) using a Cameca 1280-HR instrument in Potsdam in. A nominally 2.0-nA, 10-keV $^{133}Cs^{+}$ primary ion beam was employed for all microanalyses. Raw measured $^{34}S/^{32}S$ ratios were converted to the delta notation $\delta^{34}S$ by normalising to Vienna Cañon Diablo Troilite [$(^{34}S/^{32}S)_{VCDT}$ = 0.044163[57]**Fehler! Verweisquelle konnte nicht gefunden werden.**]. Instrument drift was monitored by repeated analyses of Balmat pyrite [$(^{34}S/^{32}S)_{B}$ = 0.044795; $\delta^{34}S$ = 14.3[58]], which gave a repeatability of 0.12‰ (0.0444519 ± 0.0000055; 1s; n = 24) on the first day of measurement and a repeatability of 0.08‰ (0.0445733 ± 0.0000034; 1s; n =13) on the second day of measurement. Drift correction was applied to the $^{34}S/^{32}S$ ratios obtained from the first day. Results are in **ESMA-Table S2**.

Details

Sample were prepared as rock chips that were embedded in 2 component cold set epoxy which was then ground and polished to a surface quality of <1 µm over the entirety of the 1-inch diameter mount. Each mount contained around 15 such chips with sizes between 5 and 10 mm. Each mount was studied under reflected light in order to identify the optimal measurement locations for our SIMS sulfur isotope determinations, care being taken to select locations that were within 8 mm of the center of the round mount whenever possible.

The mounts were individually cleaned ultrasonically in high-purity ethanol followed by argon sputter coating that deposited a 35 nm thick film of a high-purity gold film across the mount's surface, this film is needed for suppressing electrical charging during the subsequent SIMS isotope ratio determinations. Next, the mounts were imaged in their entirety using a Nikon Eclipse scanning optical microscope (in ESMC) prior to placing the mounts in the SIMS instrument's vacuum system. The samples were allowed to outgas for several days prior to beginning our measurements. Our $\delta^{34}S$ determinations were conducted over a consecutive 2-day period; as the Balmat reference pyrite was not included in the actual sample mounts we were compelled to do periodic sample-RM mount swaps in order to check the stability of the SIMS instrument's fractionation. During the first measurement day the intrinsic instrumental mass fractionation (IMF) drifted by 1‰ over our seven hours of data collection; this was accounted for with a linear best-fit correction in time vs. $^{34}S^{-}/^{32}S^{-}$ measurement space. On the second day the IMF was constant throughout the four hours of data acquisition. Our raw and processed data are reported in ESMD. BSE images showing the typical craters conducted on pyrite framboids are provided in ESMB.

Our actual SIMS analyses employed a 200 to 400 pA, mass filtered $^{133}Cs^{+}$ primary beam using Gaussian illumination and with a beam size of ca 3 * 4 µm at the sample surface; the total impact energy was 20 keV. Each $^{34}S^{-}/^{32}S^{-}$ determination was preceded by a 70 second (s) presputtering using a 10 *10 µm raster in order to remove locally the gold coat and for establishing equilibrium sputtering conditions. Following the presputtering the beam raster was turned off and automatic centering routines were

conducted for X and Y on the field aperture and for X on the mass spectrometer's entrance slit. We did not conduct any centering routine for the 40 V wide energy window.

SIMS data collection was done in static multi-collection mode where each determination consisted of 20 quasi-independent integrations each lasting 4 s. The $^{32}S^-$ signal was collected on a Faraday cup positioned at the L2' position in conjunction with a 10^{10} Ω amplifier. The ^{34}S- signal was collected on a second Faraday cup that was positioned behind the central mono-collection slit. The mono-collection slit was closed to 249 µm width, providing a mass resolution of M/ΔM ≈ 5000, which is more than adequate for eliminating both the $^{33}S^1H^-$ and $^{16}O^{18}O^-$ isobaric interferences. The central Faraday cup employed a 10^{12} Ω amplifier. The count rate on the $^{32}S^-$ mass station was typically between 1.5 and 2.5 * 10^8 ion per s. A single analysis, including presputtering, automatic centering routines and data acquisition took around 3 minutes.

Our SIMS IMF value was calibrated using the Balmat pyrite RM, which has an assigned $\delta^{34}S_{CDT}$ value of 14.3‰[58]. For calibrating the zero point of the Canyon Diablo Troilite isotope ratio scale we used the value of[57] of $^{34}S/^{32}S$ = 0.044163. For the first measurement day, after correcting for a linear drift, we achieved a repeatability of ± 0.08‰ (1s; n = 24) and for the second measurement day we achieved a repeatability of ± 0.12‰ (1s; n = 13). It is known that the Balmat pyrite is isotopic heterogenic with a real range in its $^{34}S/^{32}S$ value when measured at the micrometer scale[59]. Roughly 90% of the material belongs to the main population with a tail of isotopically lighter results tailing up to 3‰ lighter from the main population. On both analysis days we included two distinct pieces of Balmat in our calibrations; as these both yielded consistent $^{34}S^-/^{32}S^-$ values, we conclude that our calibrations must have been collected on material representing the main population within the Balmat pyrite. It therefore seems reasonable to conclude that our results on the Tilkerode SIMS mounts should be true to within 1‰, as based on the limitation of our reference pyrite, and should be precise to ± 0.2‰ (1s), as based on our repeatability on that reference pyrite.

ESMA-Table S2. Results of secondary-ion mass spectrometry (SIMS) of framboidal pyrite II and anhedral pyrite III.

No.	Sample	$^{32}S^-$	$^{34}S/^{32}S$ measured	1 SE	$^{34}S/^{32}S$ drift corrected	$^{34}S/^{32}S$ IMF corrected	$\delta^{34}S$ (V-CDT)	1SE internal	Pyrite type
1	TK4	1.31E+08	0.0438734	0.47	0.0438621	0.0442002	0.8	0.47	framboidal
2	TK4	1.63E+08	0.0439460	0.40	0.0439342	0.0442728	2.5	0.40	framboidal
3	TK4	2.00E+08	0.0438877	0.91	0.0438754	0.0442136	1.1	0.91	framboidal
4	TK4	2.44E+08	0.0437914	0.24	0.0437781	0.0441156	-1.1	0.24	framboidal
5	TK4	2.31E+08	0.0438859	0.18	0.0438720	0.0442102	1.1	0.18	framboidal
6	TK4	2.23E+08	0.0462838	1.17	0.0462694	0.0466260	55.8	1.17	framboidal
7	TK4	2.00E+08	0.0458956	0.30	0.0458807	0.0462344	46.9	0.30	framboidal
8	TK4	1.85E+08	0.0438990	0.35	0.0438831	0.0442214	1.3	0.35	framboidal
9	TK4	3.20E+07	0.0434684	0.61	0.0434519	0.0437869	-8.5	0.61	framboidal
10	TK4	2.26E+08	0.0438500	0.26	0.0438328	0.0441707	0.2	0.26	framboidal

ESMA-Table S2. (continued).

No.	Sample	$^{32}S^-$	$^{34}S/^{32}S$ measured	1 SE	$^{34}S/^{32}S$ drift corrected	$^{34}S/^{32}S$ IMF corrected	$\delta^{34}S$ (V-CDT)	1SE internal	Pyrite type
11	TK4	2.06E+08	0.0437372	0.14	0.0437191	0.0440561	-2.4	0.14	framboidal
12	TK4	2.51E+08	0.0437221	0.15	0.0437035	0.0440403	-2.8	0.15	framboidal
13	TK4	1.53E+08	0.0438337	0.73	0.0438141	0.0441518	-0.3	0.73	framboidal
14	TK4	1.44E+08	0.0438651	0.48	0.0438448	0.0441828	0.4	0.48	framboidal
15	TK4	2.56E+08	0.0458023	0.72	0.0457939	0.0461469	44.9	0.72	framboidal
16	TK4	2.51E+08	0.0459048	0.58	0.0458958	0.0462496	47.2	0.58	framboidal
17	TK4	2.81E+08	0.0420737	0.53	0.0420638	0.0423881	-40.2	0.53	subhedral
18	TK4	2.19E+08	0.0421049	0.29	0.0420943	0.0424187	-39.5	0.29	subhedral
19	TK4	1.61E+08	0.0436461	0.26	0.0436213	0.0439576	-4.7	0.26	subhedral
20	TK4	1.85E+08	0.0439945	0.40	0.0439702	0.0443091	3.3	0.40	subhedral
21	TK4	2.03E+08	0.0458666	1.28	0.0458331	0.0461864	45.8	1.28	framboidal
22	TK4	1.68E+08	0.0455939	0.70	0.0455600	0.0459112	39.6	0.70	framboidal
23	TK4	1.83E+08	0.0452092	0.20	0.0451748	0.0455230	30.8	0.20	framboidal
24	TK4	2.05E+08	0.0447625	0.25	0.0447264	0.0450712	20.6	0.25	framboidal
25	TK4	2.19E+08	0.0471013	0.26	0.0470641	0.0474269	73.9	0.26	framboidal
26	TK4	1.52E+08	0.0459578	0.34	0.0459195	0.0462735	47.8	0.34	framboidal
27	TK4	1.47E+08	0.0452977	0.65	0.0452586	0.0456074	32.7	0.65	framboidal
28	TK4	1.57E+08	0.0455960	0.27	0.0455542	0.0459053	39.5	0.27	framboidal
29	TK4	1.83E+08	0.0459605	0.44	0.0459178	0.0462717	47.7	0.44	framboidal
30	TK4	9.23E+08	0.0457226	0.82	0.0456788	0.0460310	42.3	0.82	framboidal
31	TK4	1.93E+08	0.0437101	0.18	not corr.	0.0439271	-5.3	0.18	subhedral
32	TK4	2.04E+08	0.0436394	0.17	not corr.	0.0438560	-7.0	0.17	subhedral
33	TK4	2.04E+08	0.0433056	0.30	not corr.	0.0435205	-14.5	0.30	subhedral
34	TK4	2.32E+08	0.0464998	1.25	not corr.	0.0467306	58.1	1.25	subhedral
35	TK4	2.42E+08	0.0456136	0.21	not corr.	0.0458399	38.0	0.21	subhedral
36	TK4	2.20E+08	0.0457574	0.23	not corr.	0.0459845	41.2	0.23	framboidal
37	TK4	2.08E+08	0.0439787	0.26	not corr.	0.0441969	0.8	0.26	subhedral
38	TK4	2.02E+08	0.0439221	0.35	not corr.	0.0441401	-0.5	0.35	subhedral
39	TK4	2.00E+08	0.0440006	0.44	not corr.	0.0442190	1.3	0.44	subhedral
40	TK6	2.52E+08	0.0451922	0.14	not corr.	0.0454165	28.4	0.14	subhedral
41	TK6	1.31E+08	0.0446530	0.55	not corr.	0.0448746	16.1	0.55	framboidal
42	TK6	2.64E+08	0.0474210	0.35	not corr.	0.0476564	79.1	0.35	subhedral
43	TK6	2.60E+08	0.0440718	0.69	not corr.	0.0442906	2.9	0.69	framboidal
44	TK6	2.52E+08	0.0460562	0.62	not corr.	0.0462848	48.0	0.62	subhedral
45	TK6	2.26E+08	0.0452224	0.74	not corr.	0.0454469	29.1	0.74	subhedral
46	TK6	2.42E+08	0.0441792	0.47	not corr.	0.0443985	5.3	0.47	subhedral
47	TK6	2.53E+08	0.0460601	0.61	not corr.	0.0462887	48.1	0.61	framboidal
48	TK6	2.37E+08	0.0451635	0.65	not corr.	0.0453876	27.7	0.65	framboidal
49	TK6	2.15E+08	0.0480089	3.90	not corr.	0.0482472	92.5	3.90	framboidal
50	TK6	2.10E+08	0.0449094	0.62	not corr.	0.0451323	21.9	0.62	subhedral
51	TK6	2.32E+08	0.0452683	0.35	not corr.	0.0454930	30.1	0.35	framboidal
52	TK6	1.45E+08	0.0461498	1.41	not corr.	0.0463788	50.2	1.41	framboidal
53	TK6	1.89E+08	0.0451321	0.13	not corr.	0.0453561	27.0	0.13	subhedral

Fluid inclusion measurements

Fluid inclusions hosted in carbonate, spatially associated with selenide minerals, were investigated by Raman spectroscopy and microthermometry at Georg-August Universität Göttingen (Germany). Sample TK1 is from the so-called barite vein, sample TK7 is from the mid-sole to the south. Firstly, the host carbonate was carbon-coated for cathodoluminescence imaging. A hot-cathode microscope HC3-LM, Simon-Neuser, with a camera Infinity 5 Teledyne Lumenera attached to a microscope Olympus BH-2, was used for distinguishing types and generations of carbonate. Subsequently, carbonate-hosted fluid inclusions had their phase-transition temperatures measured with a Linkam THMS G-600 heating-freezing stage (**ESMA-Table S3**). The stage, cooled with liquid nitrogen, was calibrated with a set of synthetic fluid-inclusion standards. For temperatures around -56.6 °C – i.e., the melting point of CO_2 – and around 0 °C, the accuracy is better than 0.5 °C, whereas for temperatures between room temperature and 200 °C the accuracy is better than 2 °C. Pressure-temperature conditions of fluid trapping were calculated using the ISOC software[60].

ESMA-Table S3. Results of microthermometric measurements of fluid inclusions in carbonate. Abbreviations: L – liquid; V – vapor; T_e – eutectic melting temperature; $T_{m\ HH}$ – hydrohalite melting temperature; $T_{m\ ice}$ – ice melting temperature; $T_{m\ clathr}$ – dissociation temperature of gas-clathrate hydrates; $T_{h\ total}$ – total homogenization temperature.

Sample	No.	Phases Tr	Vol% H_2O	Size [μm]	T_e	$T_{m\ HH}$	$T_{m\ ice}$	$T_{m\ clathr}$	$T_{h\ total}$	L/V	Composition	Type	Wt% H_2O	Wt% NaCl	Wt% $CaCl_2$	Total salinity
TK1c	1	LV	0.95	4 × 17	-53.0	-24.9	-23.6		162	L	H_2O + NaCl +$CaCl_2$	primary	76	14	10	24
TK1c	2	LV	0.95	1 × 4		-29.3	-25.7		159	L	H_2O + NaCl +$CaCl_2$	primary	76	8	16	24
TK1c	3	LV	0.95	3 × 4		-24.8	-23.7		147	L	H_2O + NaCl +$CaCl_2$	primary	76	14	10	24
TK1c	4	LV	0.95	2 × 6		-36.4	-24.2		126	L	H_2O + NaCl +$CaCl_2$	primary	77	3	19	23
TK1c	5	LV	0.95	3 × 6		-27.2	-21.5		136	L	H_2O + NaCl +$CaCl_2$	primary	78	10	13	22
TK1c	6	LV	0.95	2 × 2							H_2O + NaCl +$CaCl_2$	primary				
TK1c	7	LV	0.95	0 × 6	-53.0	-25.5	-20.0		152	L	H_2O + NaCl +$CaCl_2$	primary	78	12	10	22
TK1c	8	LV	0.95	3 × 7		-30.0	-18.6		174	L	H_2O + NaCl +$CaCl_2$	primary	80	6	14	20
TK1c	9	LV	0.95	7 × 7		-24.1	-18.8		152	L	H_2O + NaCl +$CaCl_2$	primary	79	14	7	21
TK1c	10	LV	0.95	3 × 7					120	L	H_2O + NaCl +$CaCl_2$	primary				
TK1c	11	LV	0.95	3 × 3					134	L	H_2O + NaCl +$CaCl_2$	primary				
TK1c	12	LV	0.95	4 × 4	-52.0	-32.9	-31.6		129	L	H_2O + NaCl +$CaCl_2$	primary	73	6	21	27
TK1c	13	LV	0.97	1 × 6							H_2O + NaCl +$CaCl_2$	primary				
TK1c	14	LV	0.90	6 × 15		-32.9	-30.0	-6.4	159	L	H_2O + NaCl +$CaCl_2$	primary	74	6	20	26
TK1c	15	LV	0.97	1 × 6					89	L	H_2O + NaCl +$CaCl_2$	primary				
TK1c	16	LV	0.95	4 × 4		-30.0	-19.8		169	L	H_2O + NaCl +$CaCl_2$	primary	79	6	15	21
TK1c	17	LV	0.95	3 × 6	-51.0	-30.0	-23.9		171	L	H_2O + NaCl +$CaCl_2$	primary	77	7	16	23
TK1c	18	LV	0.95	4 × 7							H_2O + NaCl +$CaCl_2$	primary				
TK1c	19	LV	0.95	1 × 4					139	L	H_2O + NaCl +$CaCl_2$	primary				
TK1c	20	LV	0.95	2 × 3					129	L	H_2O + NaCl +$CaCl_2$	primary				
TK1c	21	LV	0.95	2 × 3					148	L	H_2O + NaCl +$CaCl_2$	primary				
TK1c	22	LV	0.95	1 × 7					174	L	H_2O + NaCl +$CaCl_2$	primary				

ESMA-Table S3. (continued).

Sample	No.	Phases Tr	Vol% H_2O	Size [µm]	T_e	$T_{m\ HH}$	$T_{m\ ice}$	$T_{m\ clathr}$	$T_{h\ total}$	L/V	Composition	Type	Wt% H_2O	Wt% NaCl	Wt% $CaCl_2$	Total salinity
TK1b	1	LV	0.95	3 × 9	-52.0	-30.1	-18.1		140	L	H_2O + NaCl +$CaCl_2$	primary	80	6	14	20
TK1b	2	LV	0.95	1 × 4					141	L	H_2O + NaCl +$CaCl_2$	primary				
TK1b	3	LV	0.95	3 × 3					152	L	H_2O + NaCl +$CaCl_2$	primary				
TK1b	4	LV	0.95	3 × 4					140	L	H_2O + NaCl +$CaCl_2$	primary				
TK1b	5	LV	0.95	3 × 4							H_2O + NaCl +$CaCl_2$	primary				
TK1b	6	LV	0.95	3 × 6					141	L	H_2O + NaCl +$CaCl_2$	primary				
TK1b	7	LV	0.95	2 × 4					139	L	H_2O + NaCl +$CaCl_2$	primary				
TK1b	8	LV	0.95	2 × 4		-26.8	-20.8		156	L	H_2O + NaCl +$CaCl_2$	primary	78	10	12	22
TK1b	9	LV	0.95	3 × 4		-29.7	-21.7		139	L	H_2O + NaCl +$CaCl_2$	primary	78	7	15	22
TK1b	10	LV	0.95	3 × 4					154	L	H_2O + NaCl +$CaCl_2$	primary				
TK1b	11	LV	0.95	3 × 4					158	L	H_2O + NaCl +$CaCl_2$	primary				
TK1b	12	LV	0.95	3 × 6		-24.7	-16.9		157	L	H_2O + NaCl +$CaCl_2$	primary	80	12	8	20
TK7b	1	LV	0.97	2 × 3			-22.2		154	L	H_2O + NaCl +$CaCl_2$	primary				24
TK7b	2	LV	0.95	2 × 6			-23.1		152	L	H_2O + NaCl +$CaCl_2$	primary				24
TK7b	3	LV	0.95	2 × 4			-22.7				H_2O + NaCl +$CaCl_2$	primary				24
TK7b	4	LV	0.97	3 × 4							H_2O + NaCl +$CaCl_2$	primary				
TK7b	5	LV	0.97	2 × 2							H_2O + NaCl +$CaCl_2$	primary				
TK7b	6	LV	0.95	3 × 4							H_2O + NaCl +$CaCl_2$	primary				
TK7b	7	LV	0.97	2 × 2							H_2O + NaCl +$CaCl_2$	primary				
TK7b	8	LV	0.90	1 × 7			-15.8		159	L	H_2O + NaCl +$CaCl_2$	primary				19
TK7b	9	LV	0.95	2 × 3					146	L	H_2O + NaCl +$CaCl_2$	primary				

U–Pb dating by LA–ICP–MS

Reddish calcite from an oxidized domain of a carbonate–hematite vein (Fig. S1) was analyzed in situ for U–Th–Pb isotopes by laser ablation – inductively coupled plasma – mass spectrometry (LA–ICP–MS) at Karlsruhe Institute of Technology, Germany. The measurements were performed on a polished thick section, using a 193-nm ArF Excimer laser (Teledyne Photon Machines, Analyte Excite+), coupled to a Thermo-Scientific Element XR instrument. The carbonate gangue of unknown age was dated together with reference material NIST612 soda-lime glass, WC-1 calcite[61], B6 calcite (in-house), Duff–Brown calcite[62], and JT-1 calcite[63], using a laser spot diameter of 65 (for NIST612 glass) and 135 µm (for calcite), a laser fluence of 2.2 J/cm², a 10 Hz repetition rate, RF = 1210 W, and a mixed Ar-He-N_2 carrier gas consisting of Ar = 0.91 l/min, He (cup) = 0.20 l/min, He (cell) = 0.30 l/min, and N_2 = 10 ml. Measured masses were 206, 207, 208, 232, and 238 with dwell times of 6.4 ms (206), 7.5 ms (207), 3.0 ms (208), 2.0 ms (232), and 4.6 ms (238). Ten pulses of pre-ablation were performed prior to each analysis of 15 s duration on spot, following 15 s background measurement. All raw data were corrected offline for daily instrumental drift (using NIST612) and mass offset (using calcite WC-1) by means of an in-house MS Excel spreadsheet program. No common lead correction was applied (ESME). Results of reference material NIST612, WC-1, Duff, and JT-1 and unknows are shown in ESME, along with additional MetaData explaining instrument conditions and measurement strategies. Multiple measurements of reference carbonate WC-1, Duff, and JT-1 yielded regression line intercept ages at 254.5 ± 2.6/4.5 Ma (2σ; MSWD = 1.02; n = 11), 64.6 ± 2.9/3.0 Ma (2σ; MSWD = 1.45; n = 11), and 13.96 ± 1.99/1.60 Ma (2σ; MSWD = 2.29; n = 12), respectively, in agreement with published ages. The results were plotted with Isoplot 2.49[64]. The U-Pb analyses of 91 spots on calcite yielded a regression line with a lower intercept ^{206}Pb/^{238}age of 60.34 +5.95/-6.02 Ma (Fig. S2).

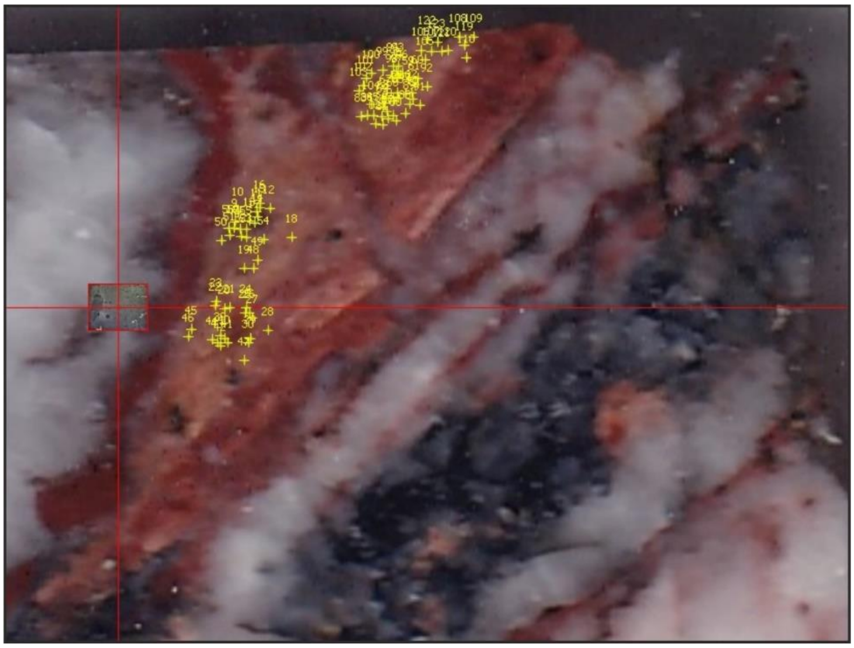

ESMA-Fig. S1. Laser spot positions on an oxidized calcite vein stained reddish by hematite.

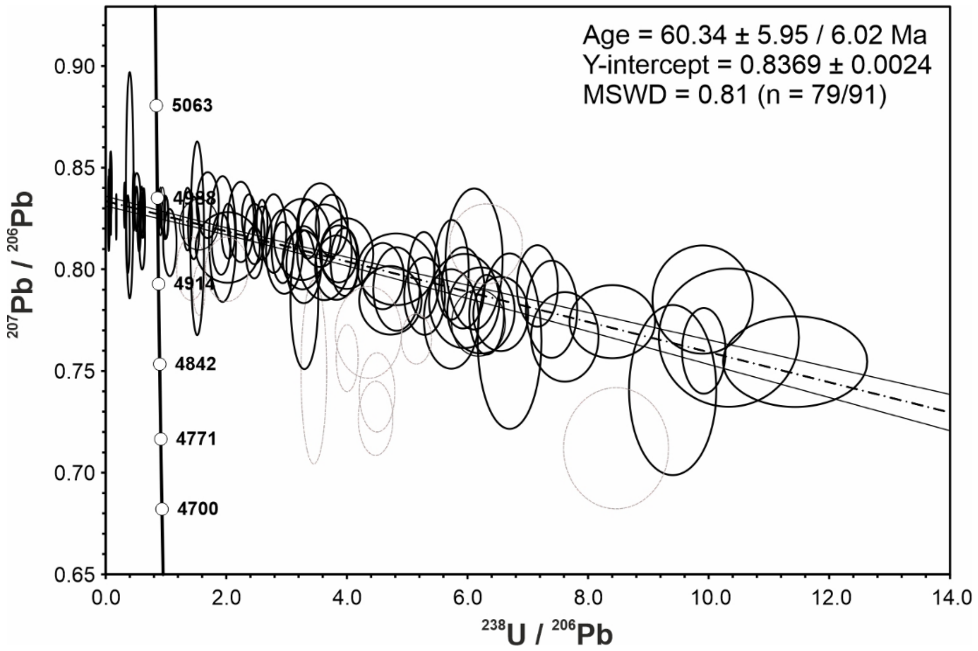

ESMA-Fig. S2. Results of in situ U–Pb dating of an oxidized calcite vein in Tera-Wasserburg plot.

References

47. Lohmeier, S., Cabral, A. R., Ehling, B.-C. & Zeh, A. Germanium and precious metals (Ag-Au-Pt-Pd) at low temperature: the hematite-carbonate-selenide vein system of Tilkerode, Harz Mountains, Germany. *Miner. Deposita* **58**, 1371–1379 (2023).

57. Ding, T. et al. Calibrated sulfur isotope abundance ratios of three IAEA sulfur isotope reference materials and V-CDT with a reassessment of the atomic weight of sulfur. *Geochim. Cosmochim. Acta* **65**, 2433–2437 (2001).

58. Crowe, D. E. & Vaughan, R. G. Characterization and use of isotopically homogeneous standards for in situ laser microprobe analysis of $^{34}S/^{32}S$ ratios. *Am. Mineral.* **81**, 187–193 (1996).

59. Whitehouse, M. J. Multiple sulfur isotope determination by SIMS: evaluation of reference sulfides for $\Delta^{33}S$ with observations and a case study on the determination of $\Delta^{36}S$. *Geostand. Geoanal. Res*. **37**, 19–33 (2013).

60. Bakker, R.J. Package FLUIDS 1. Computer programs for analysis of fluid inclusion data and for modelling bulk fluid properties. *Chem. Geol.* **194**, 1–3, 2–23; 10.1016/S0009-2541(02)00268-1 (2003).

61. Roberts, N.M.W. et al. A calcite reference material for LA-ICP-MS U-Pb geochronology. *Geochemistry, Geophysics, Geosystems* **18**, 2807–2814 (2017).

62. Wu, S. et al. In situ calcite U-Pb geochronology by high-sensitivity single-collector LA-SF-ICP-MS. *Science China Earth Sciences*, **65**, 1146–1160 (2022).

63. Guillong, M., Wotzlaw, J.-F., Looser, N. & Laurent, O. Evaluating the reliability of U-Pb laser ablation inductively coupled plasma mass spectrometry (LA-ICP-MS) carbonate geochronology: matrix issues and a potential calcite validation reference material. *Geochronology* **2**, 155–167 (2020).

64. Ludwig, K. Isoplot/Ex, rev. 2.49. A geochronological toolkit for microsoft excel. *Berkeley Geochronology Center, Spec. Publ*. **1a**, 1-55 (2001).
