# Supplementary figures and images for: Microbial life in deep-seated selenide veins reflected by extreme δ34S fractionation of framboidal pyrite
Source: Sci Rep. 2026 Jun 26;16:19627. doi: 10.1038/s41598-026-59857-1 (PMC13309615; doi:10.1038/s41598-026-59857-1)

Electronic Supplementary Material B – Documentation of analyzed SIMS spots

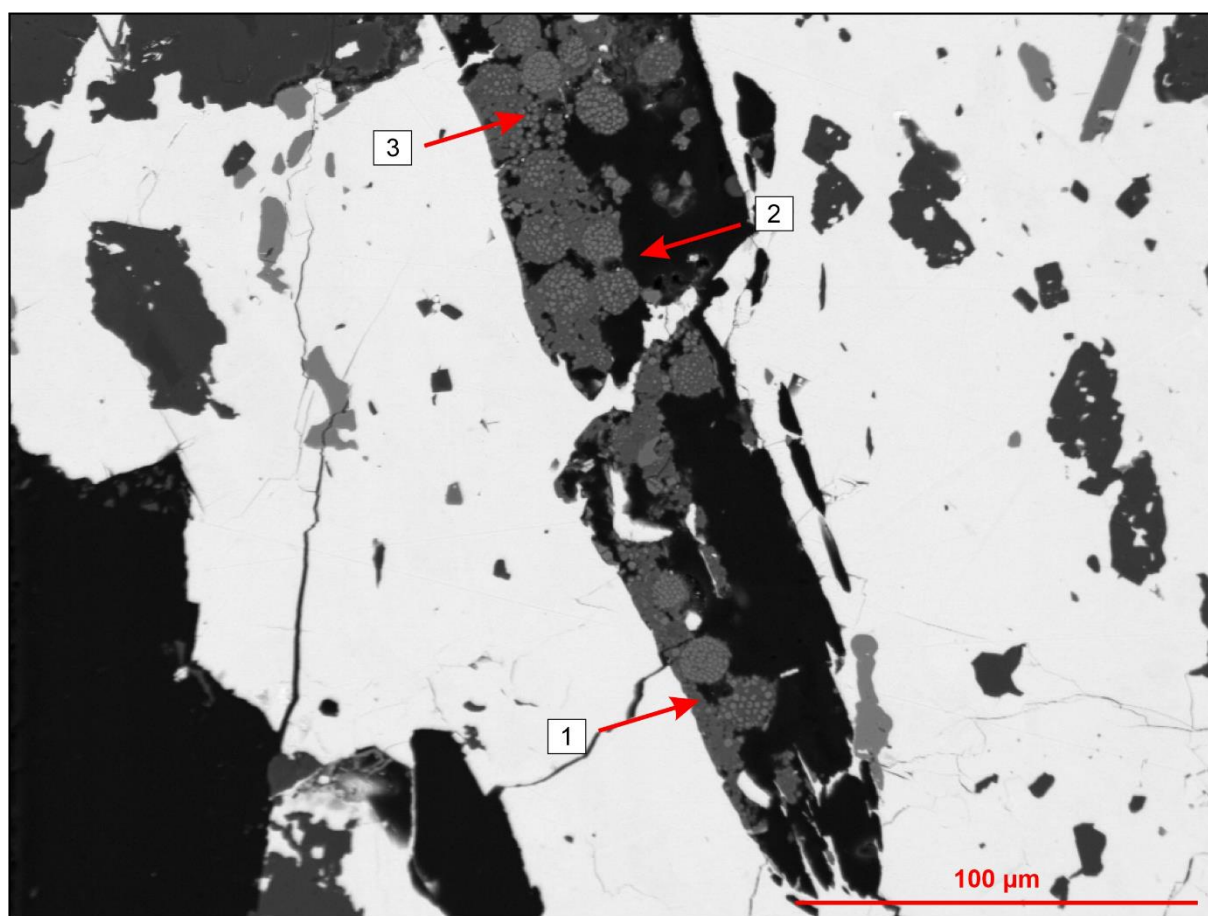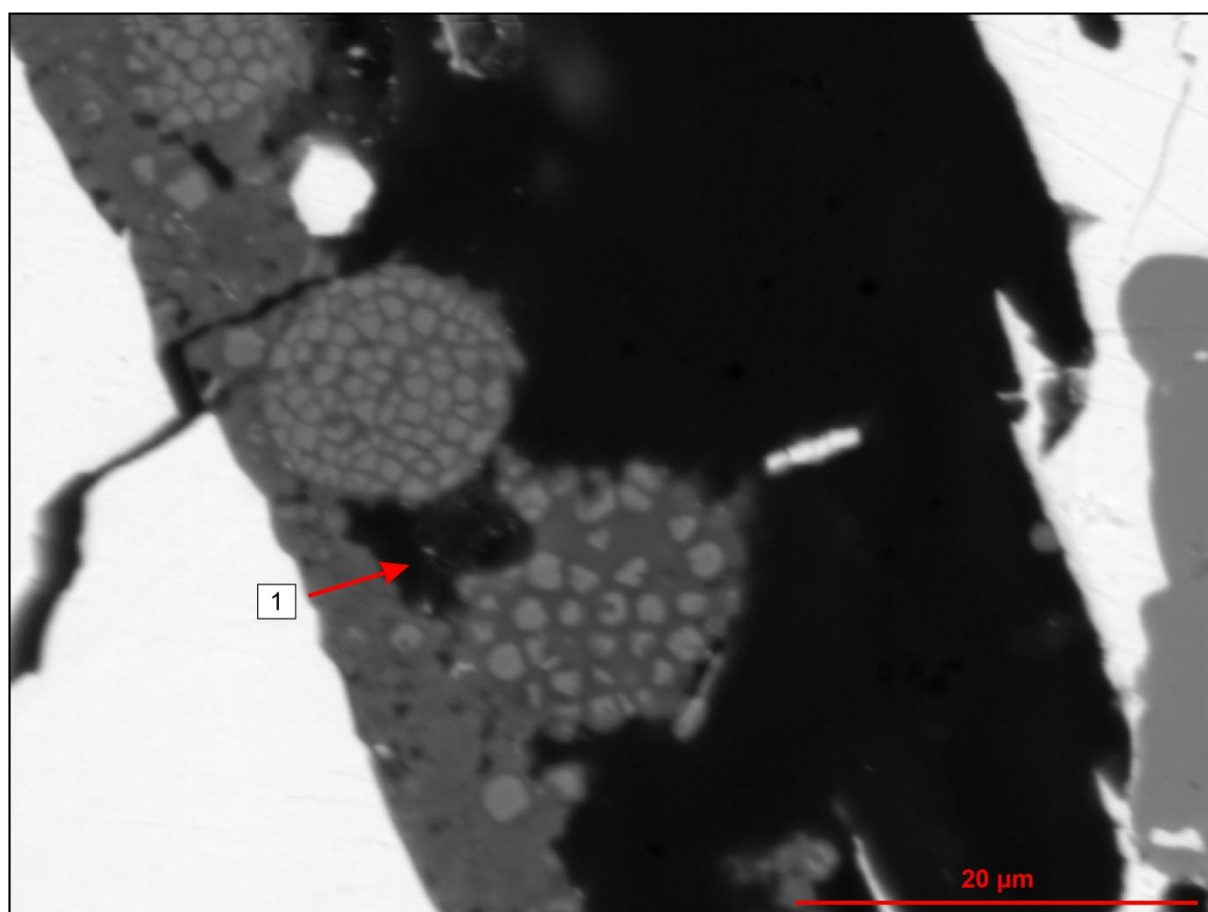

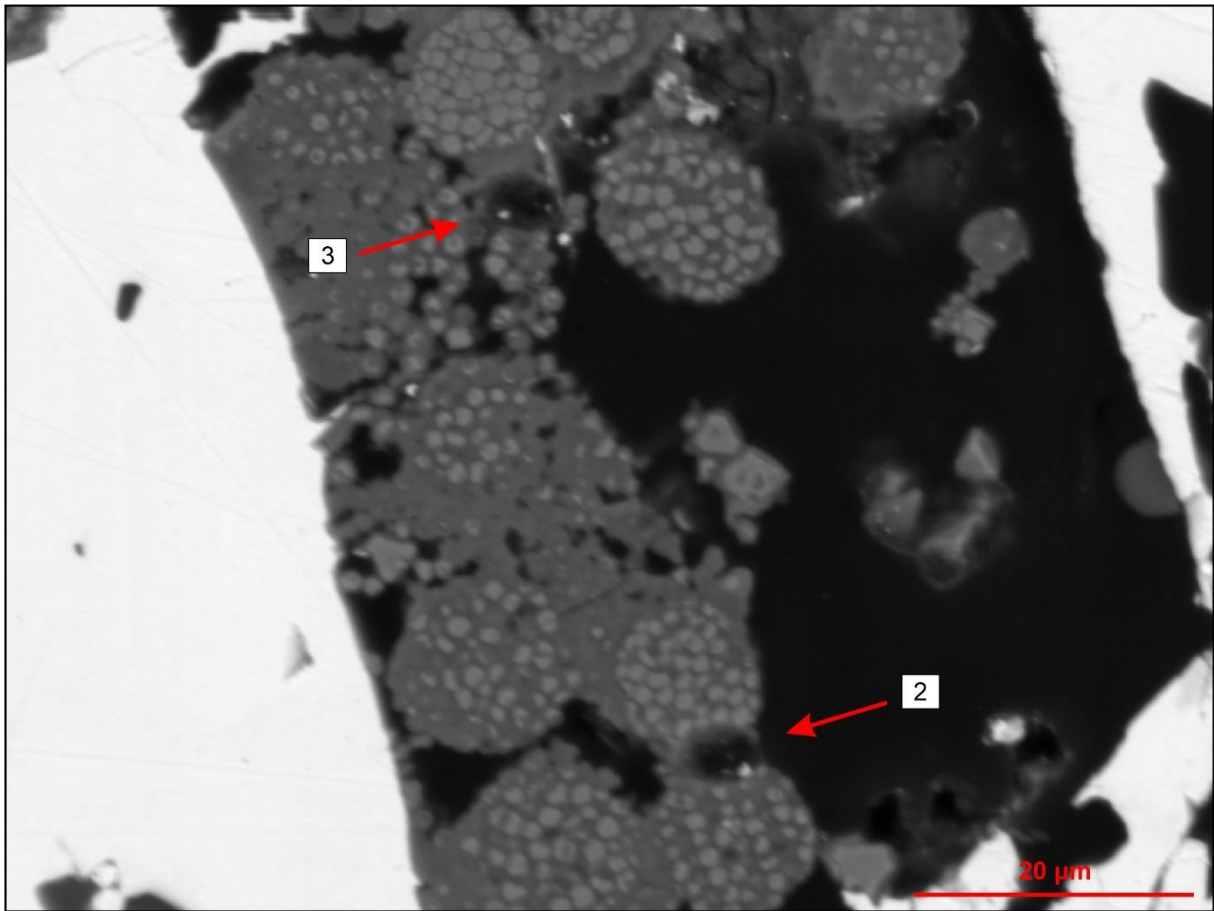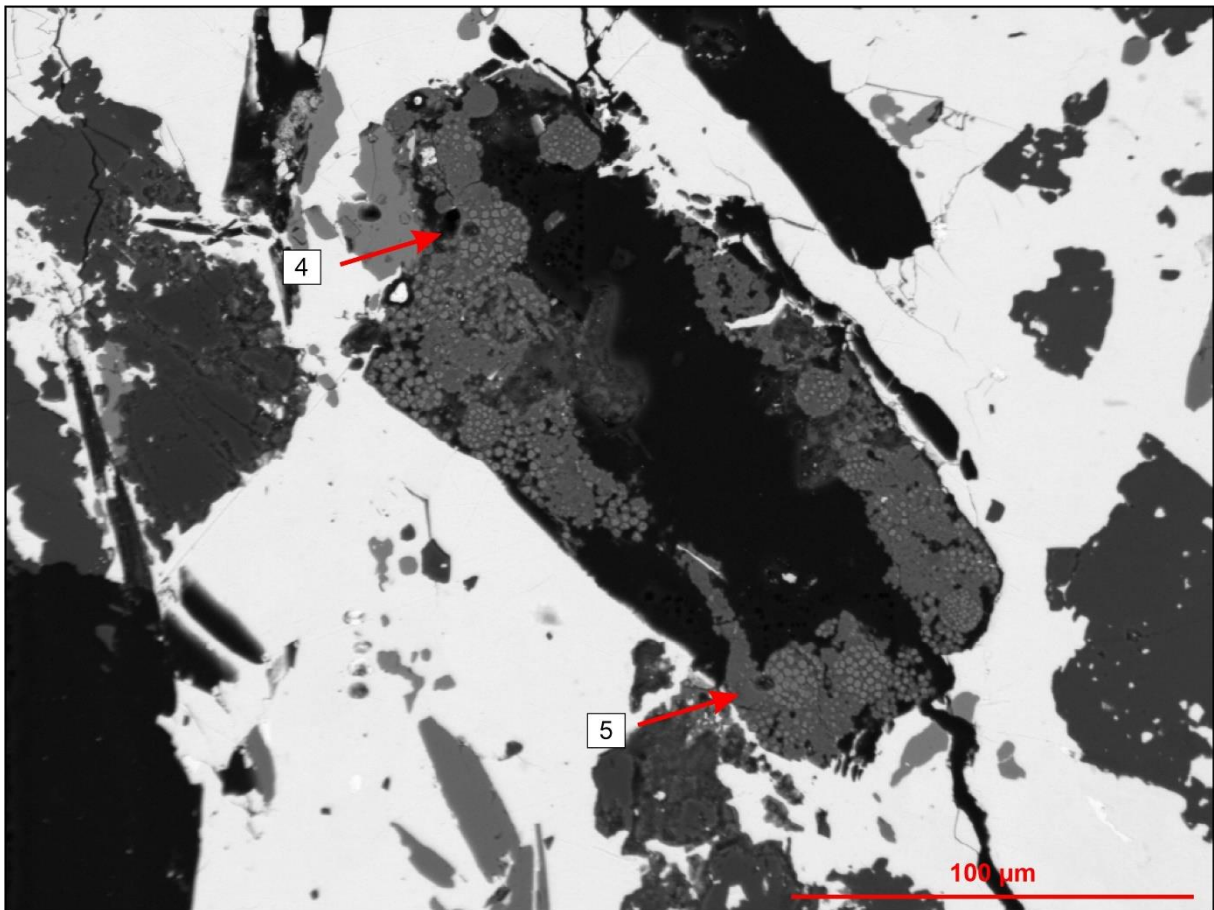

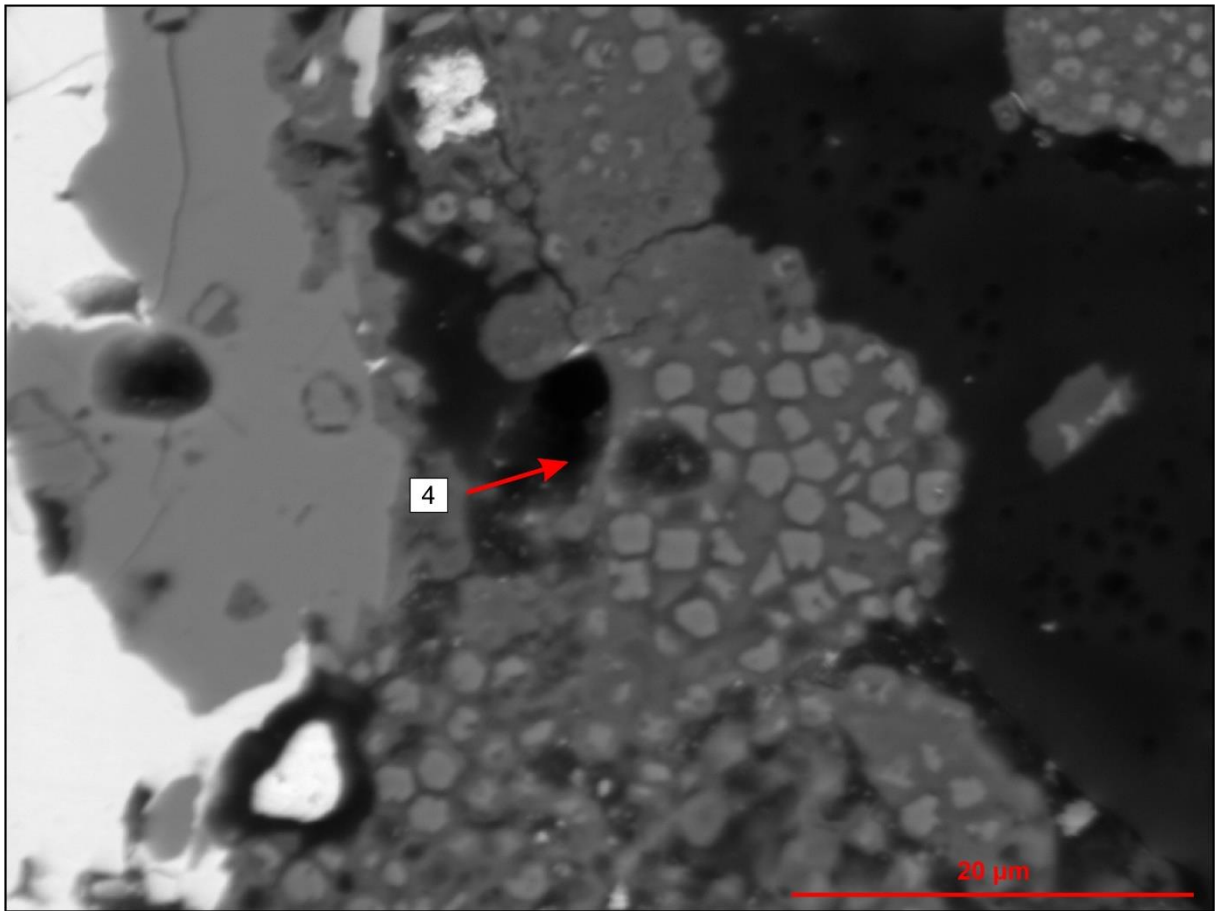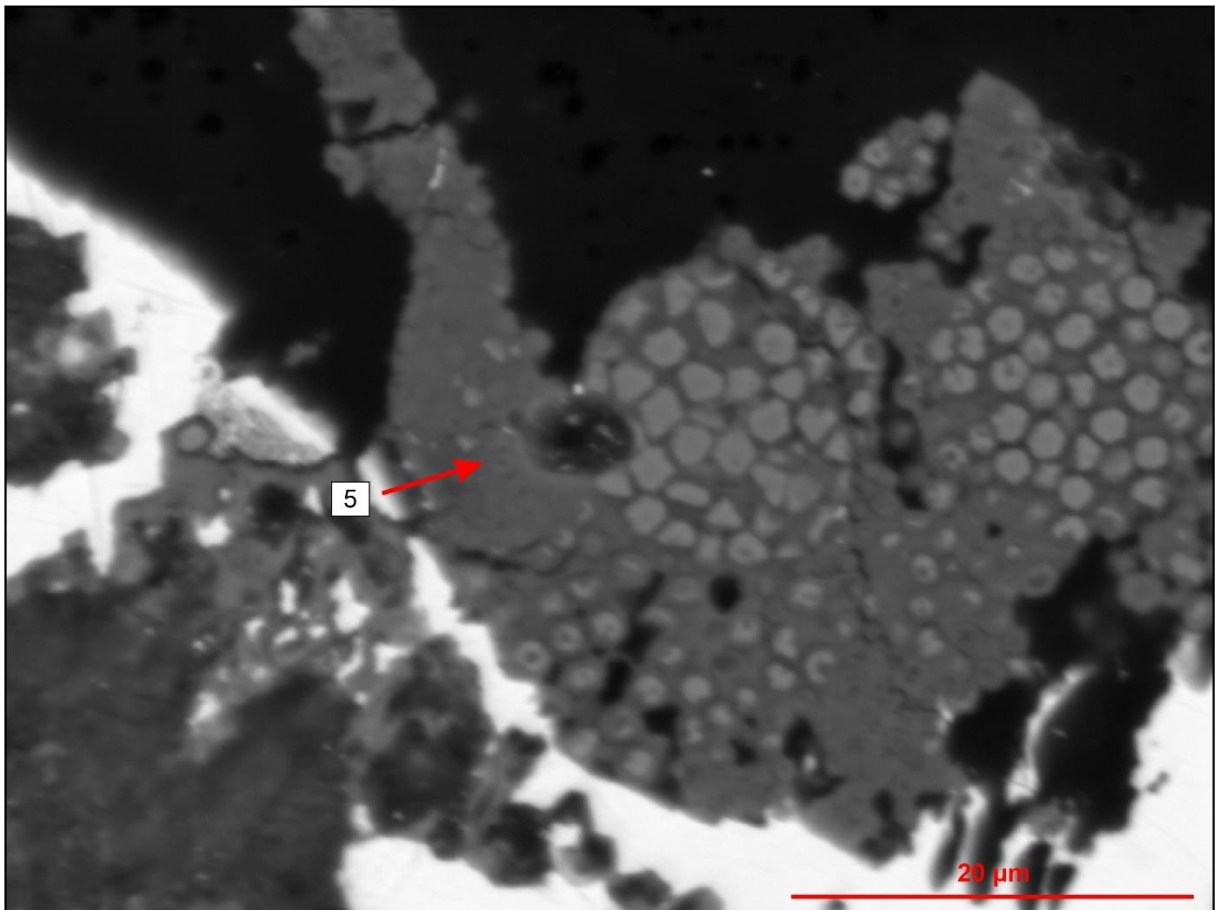

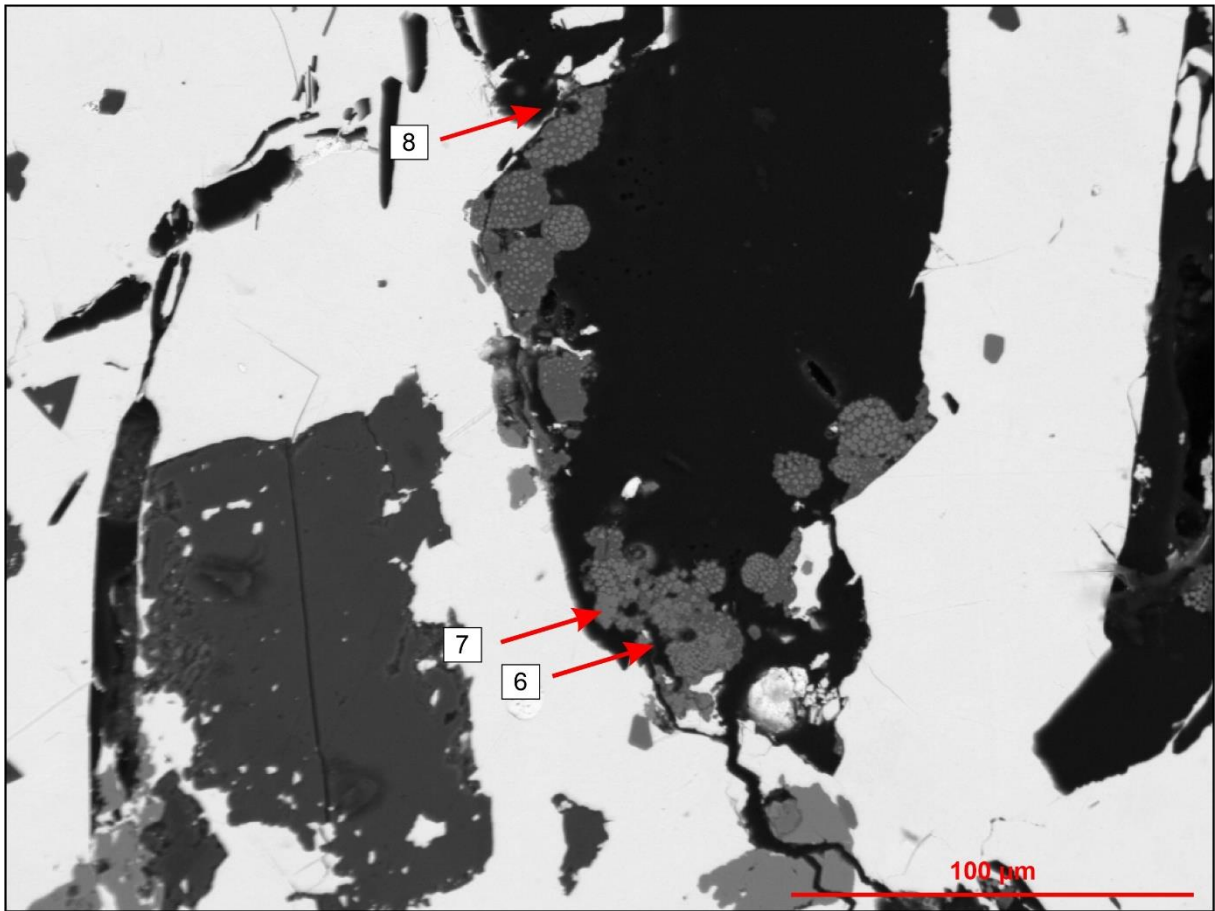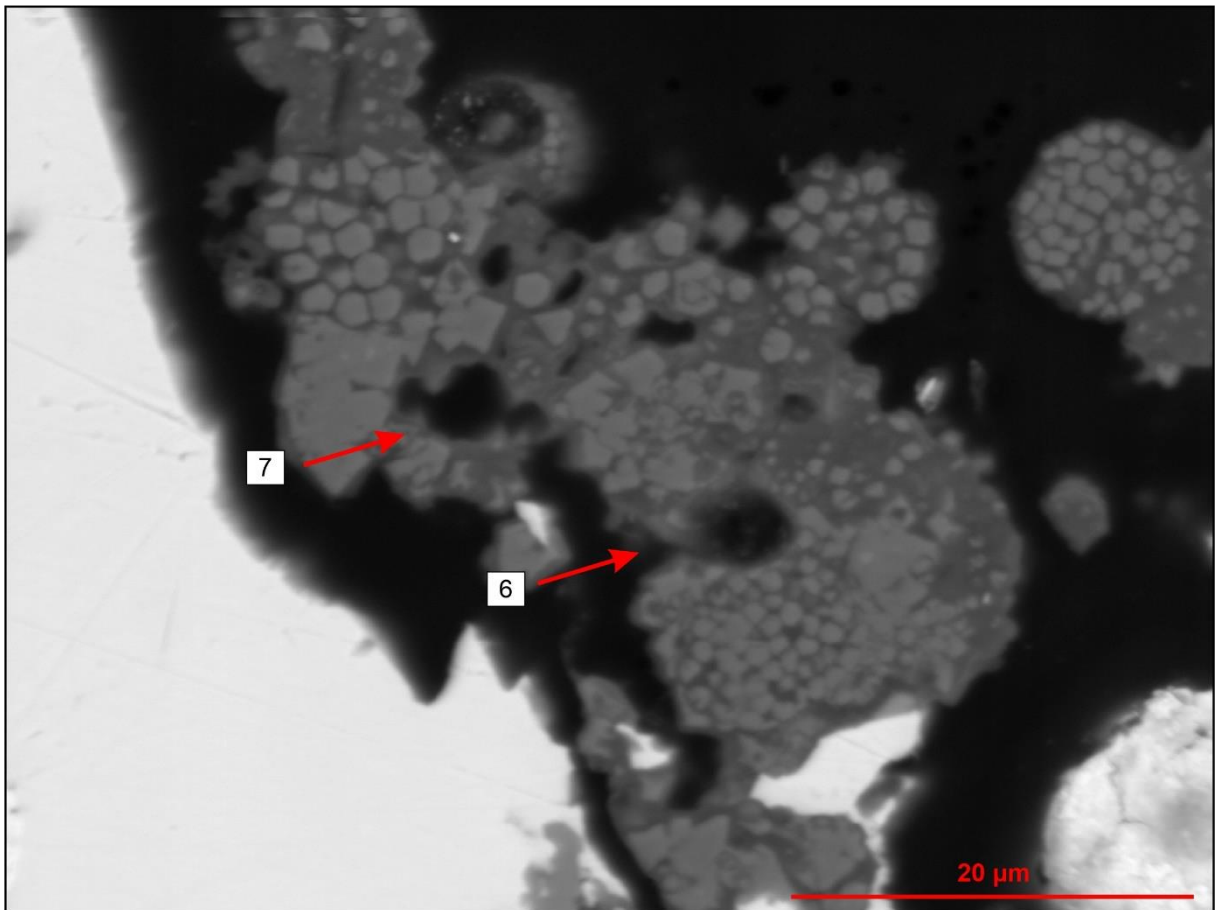

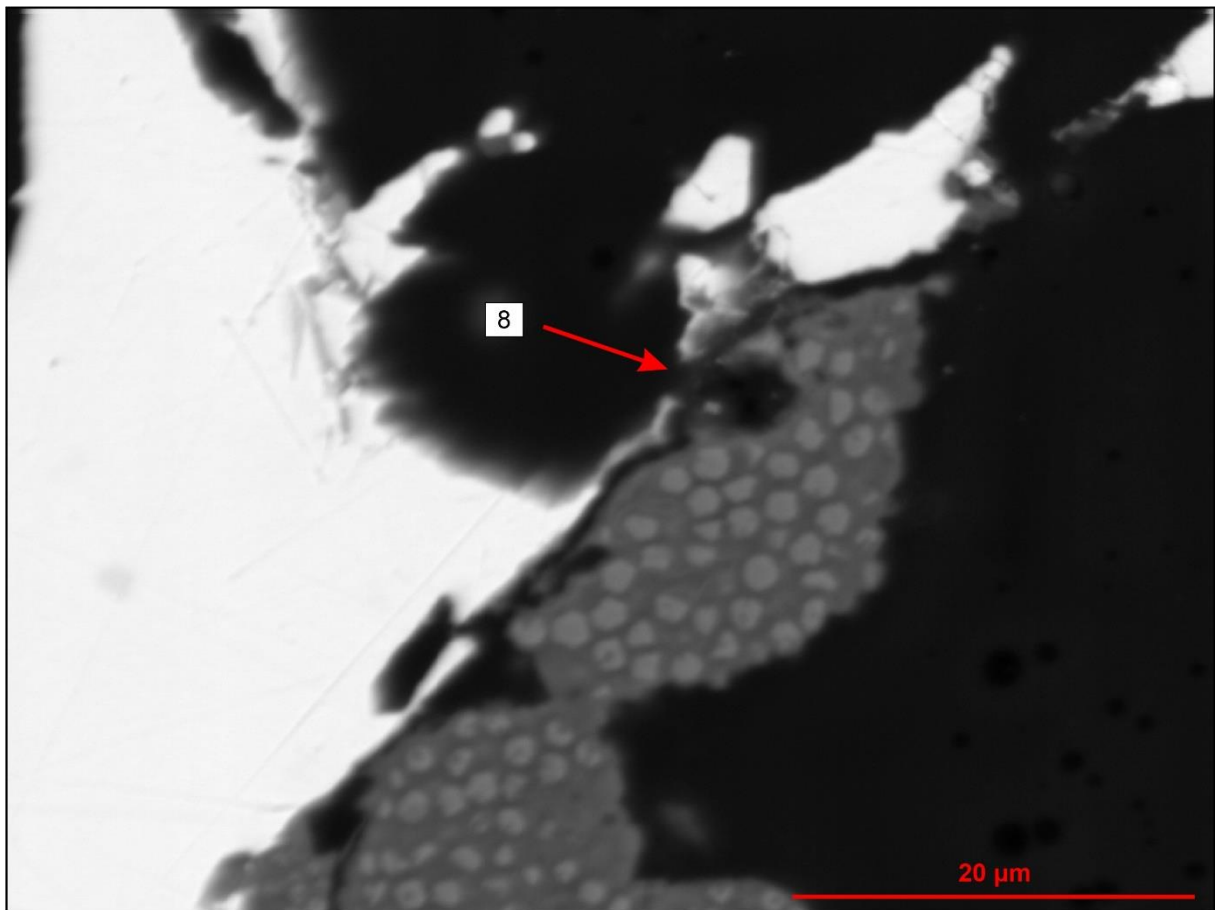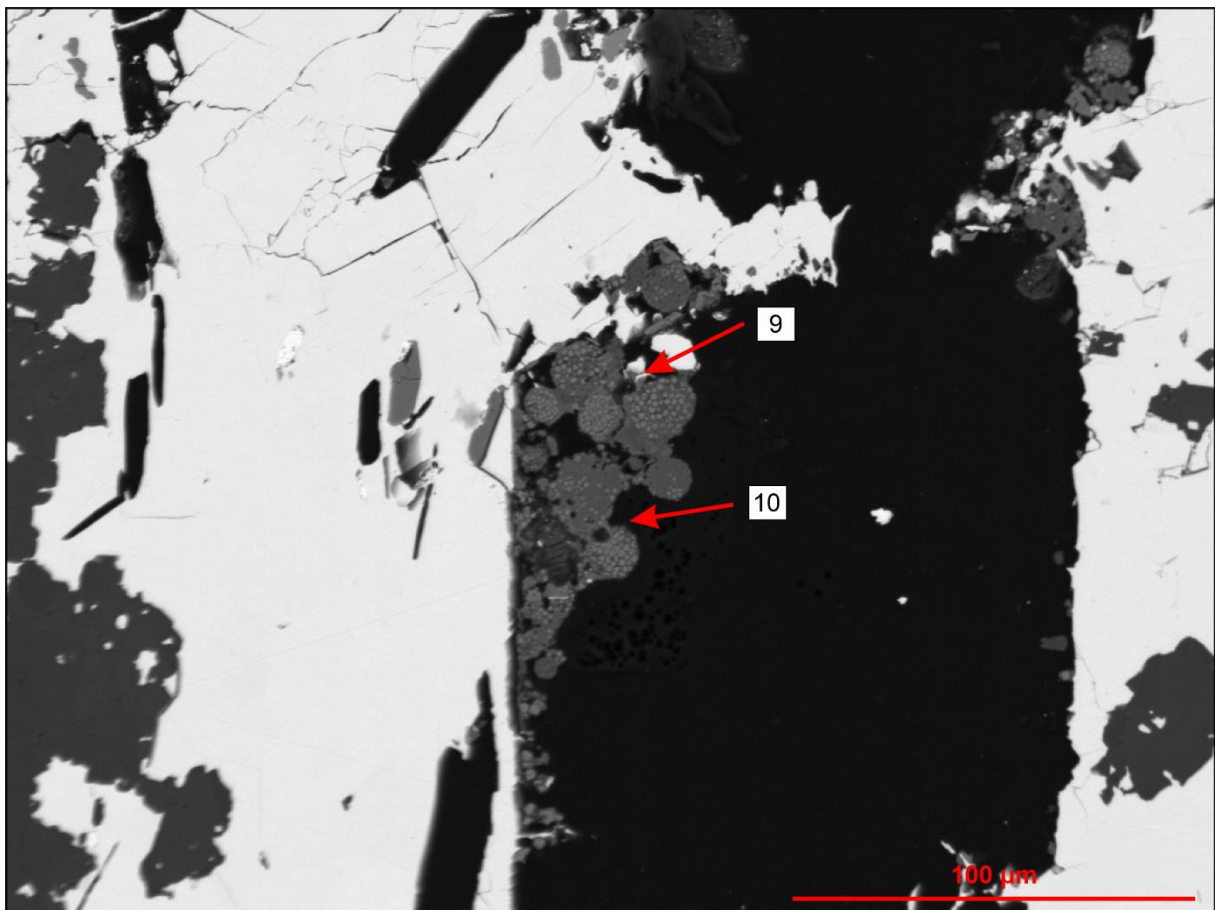

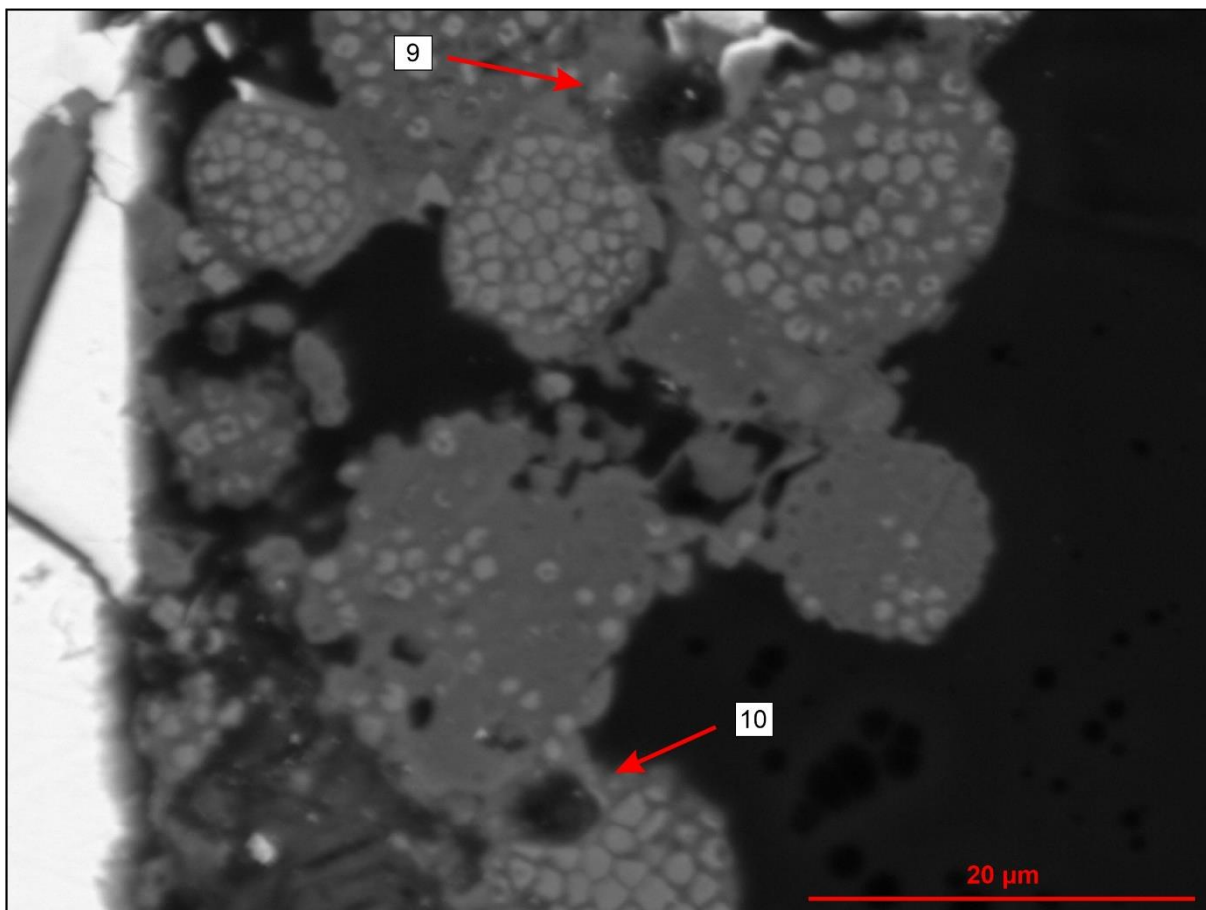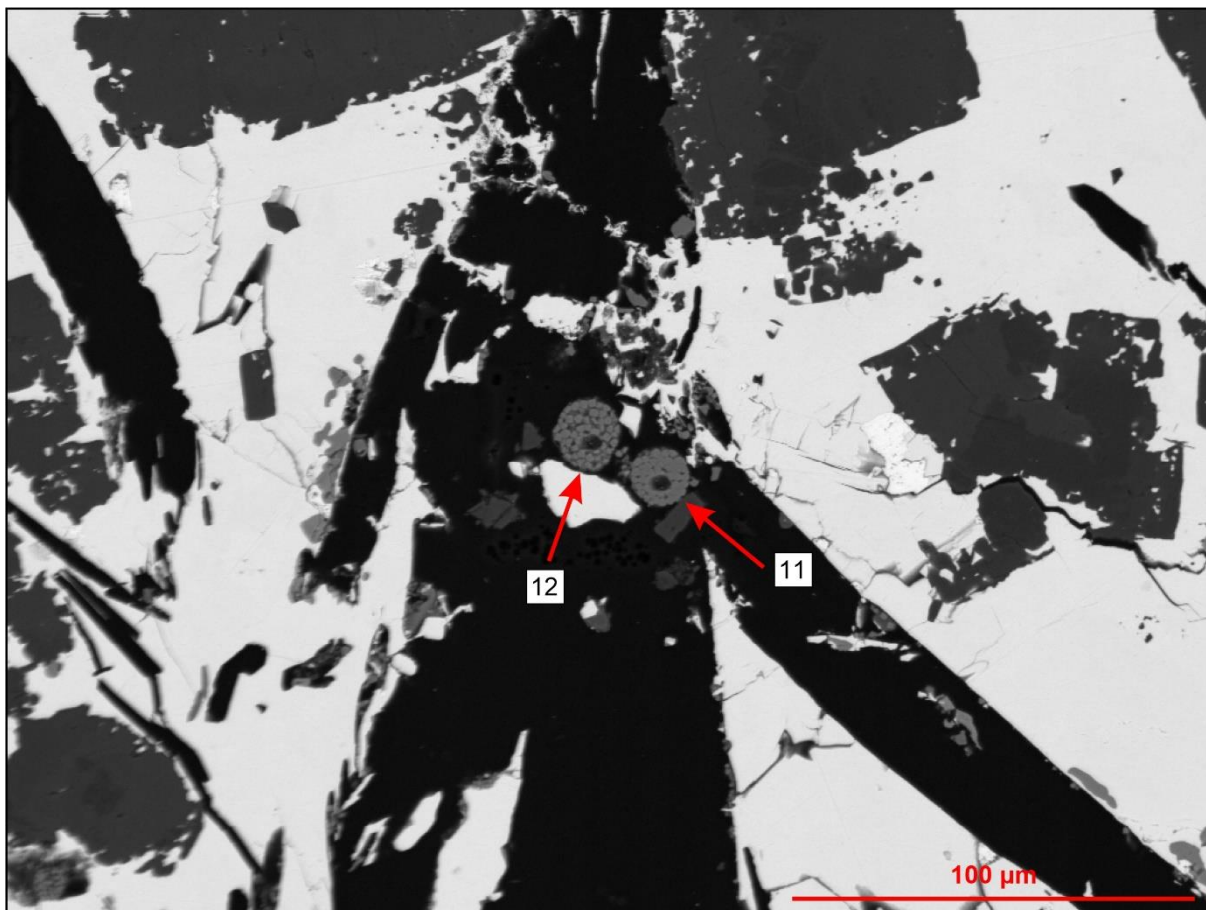

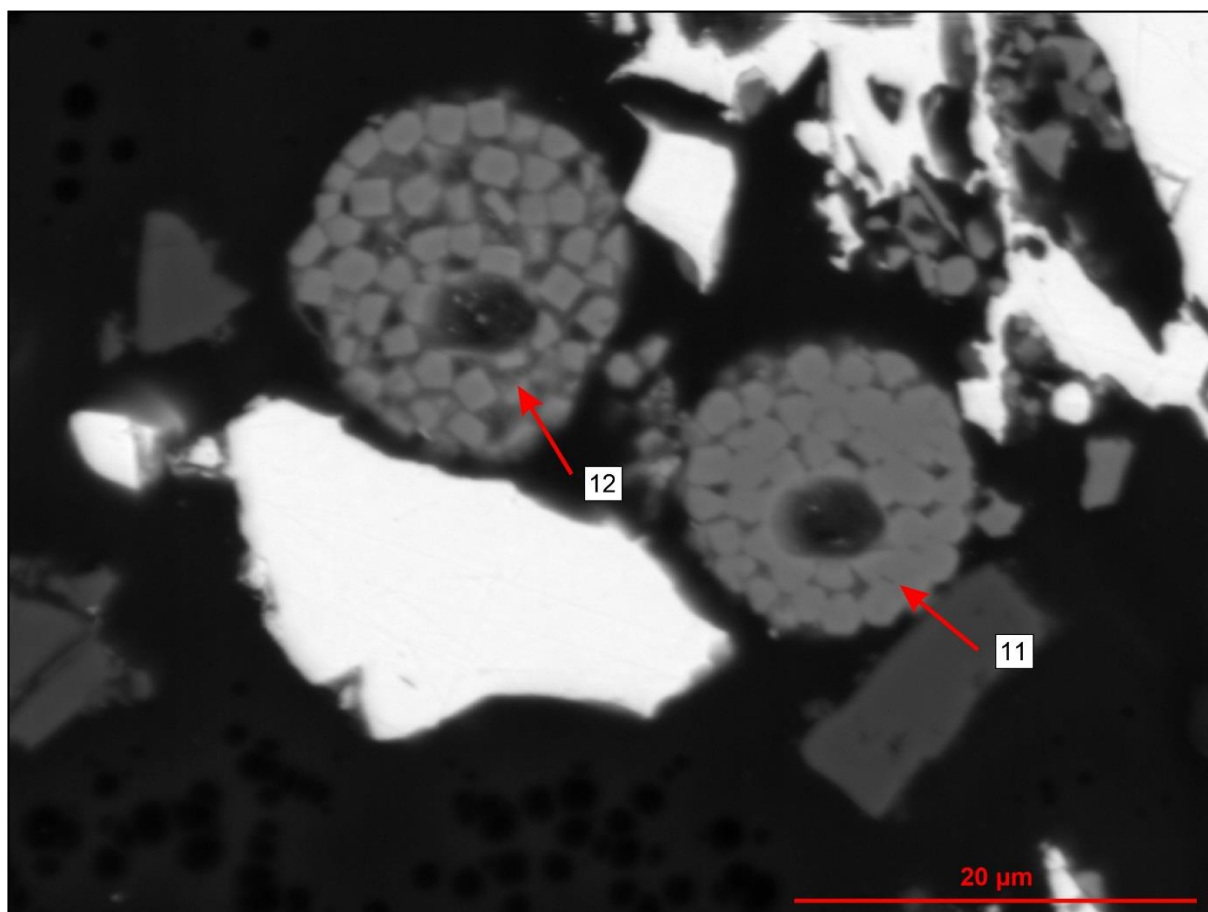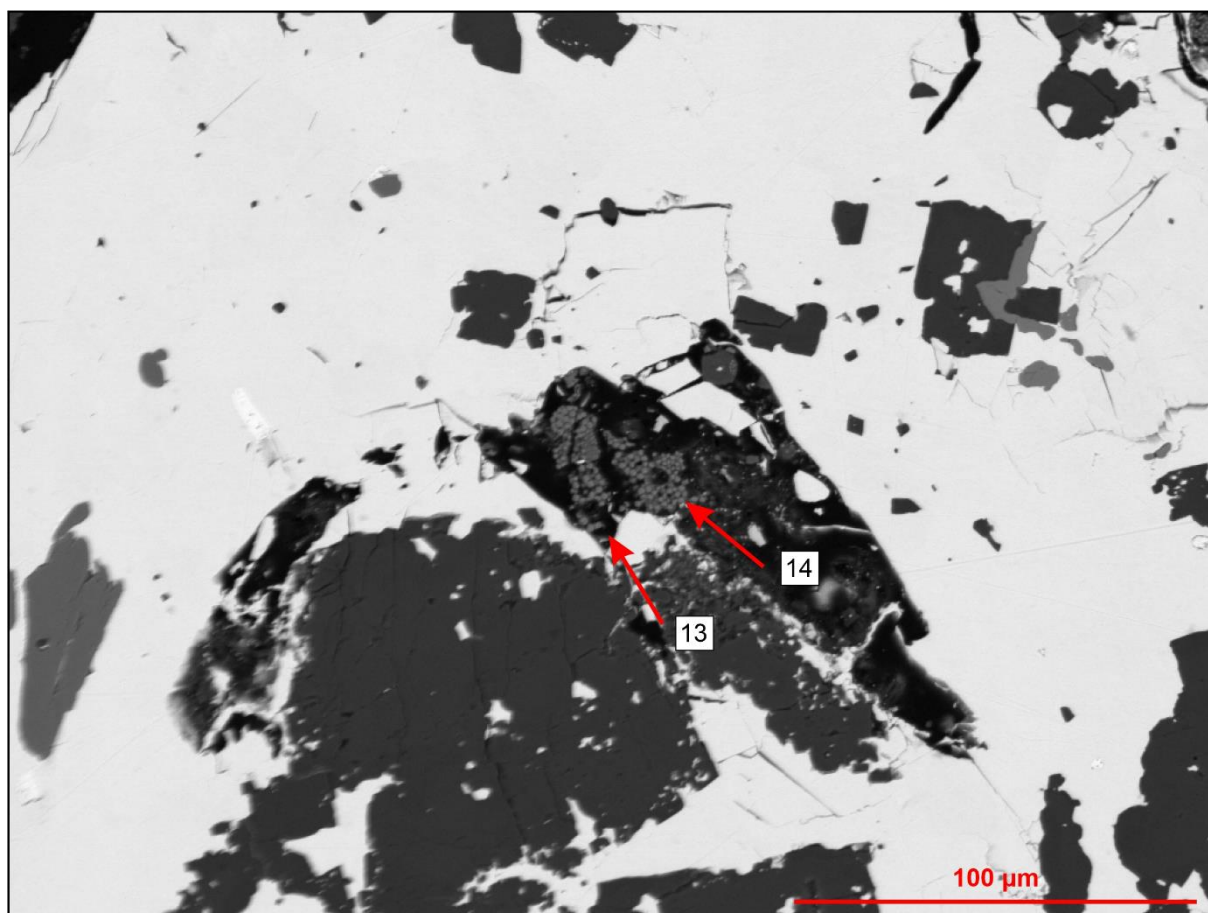

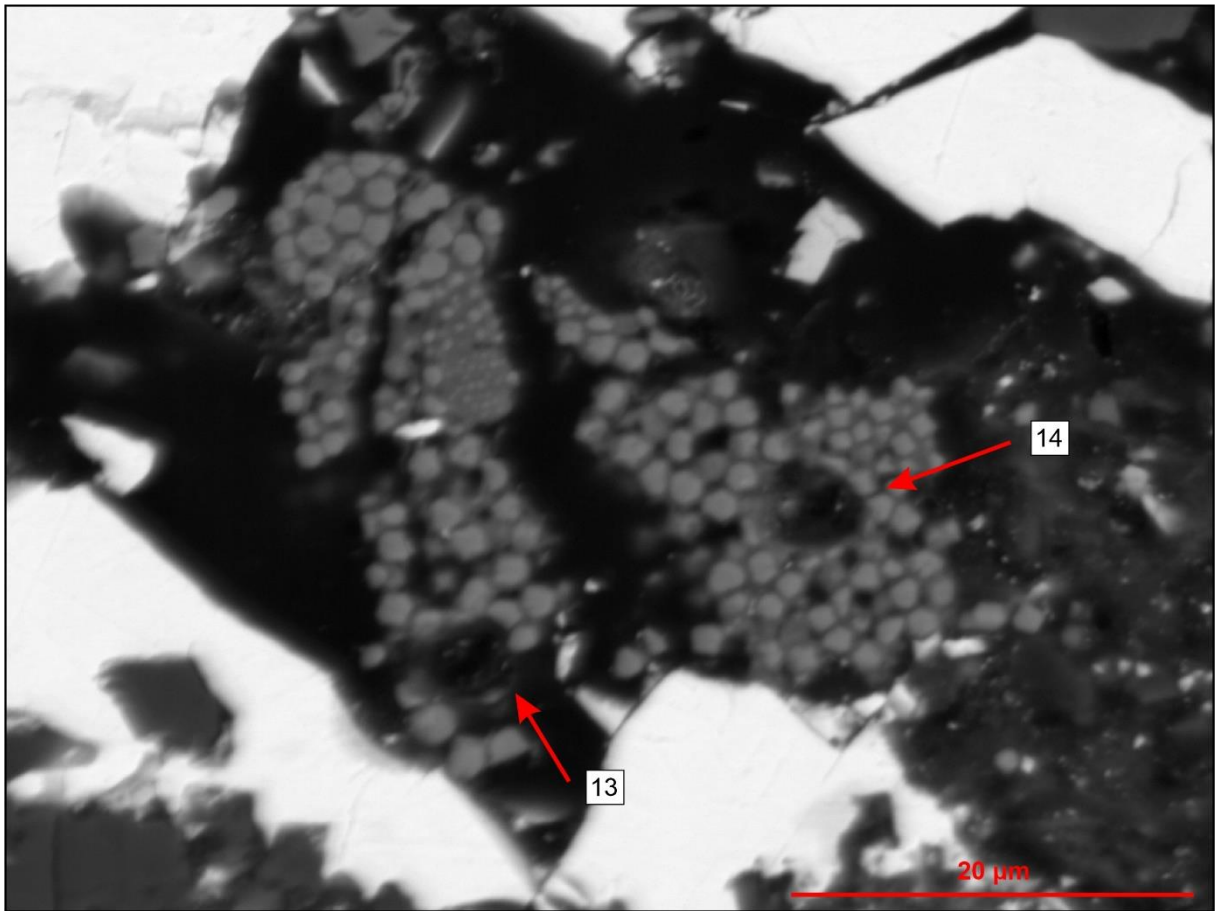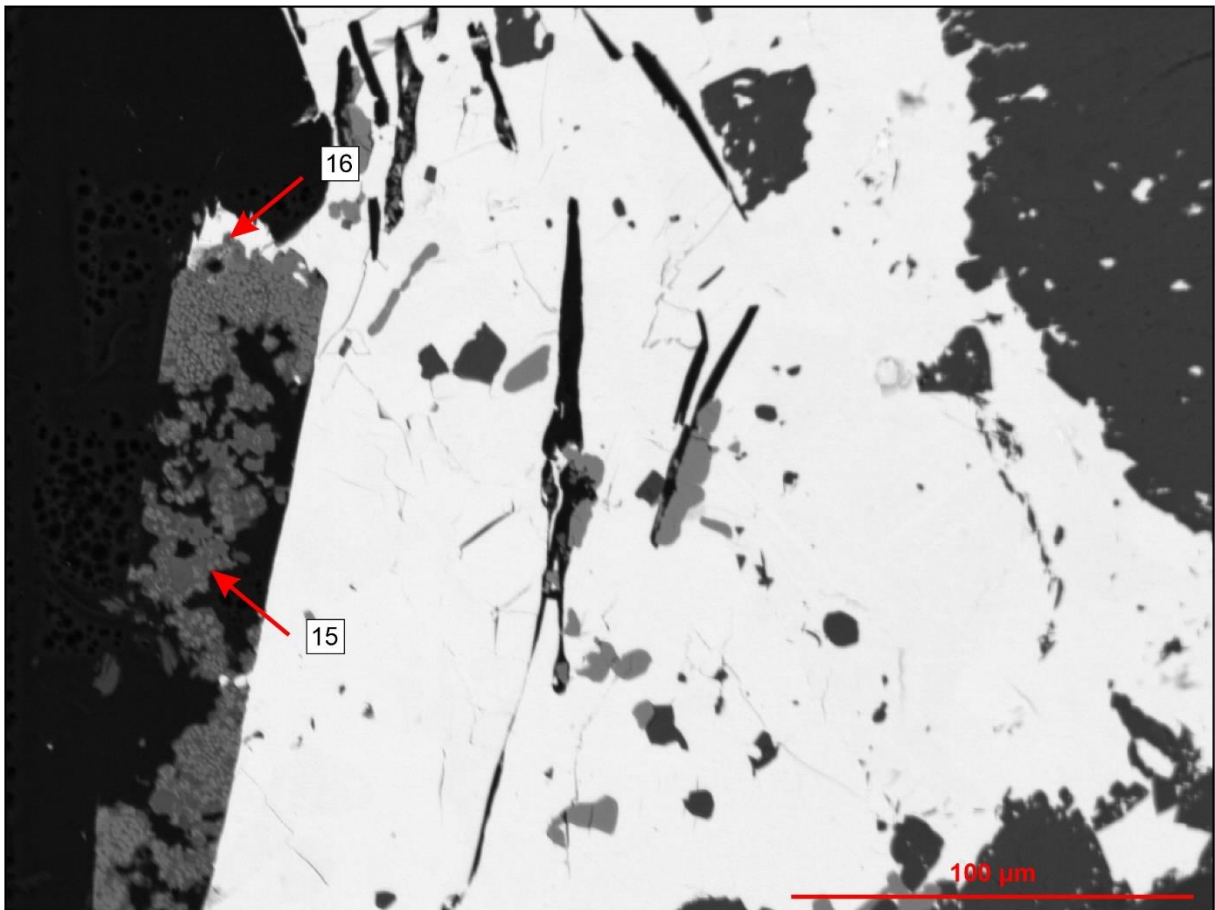

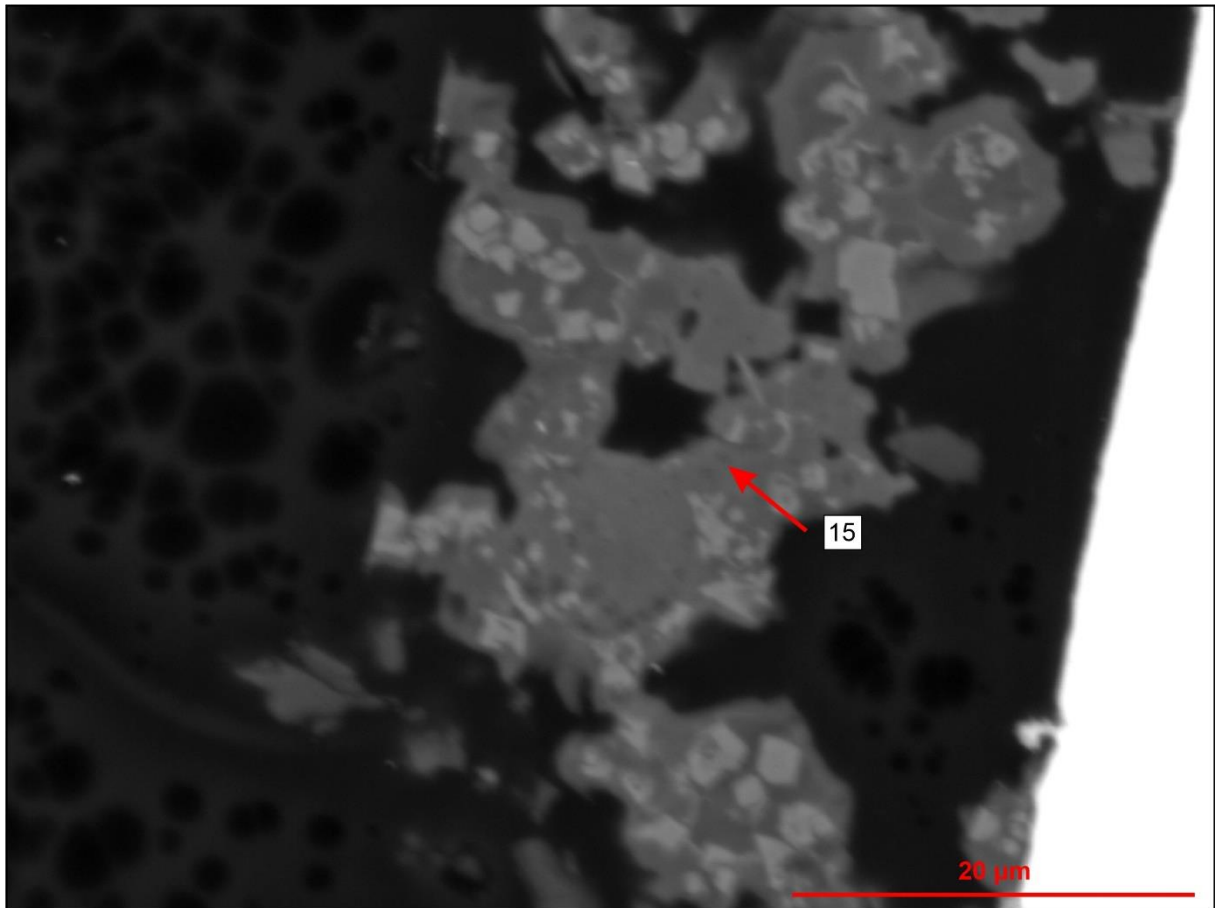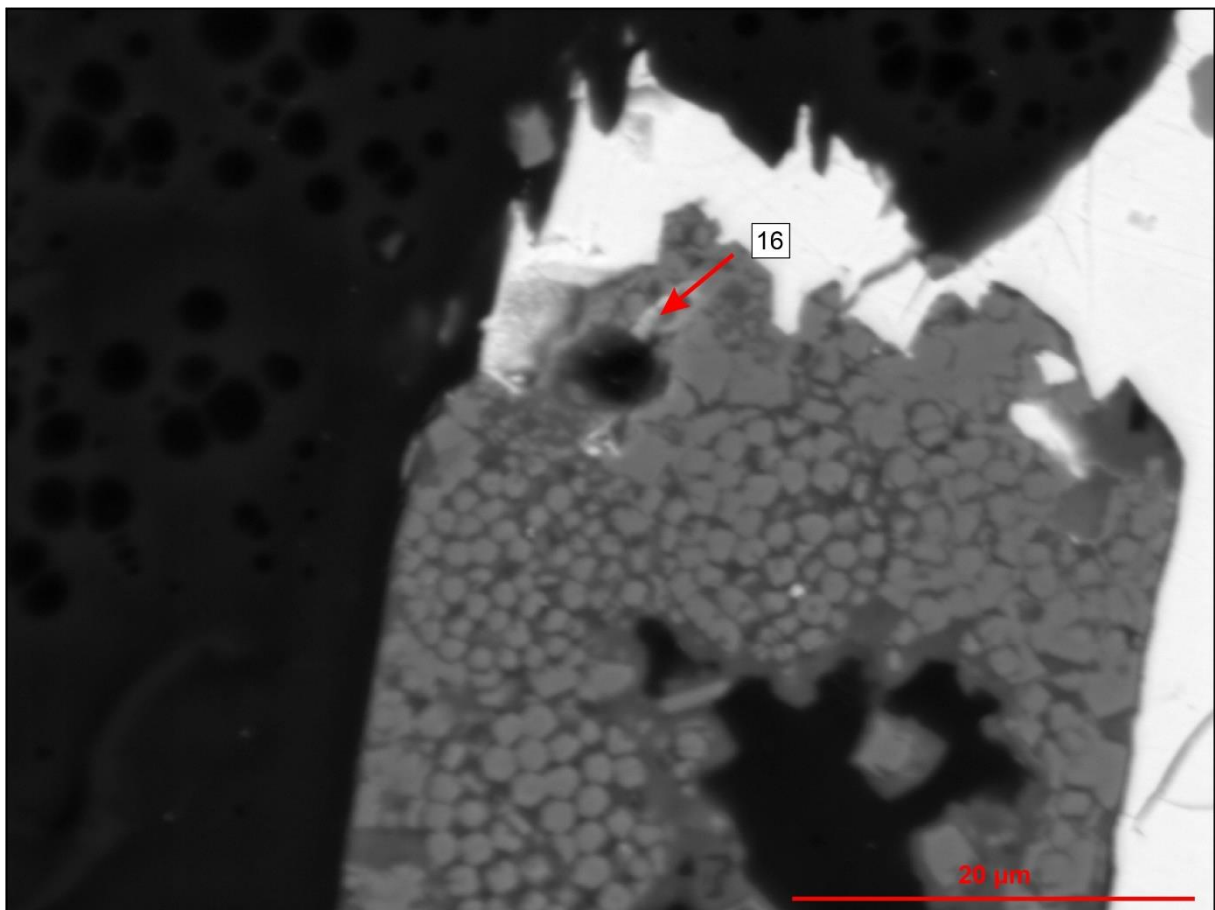

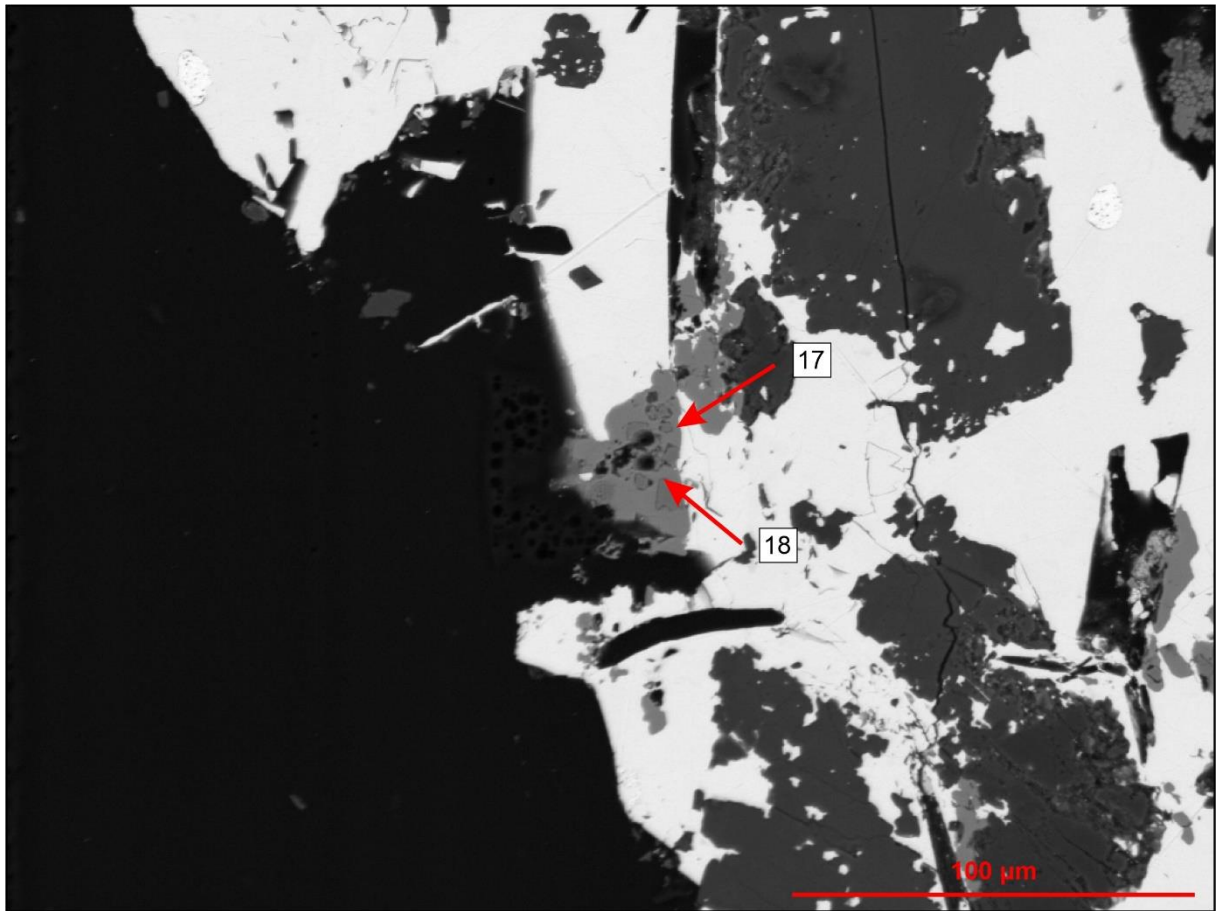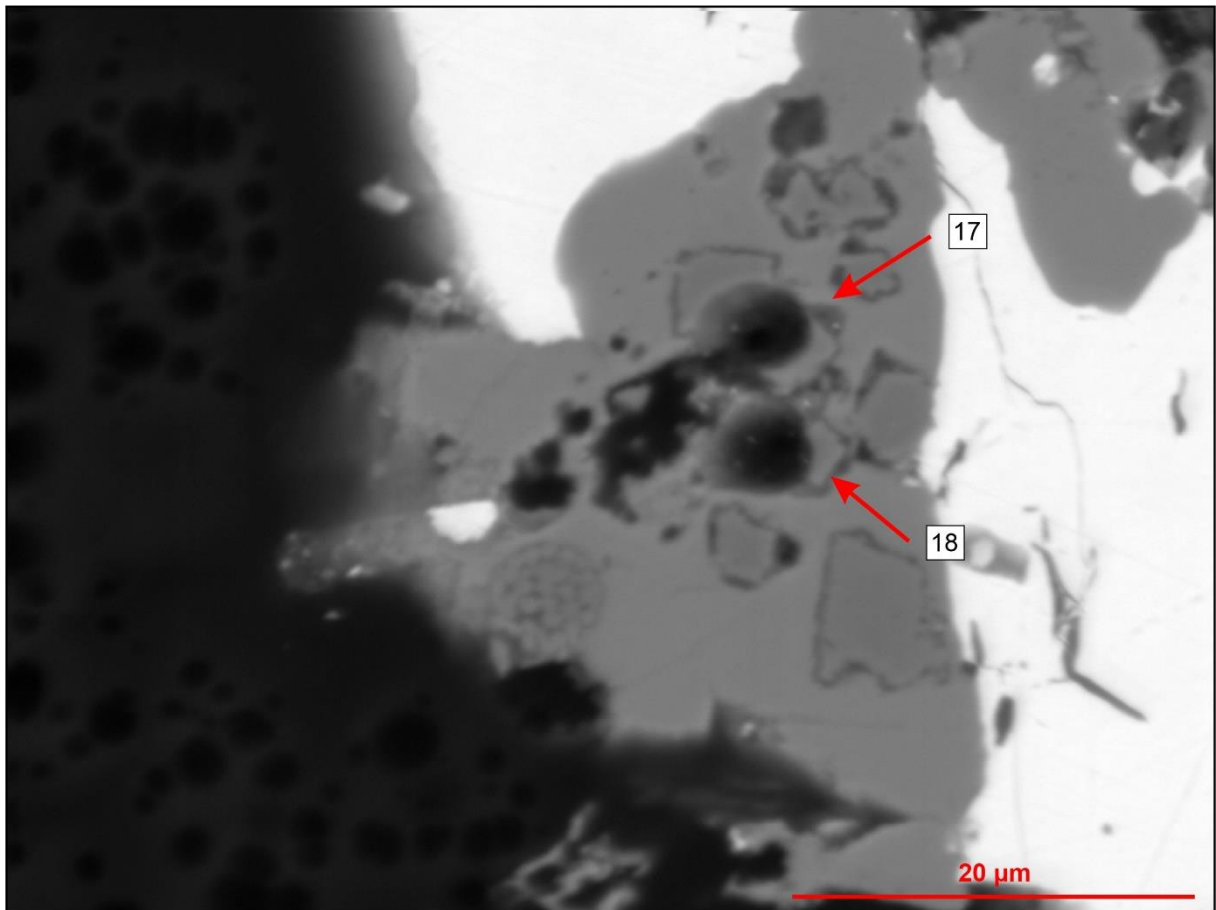

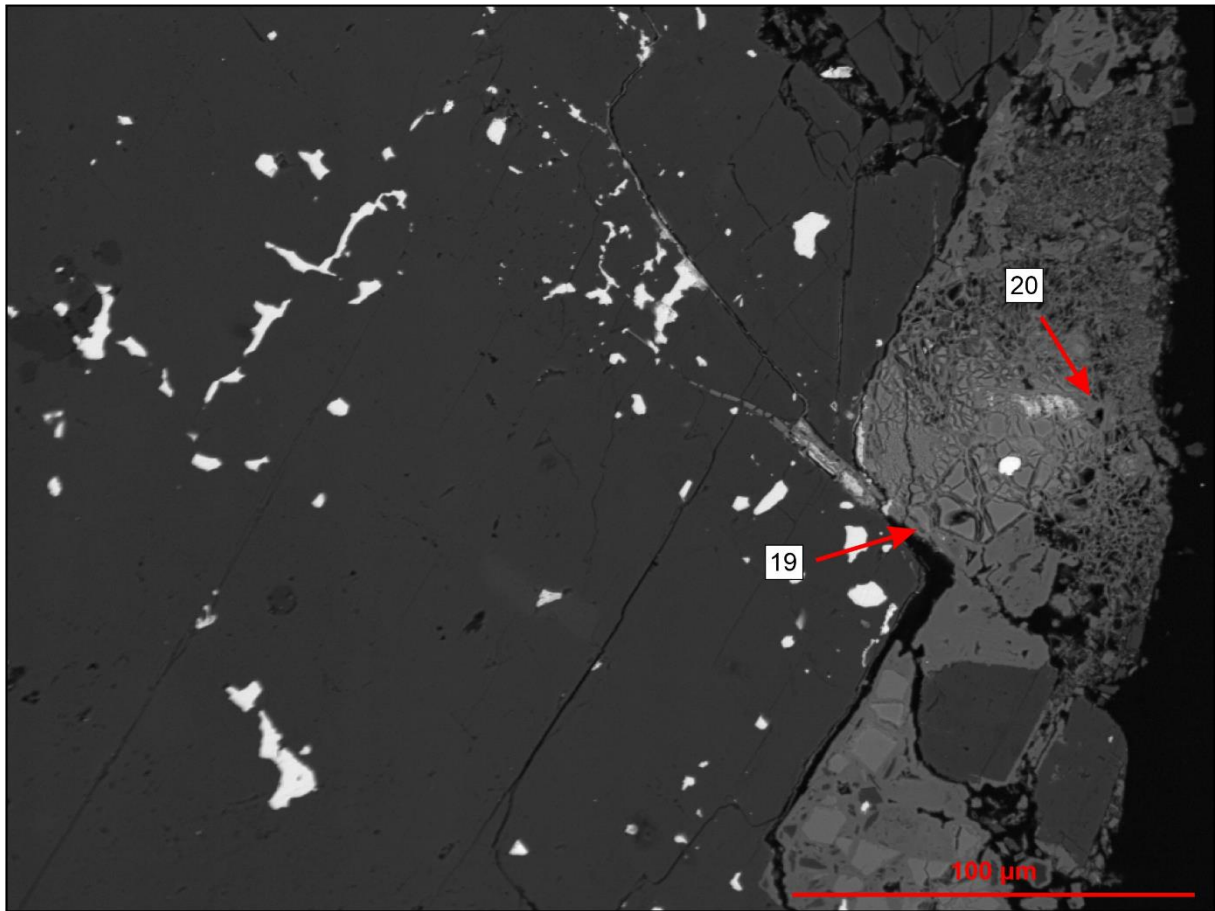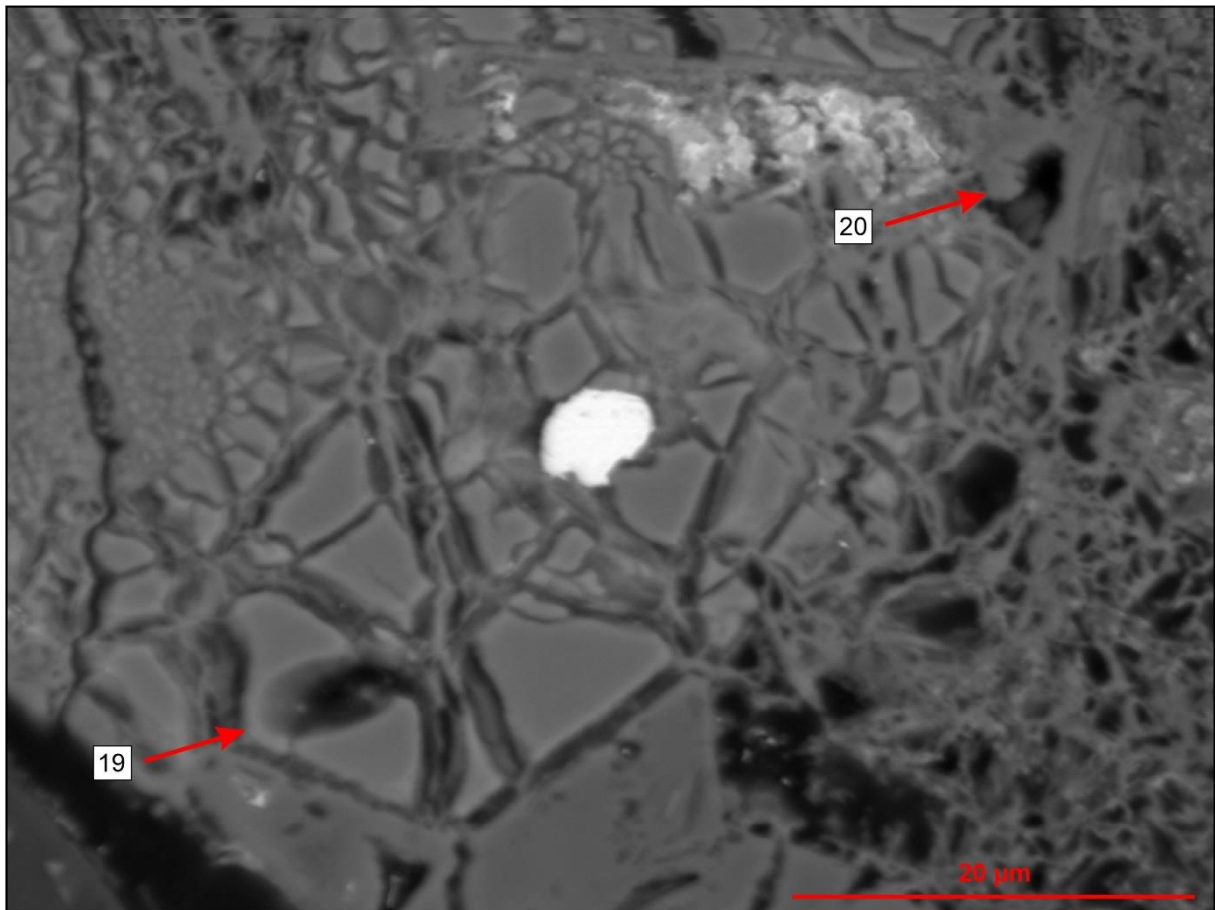

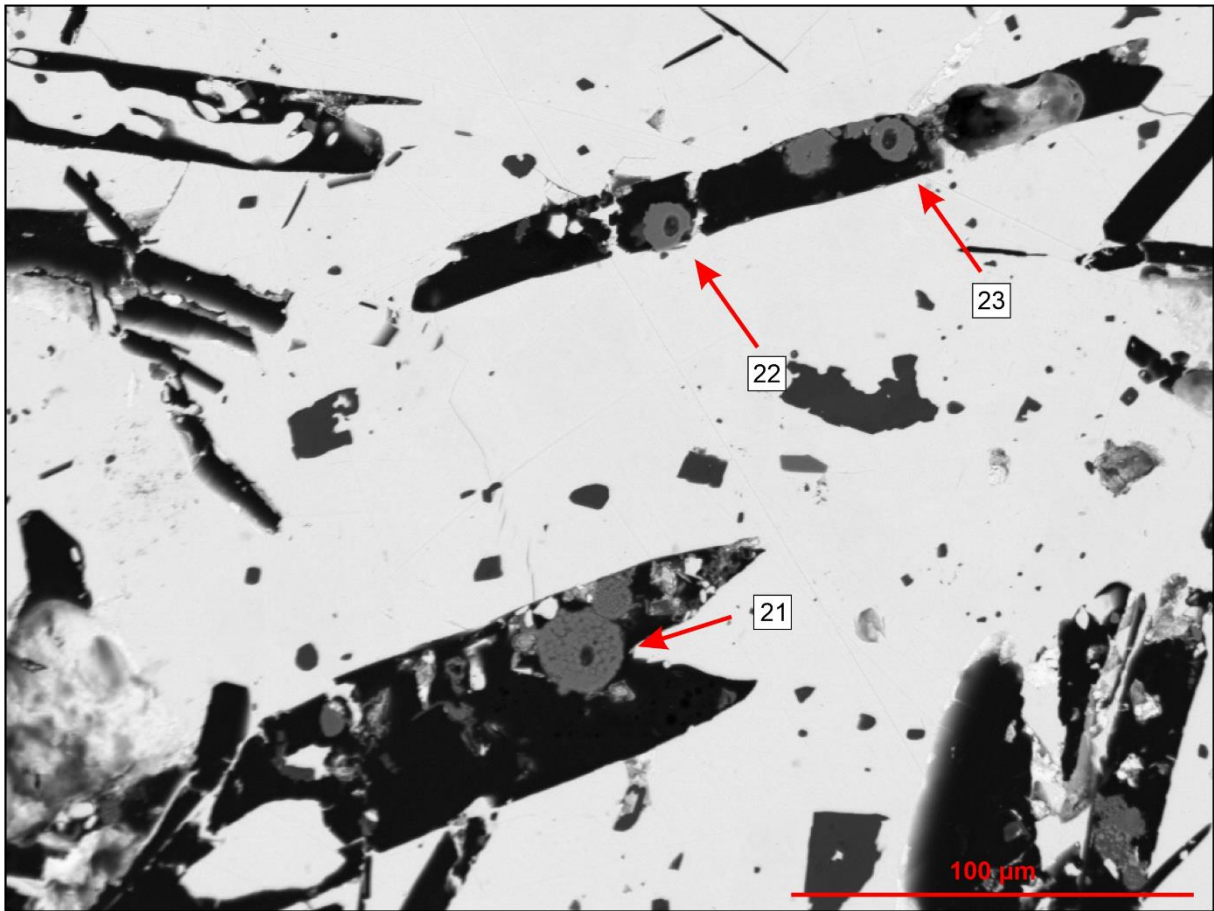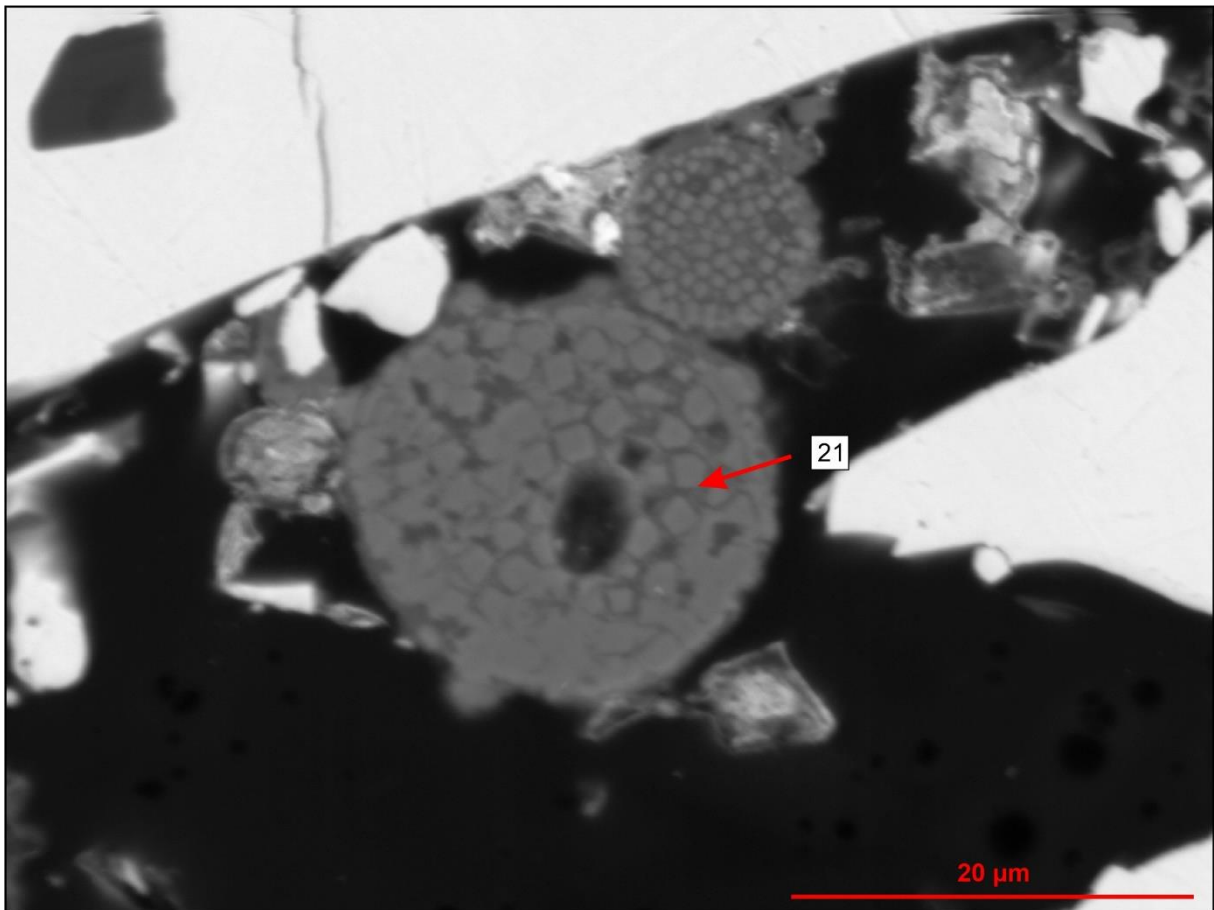

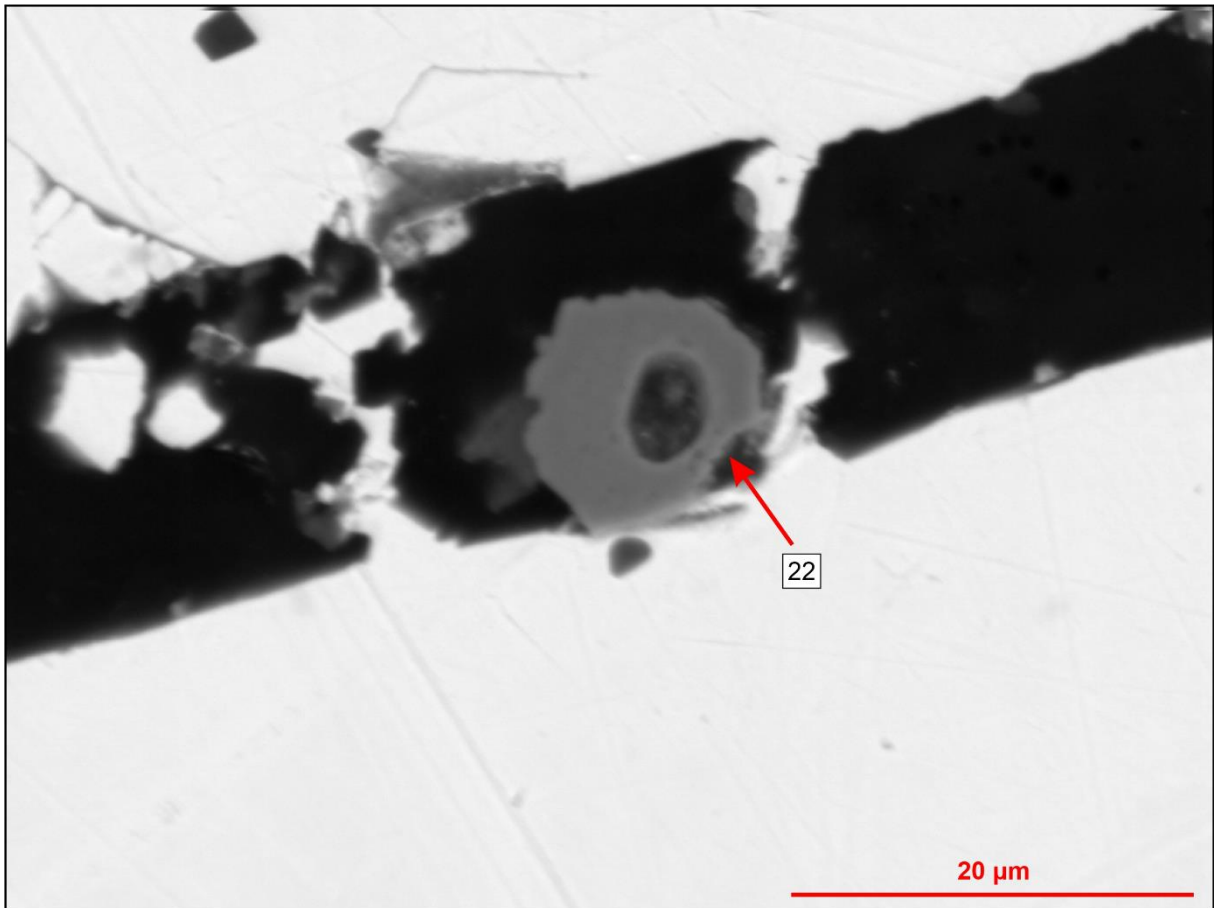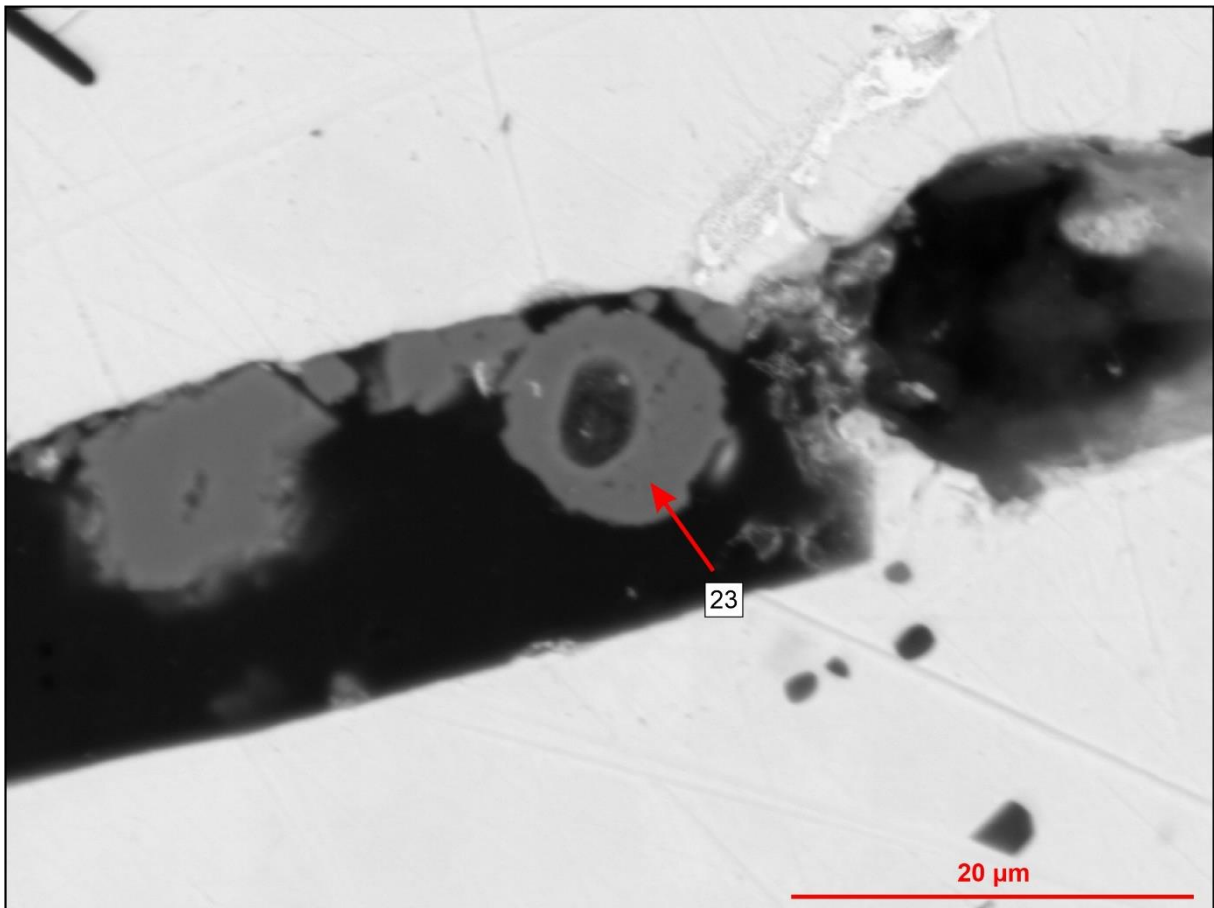

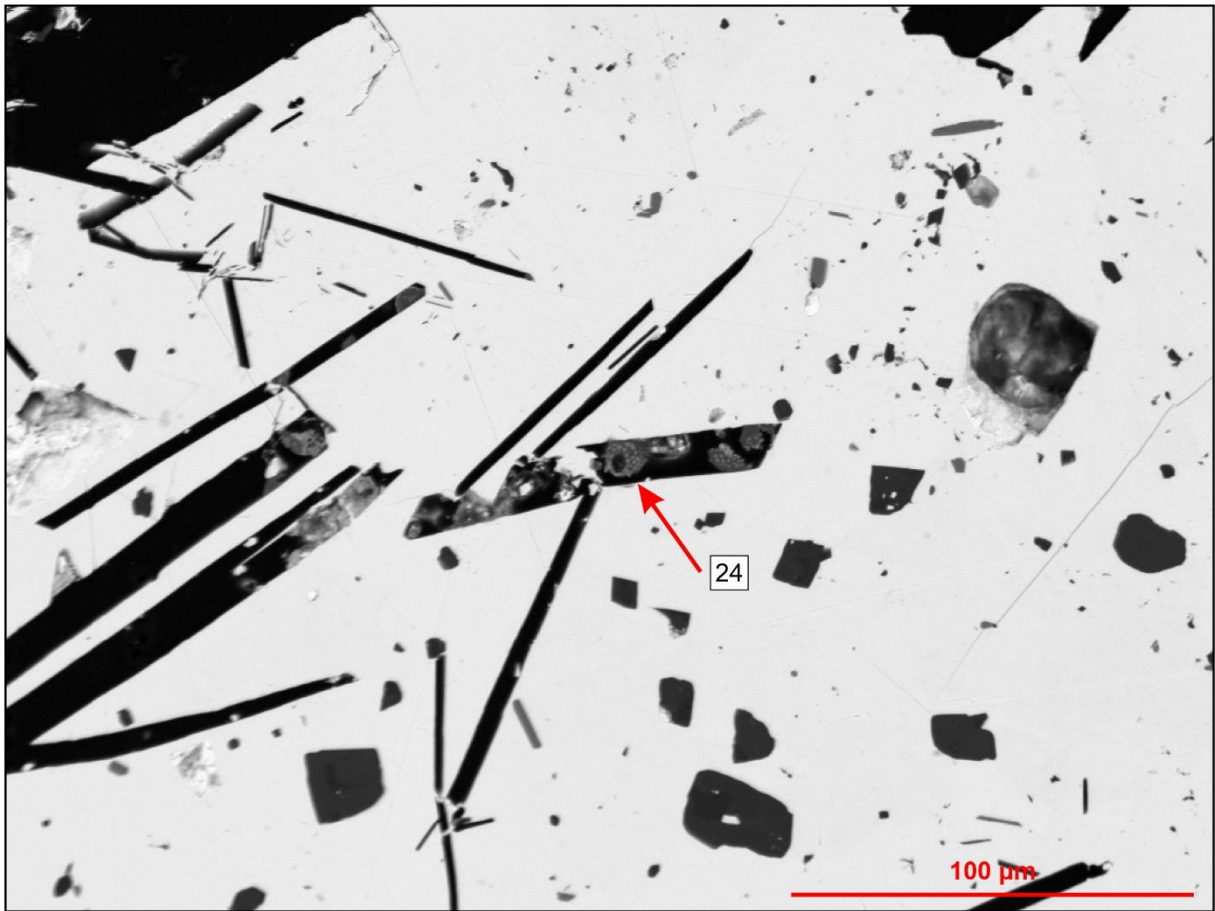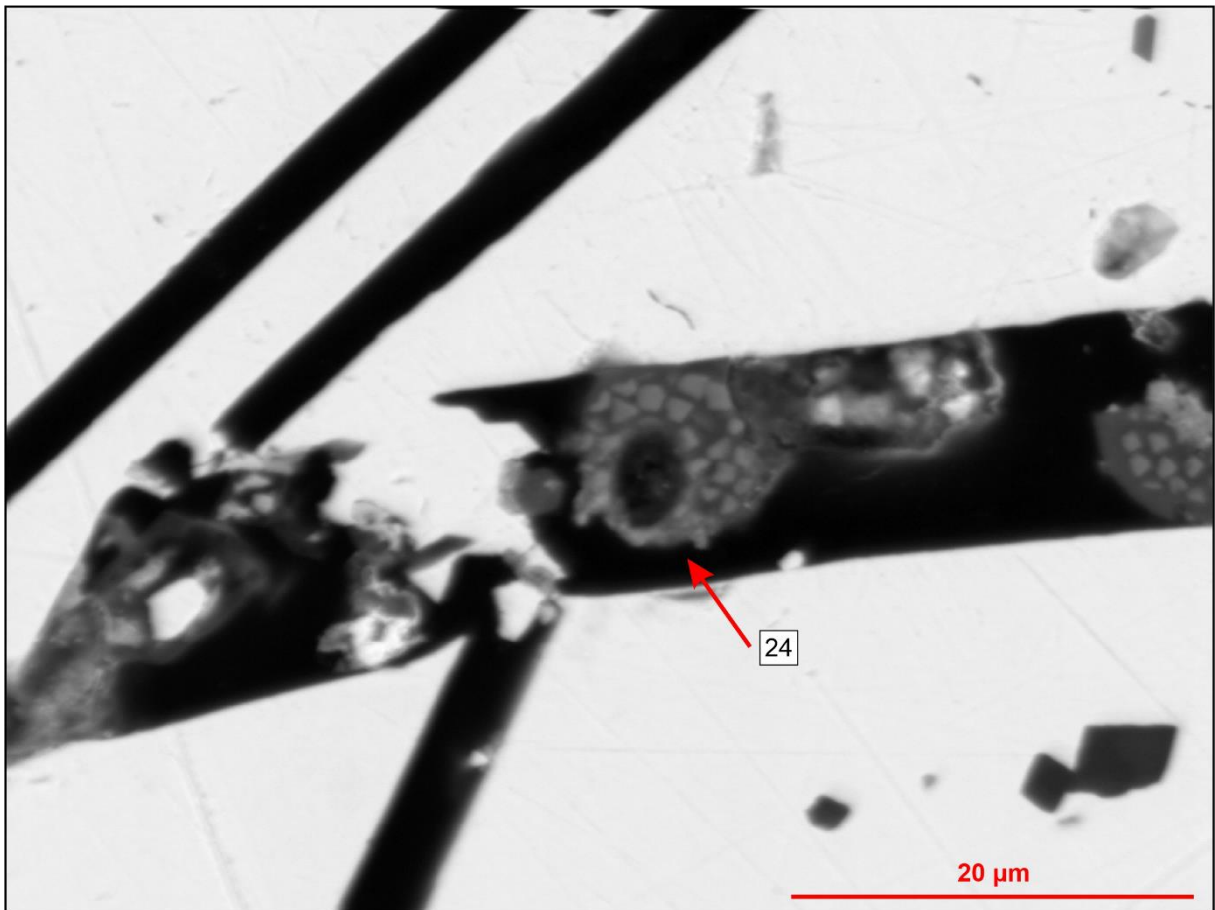

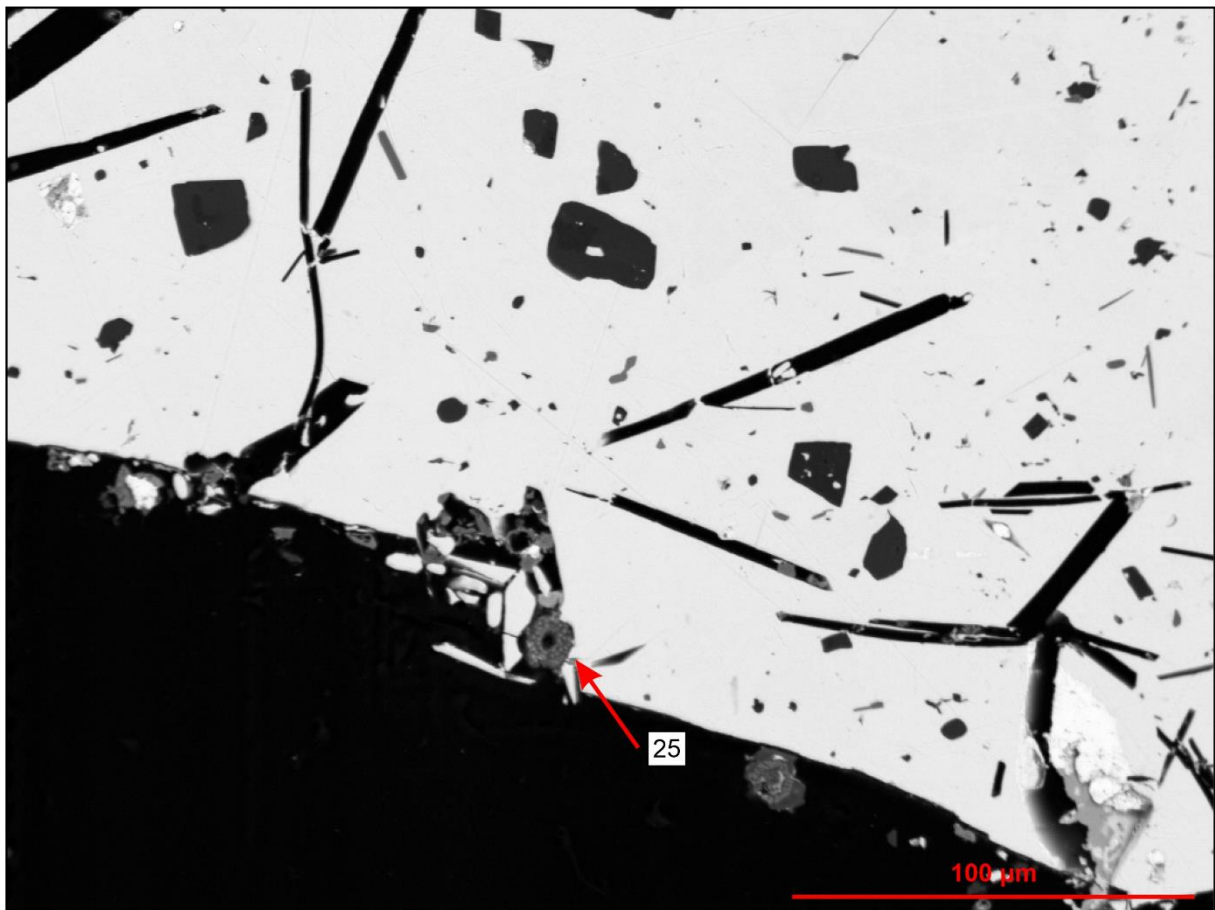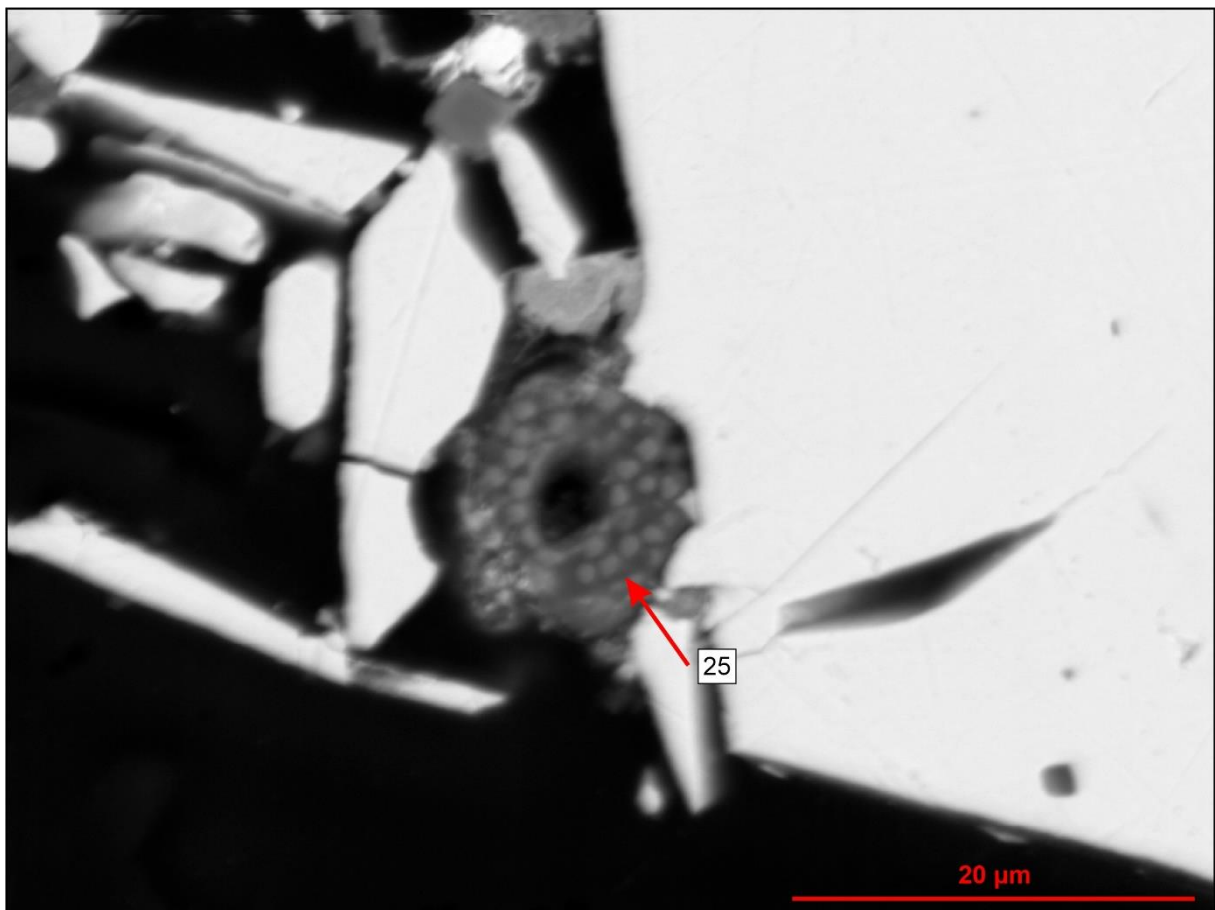

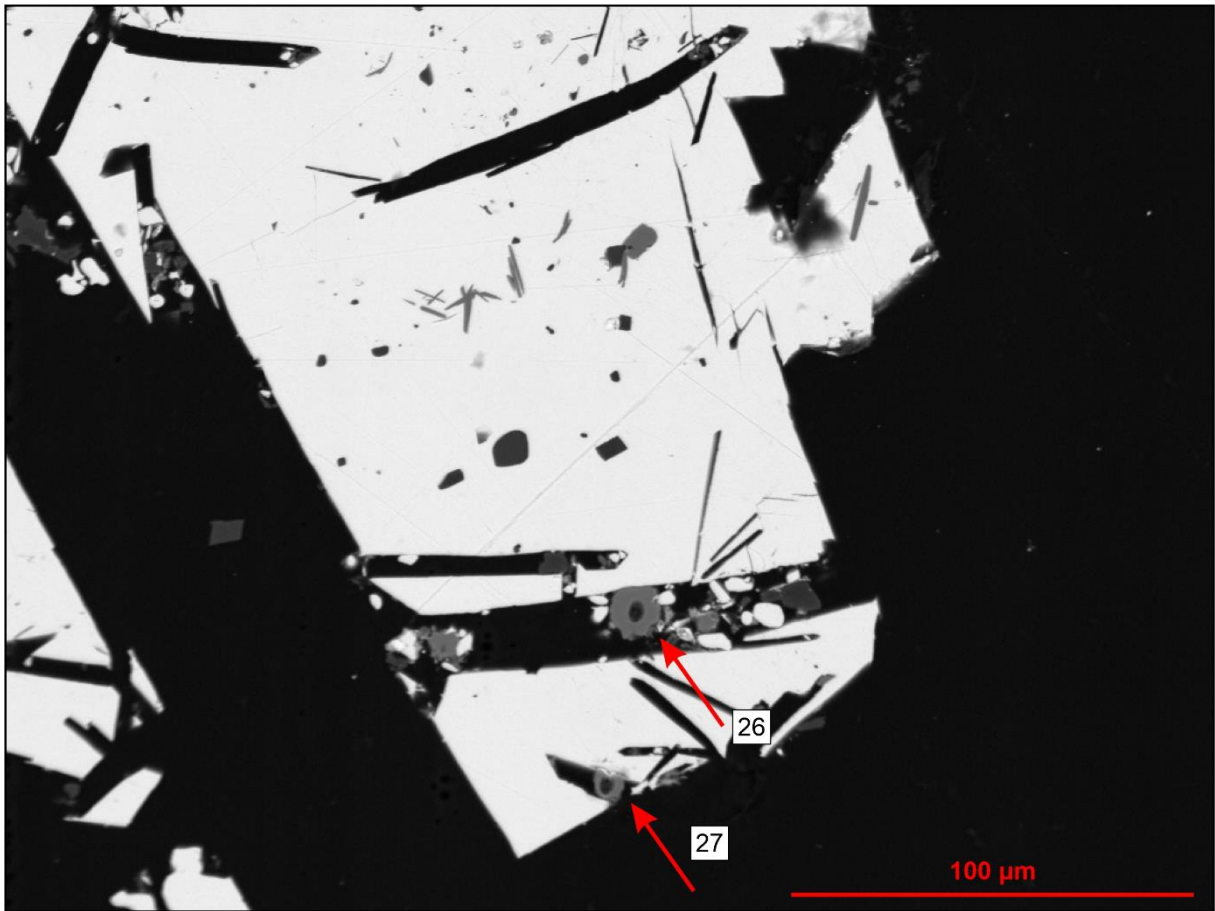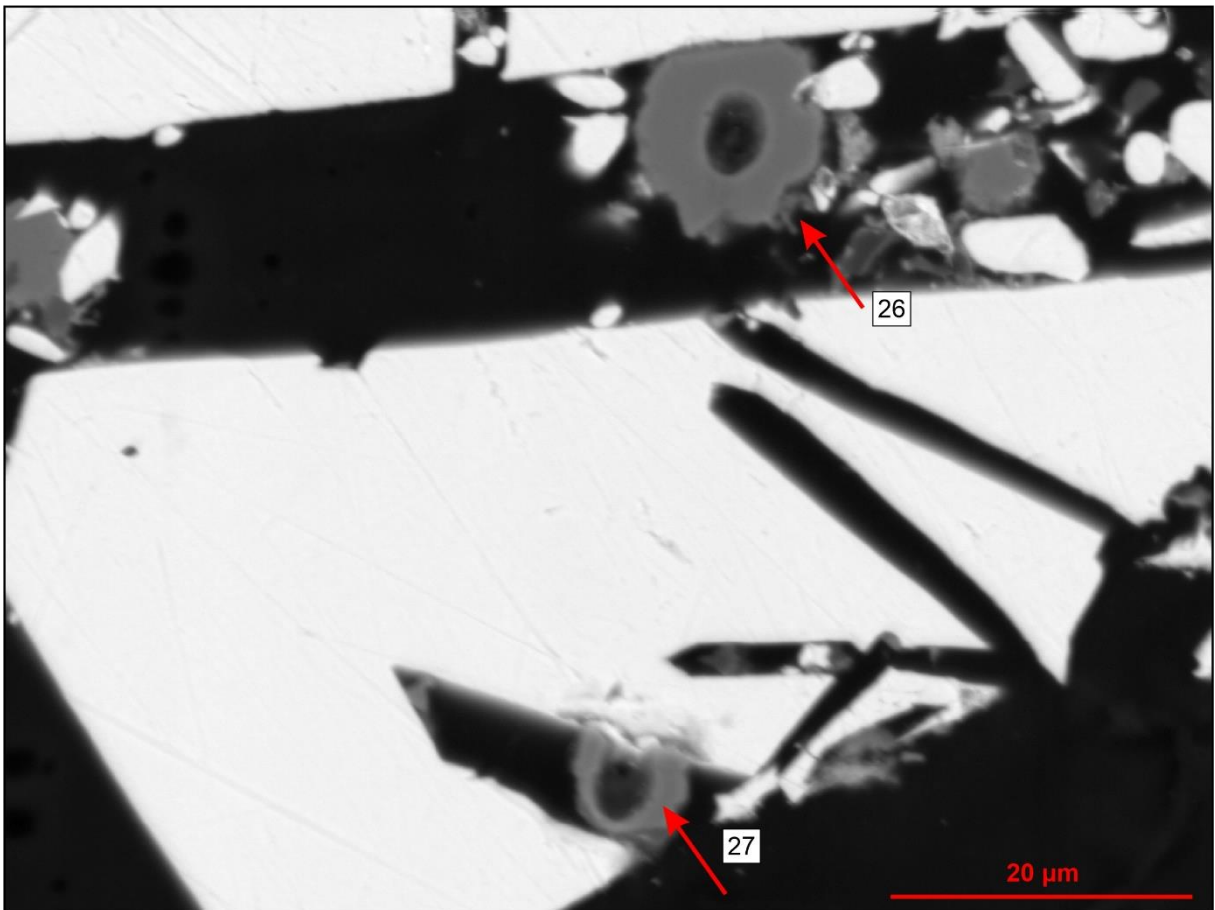

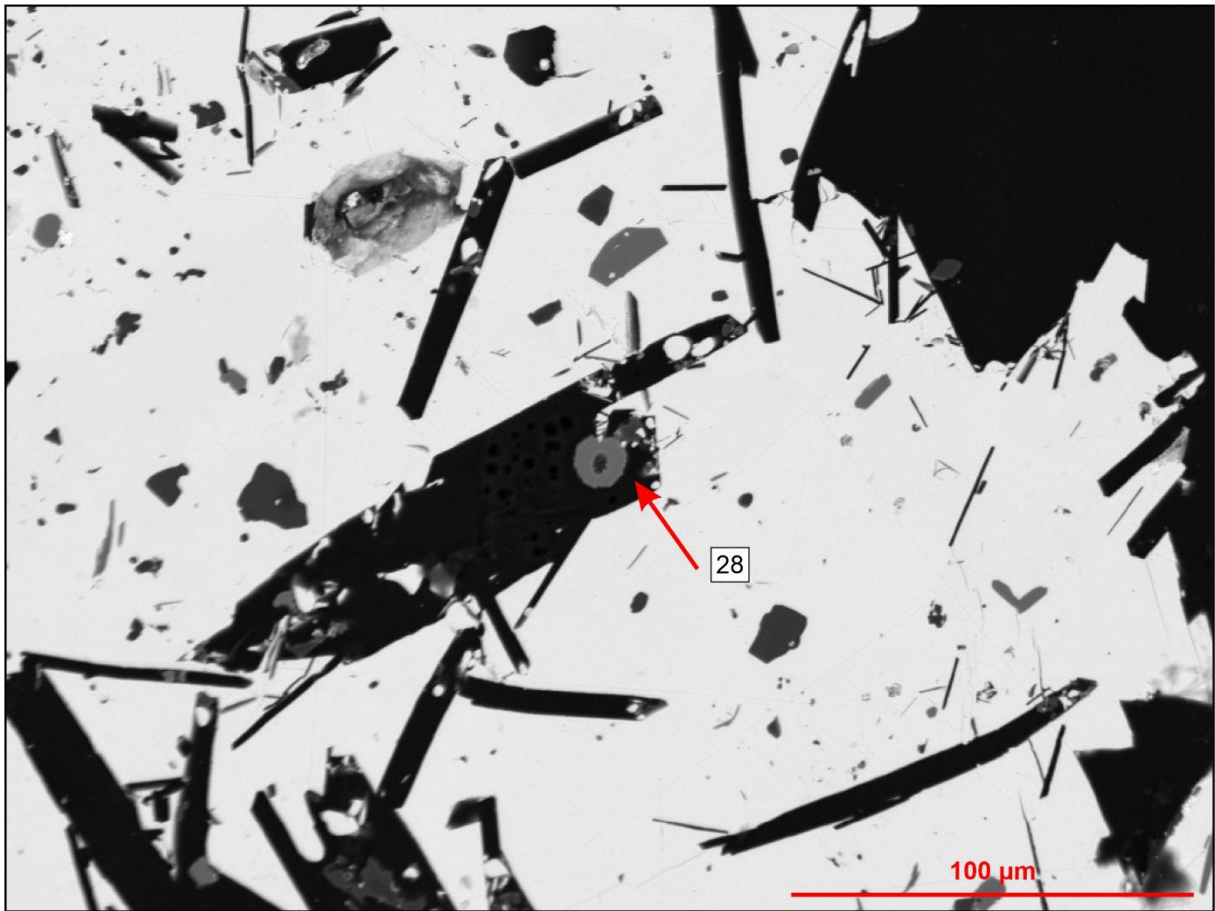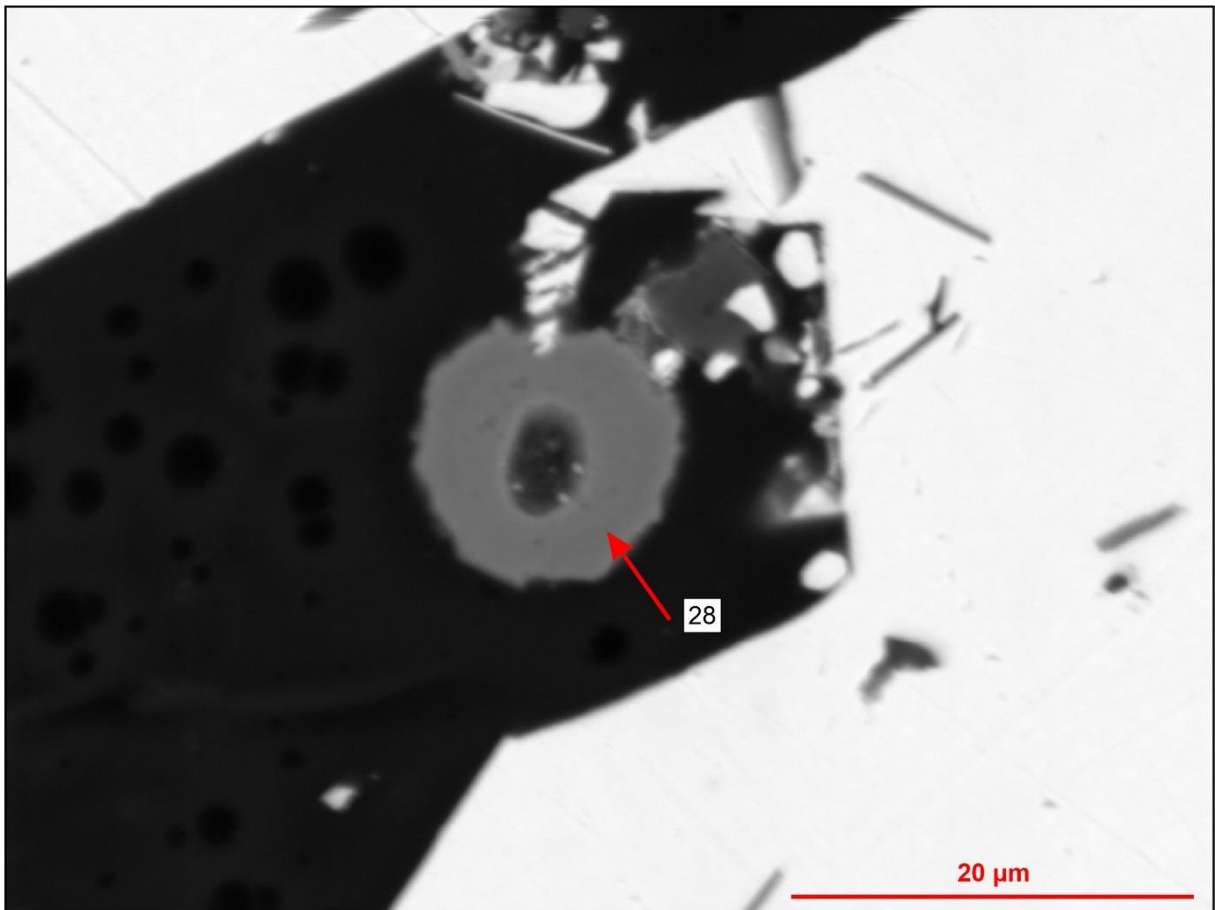

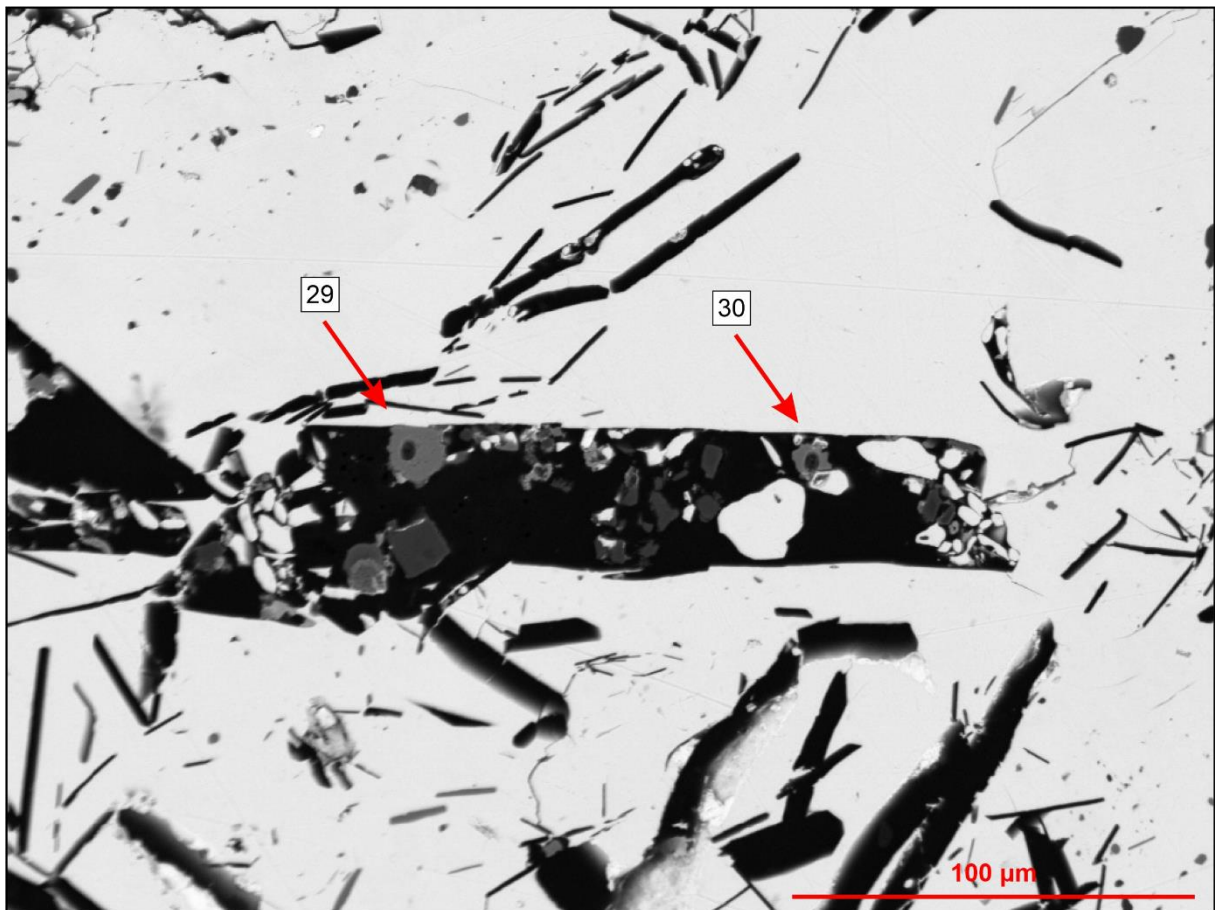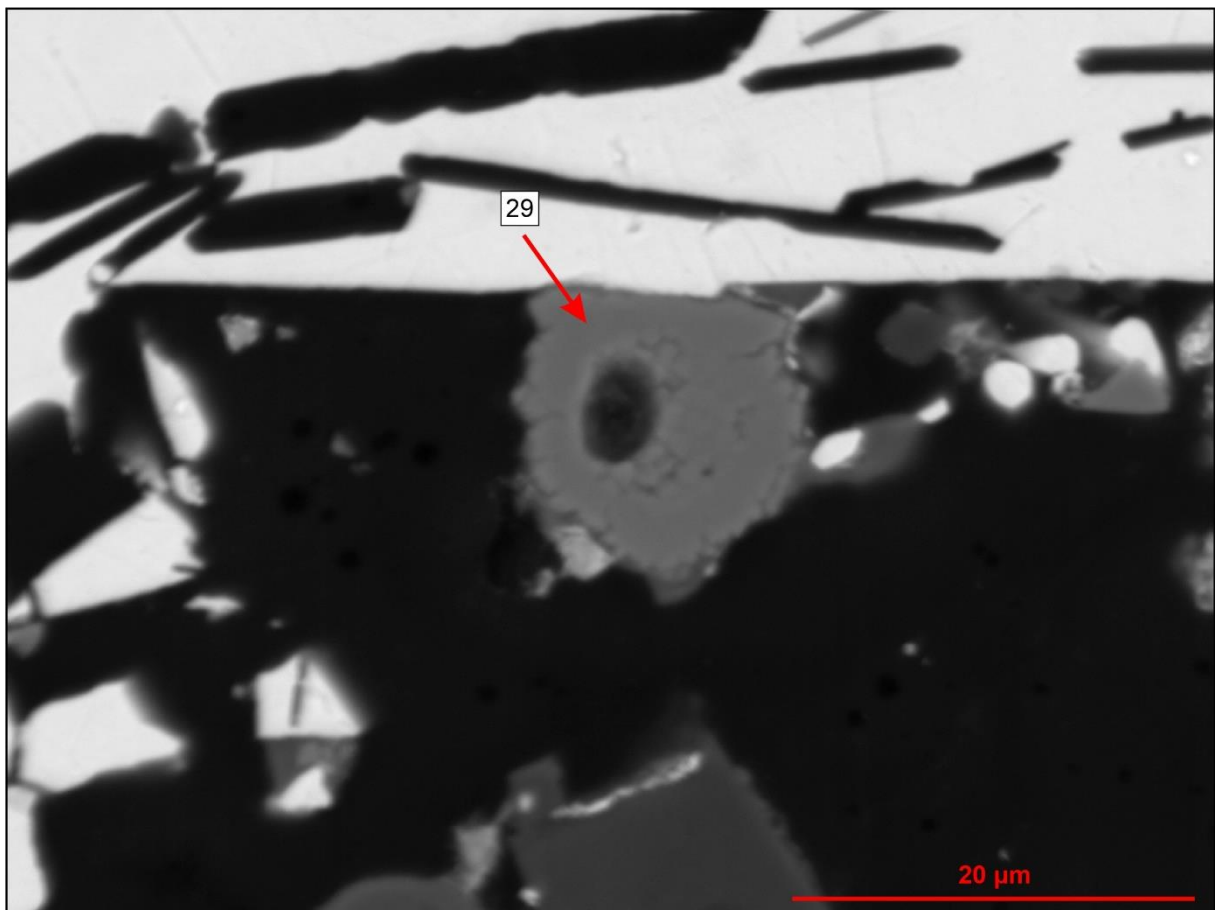

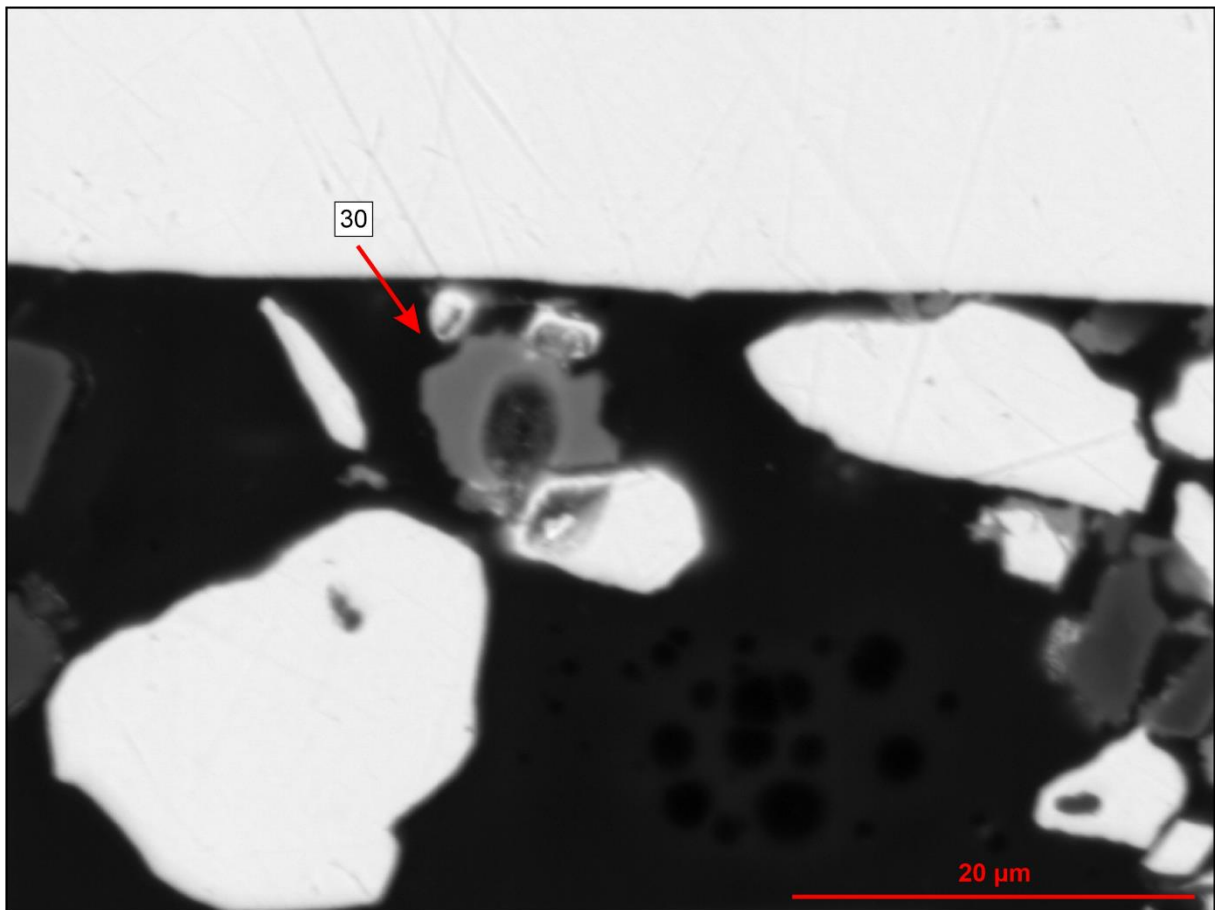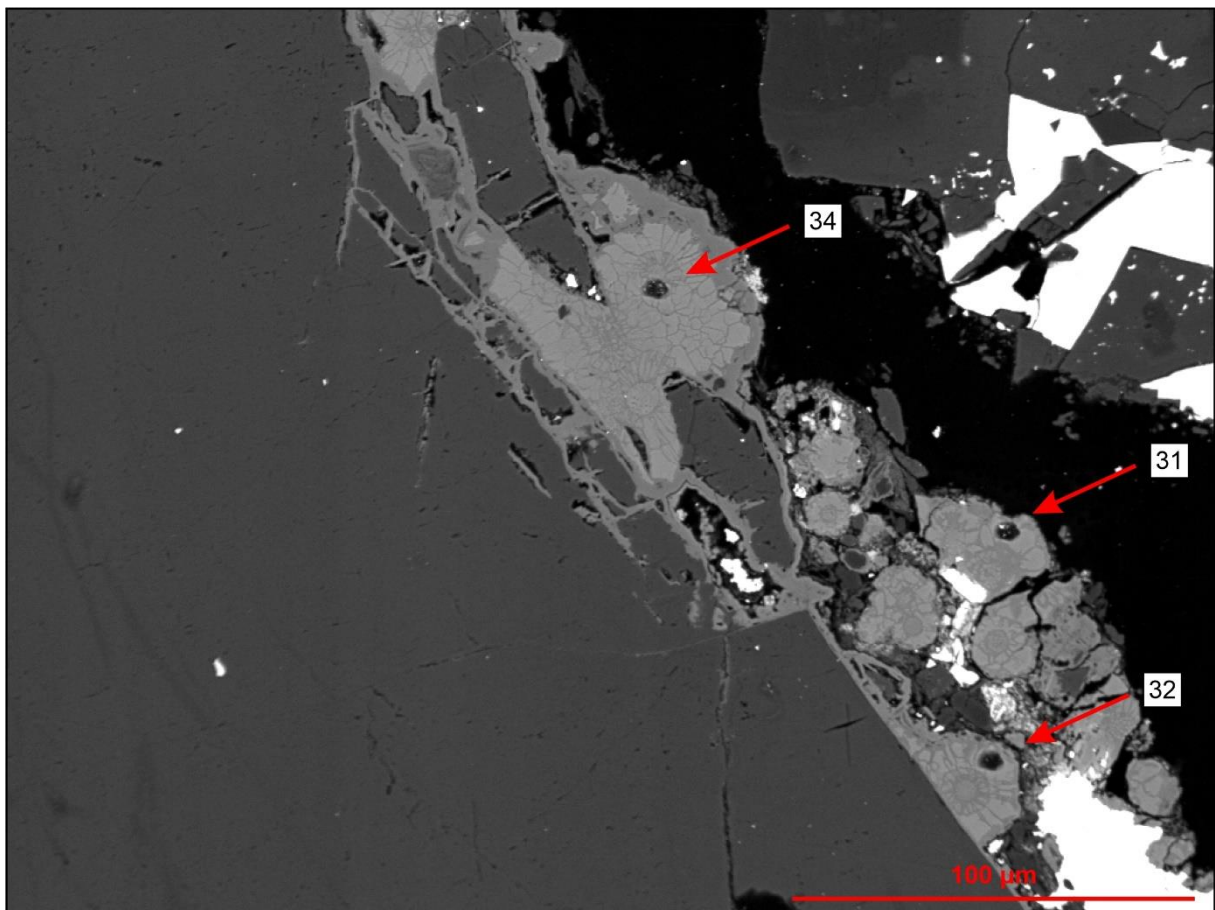

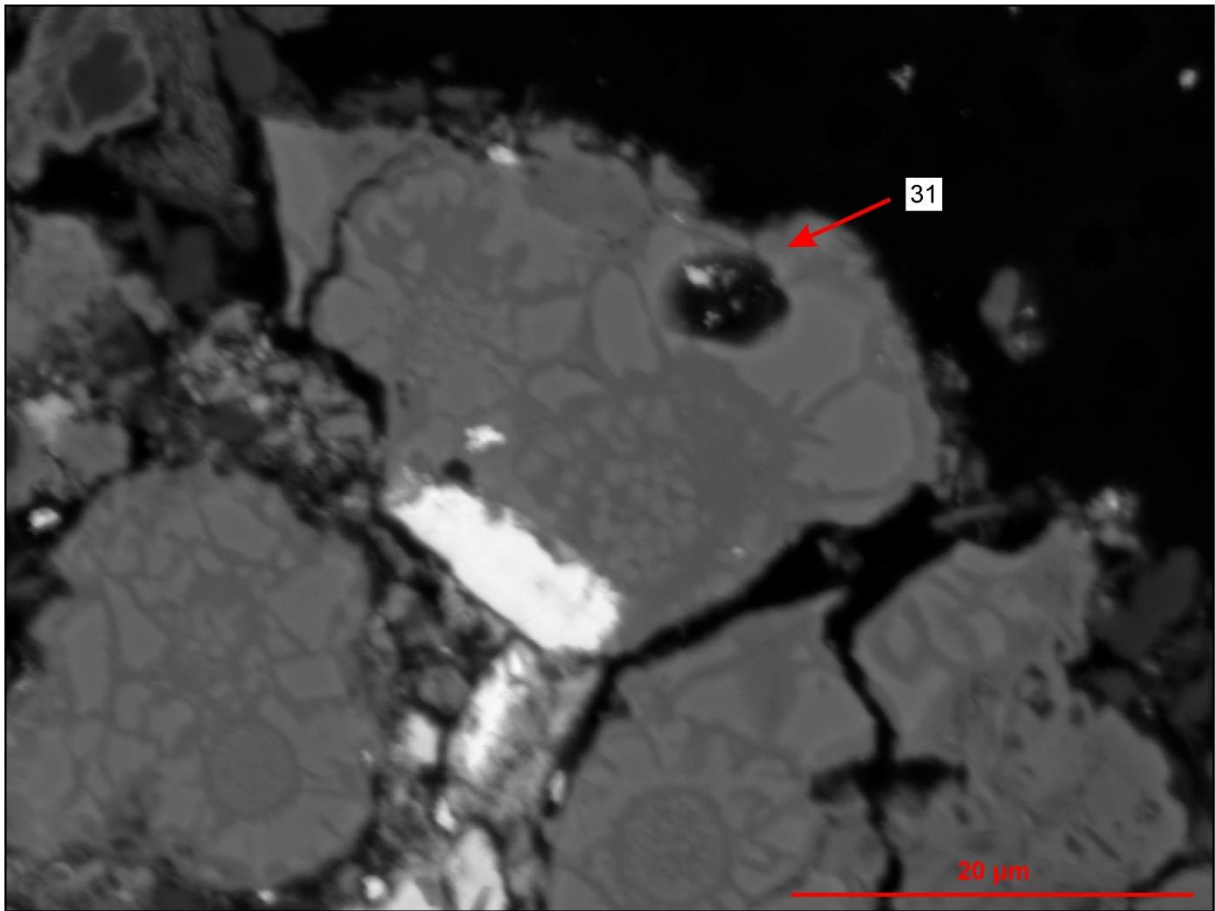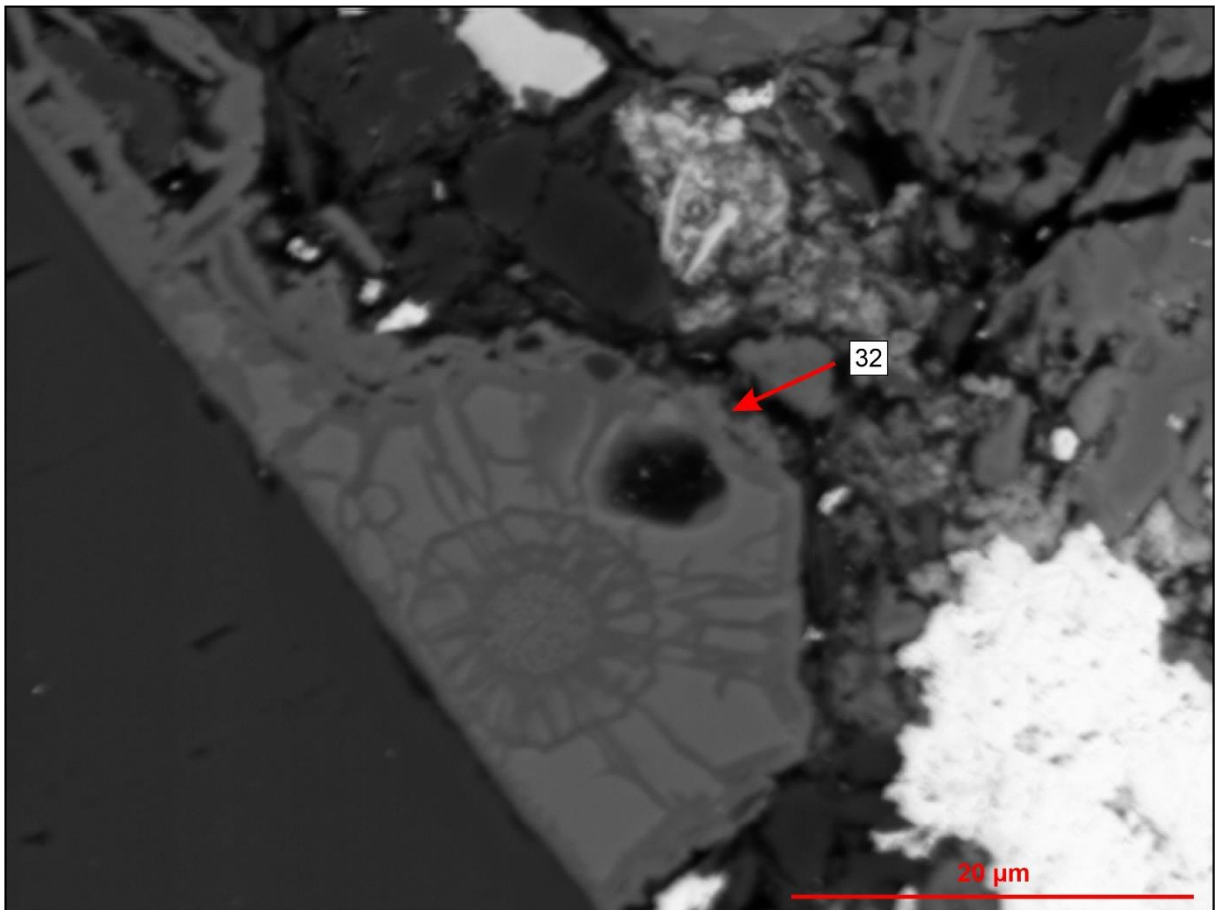

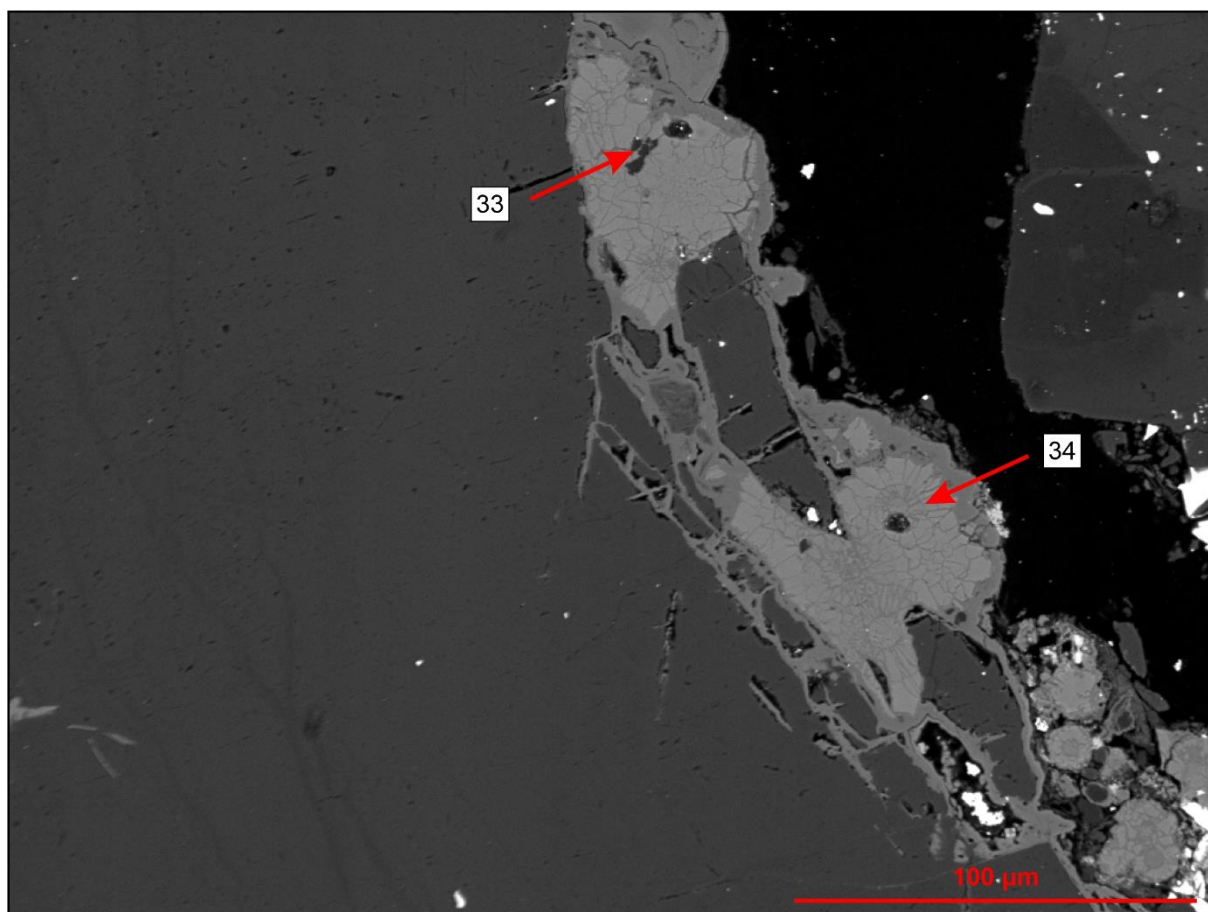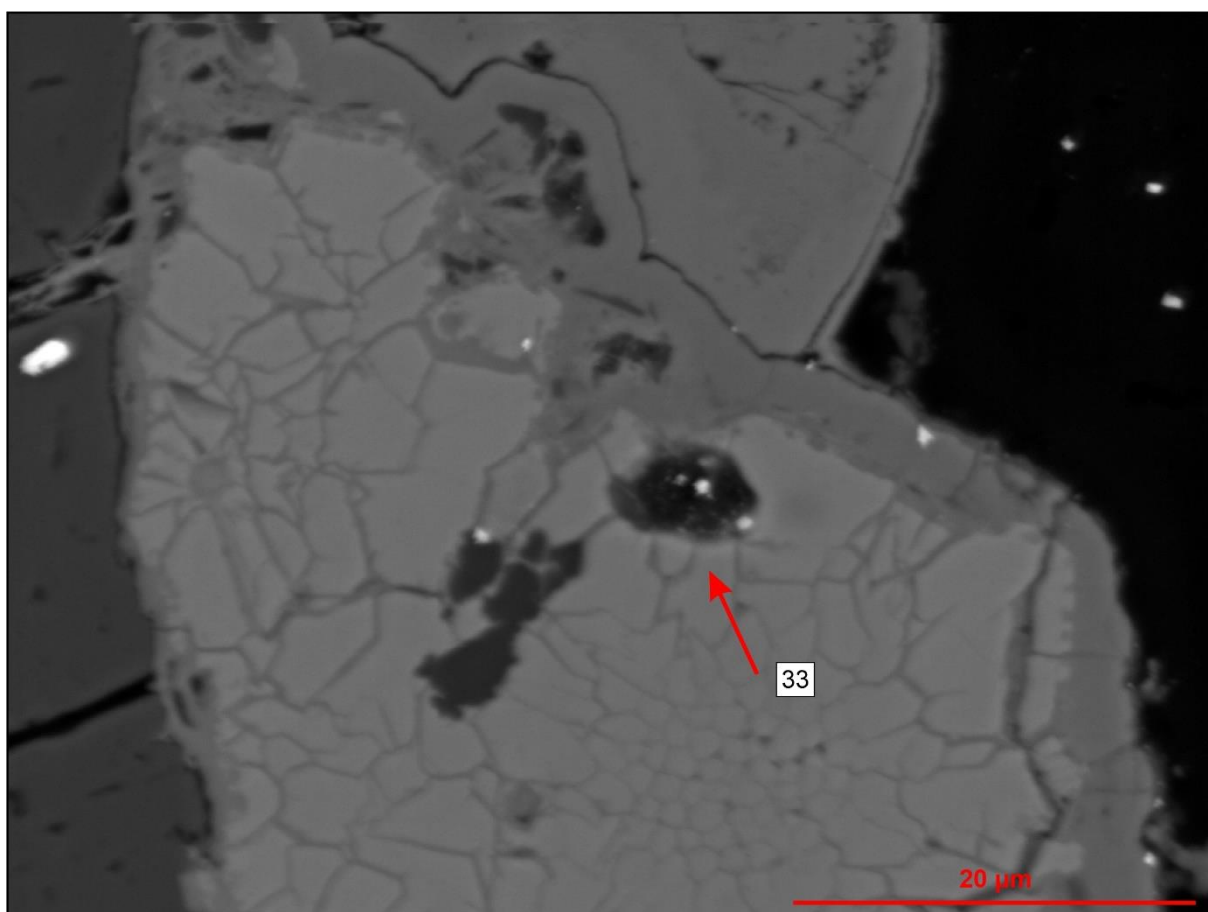

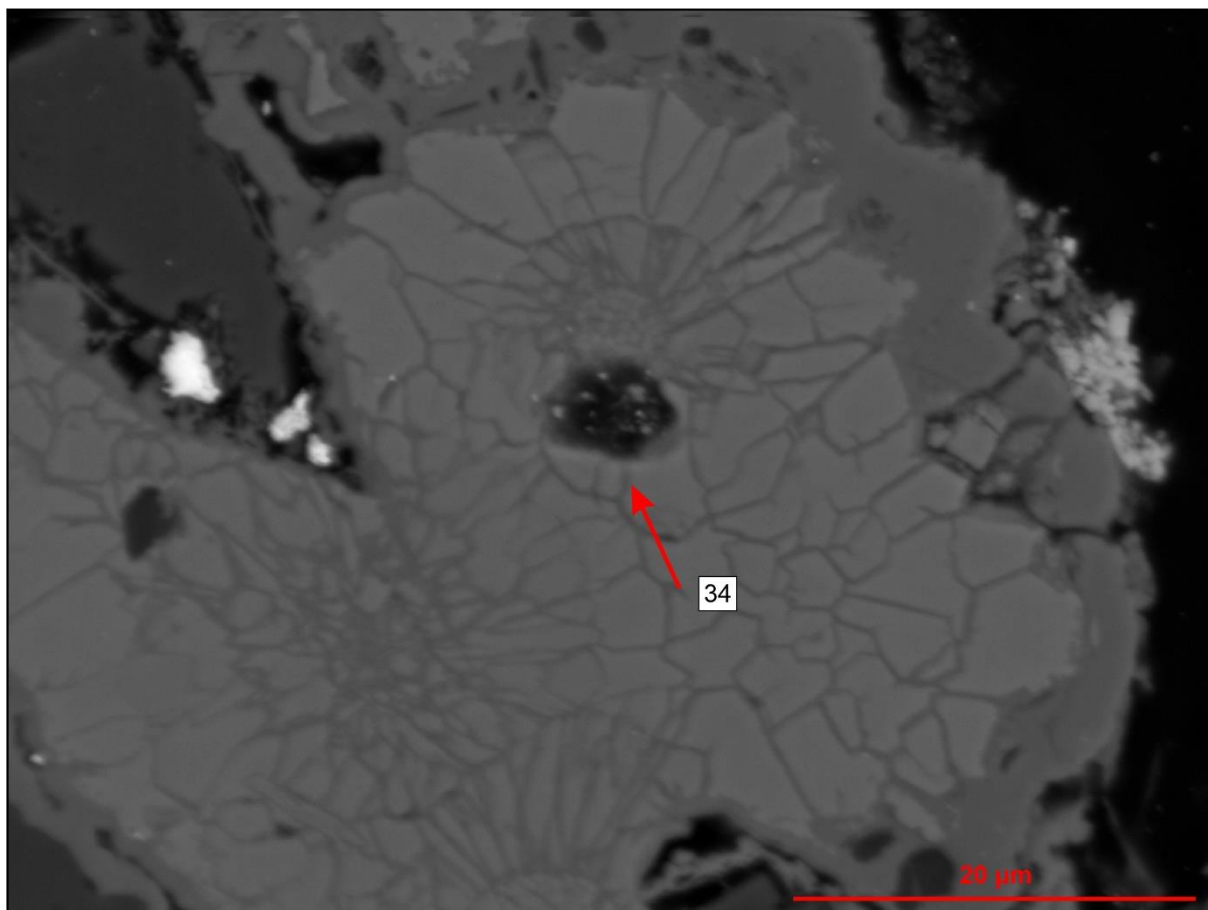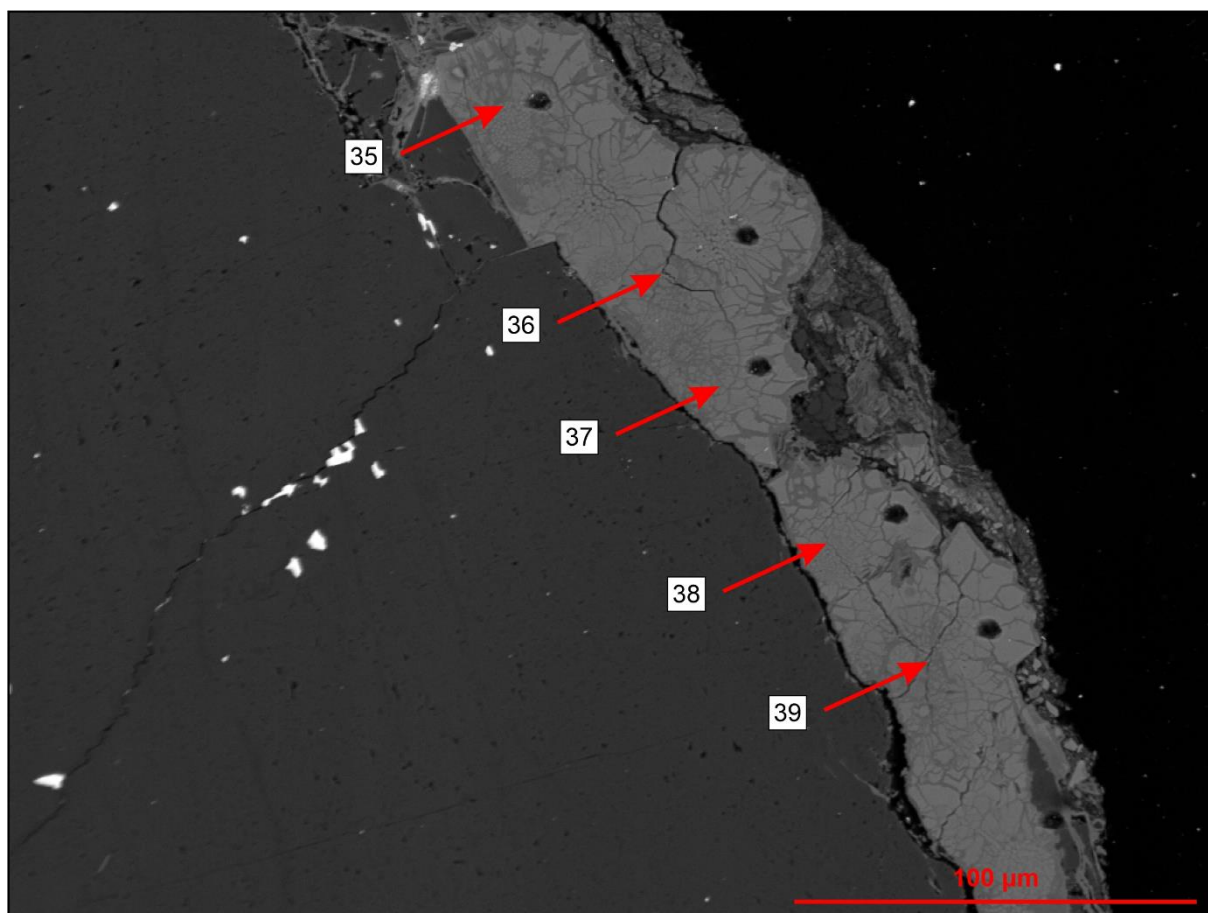

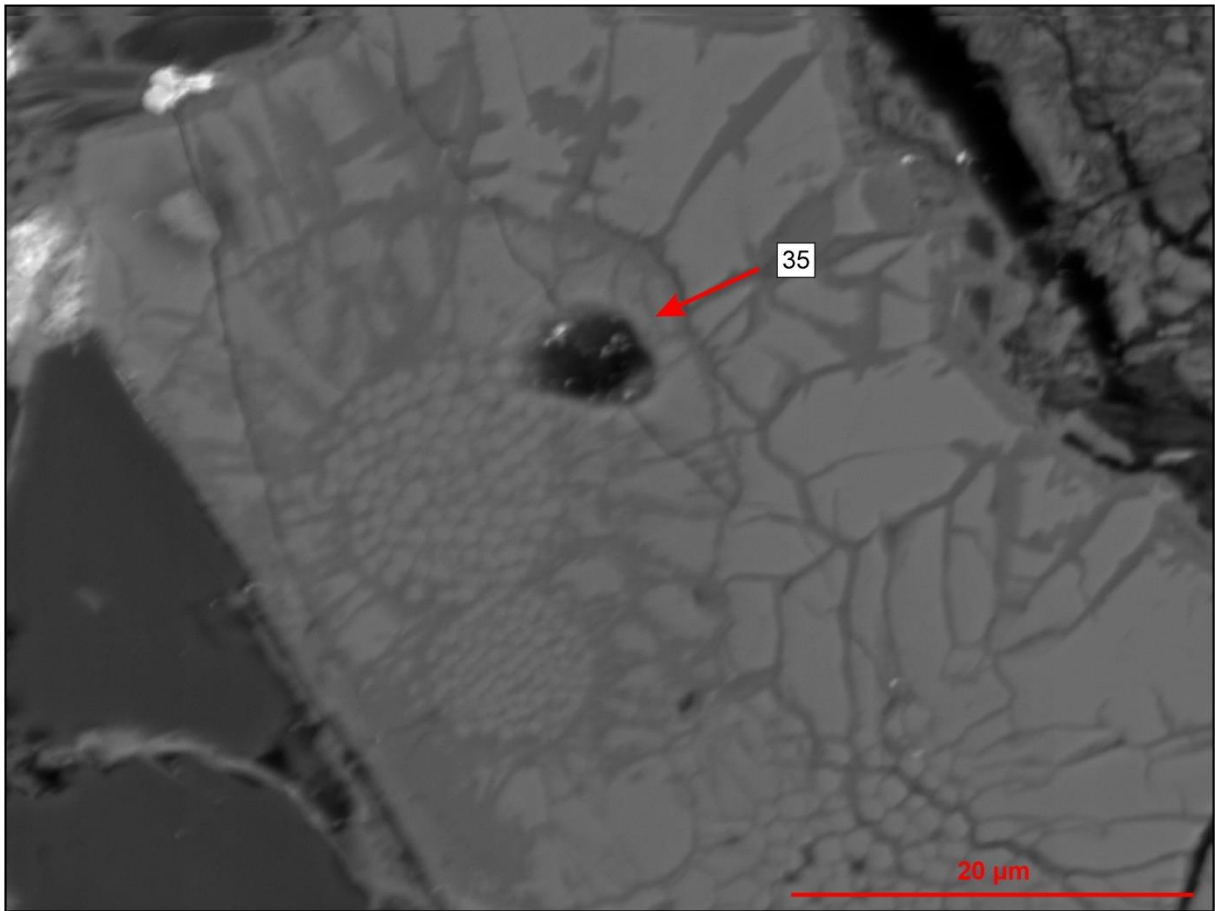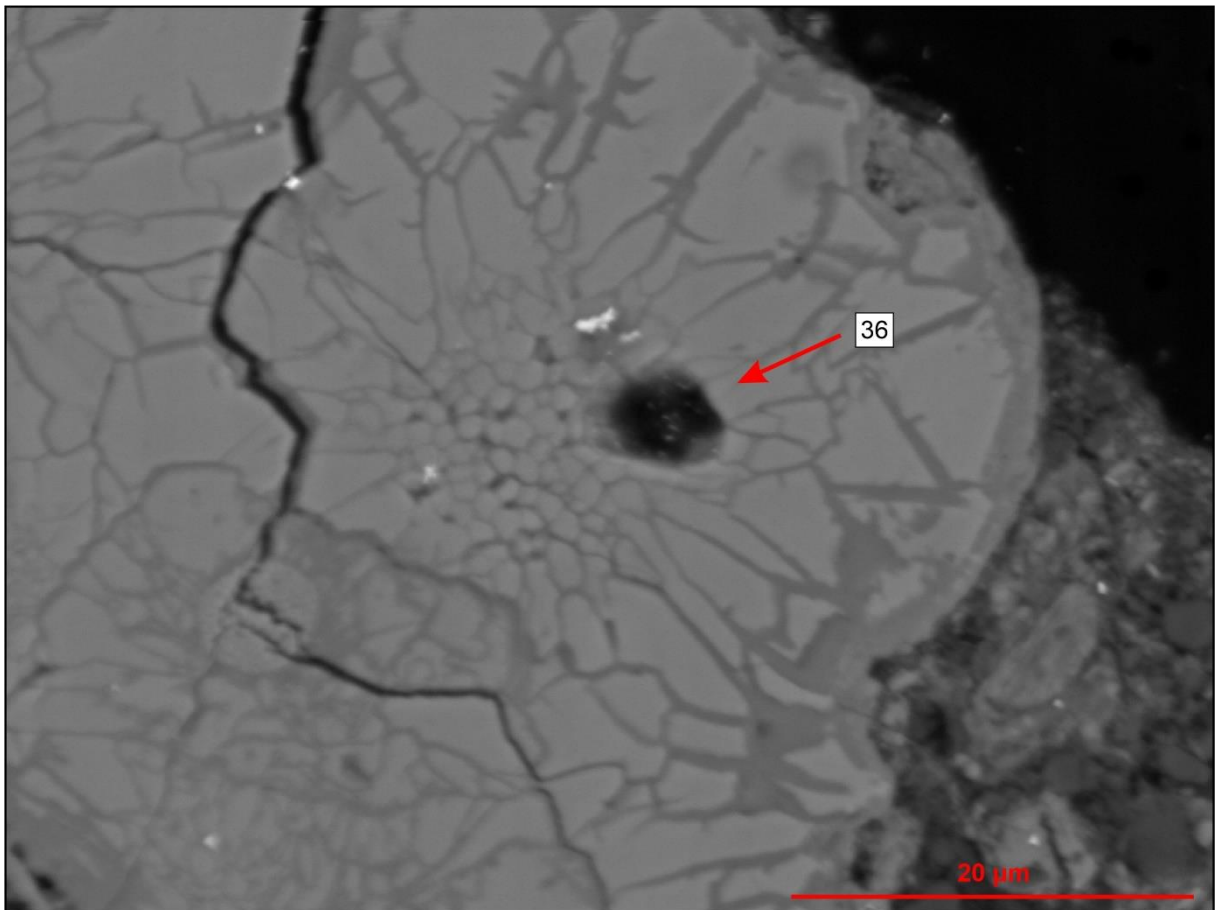

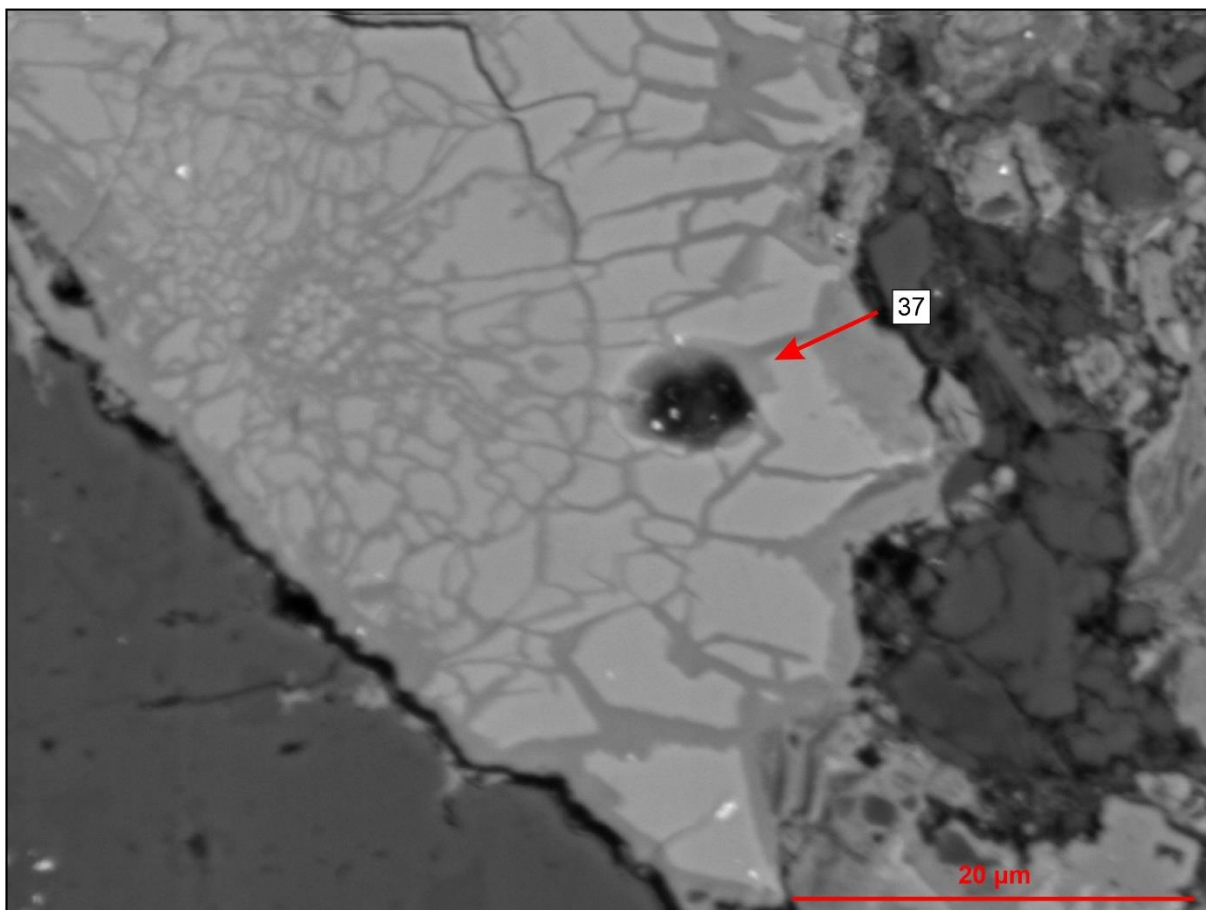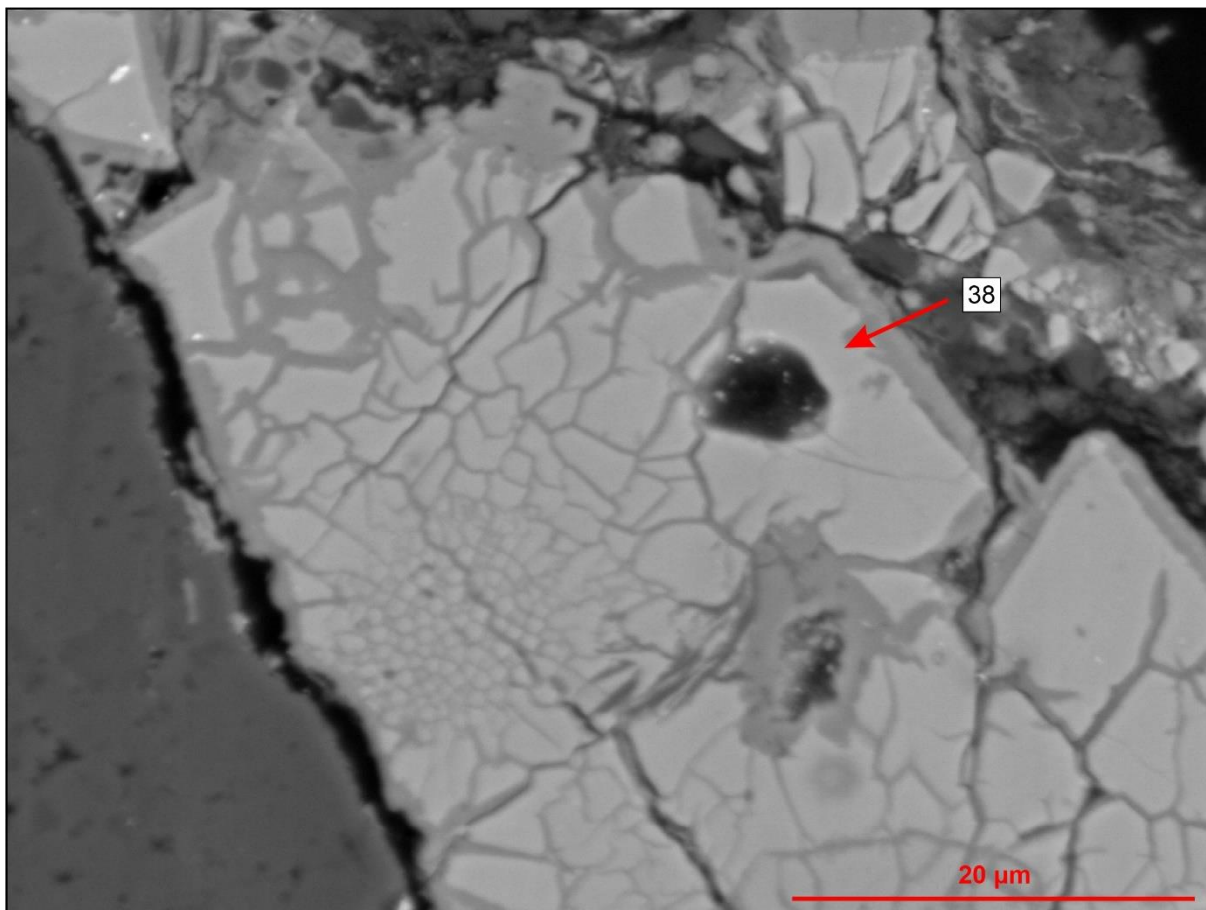

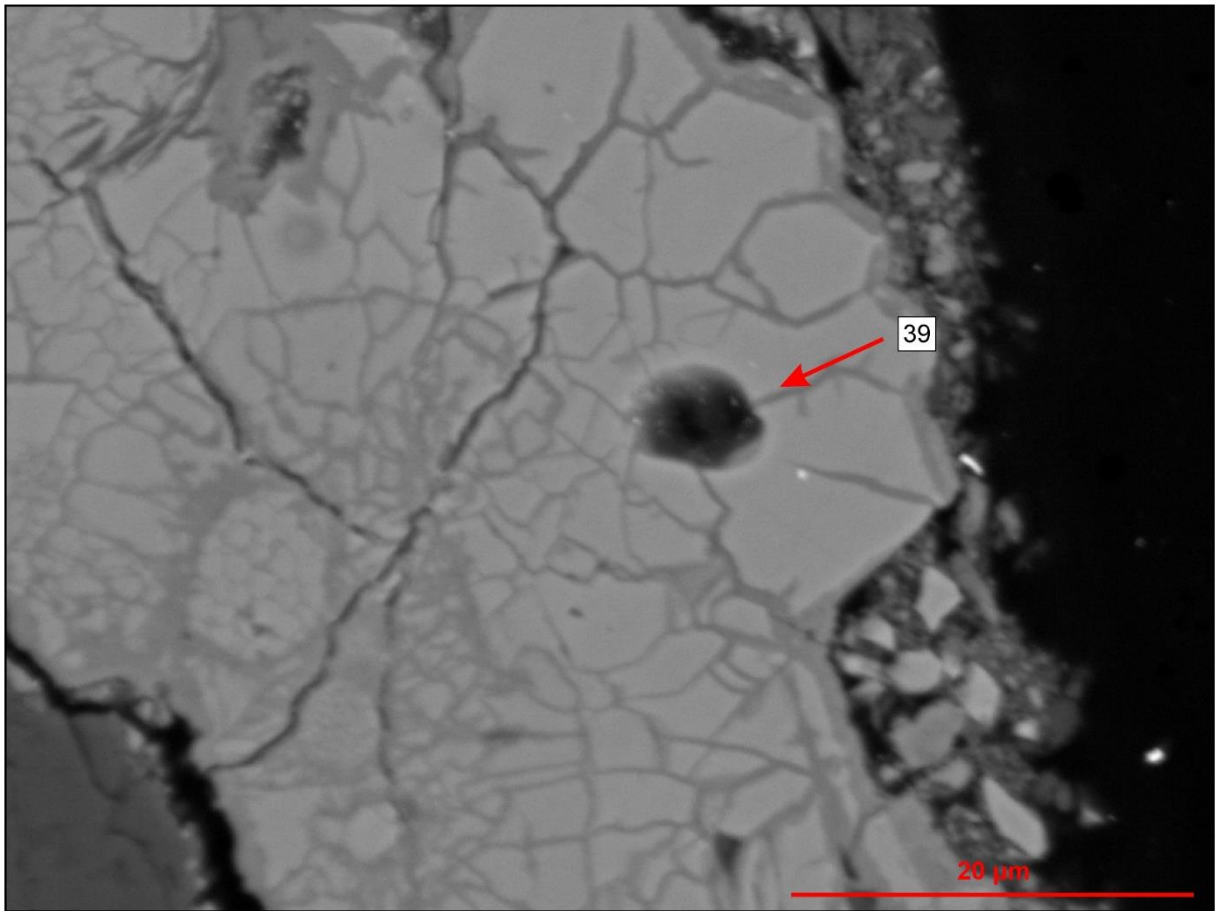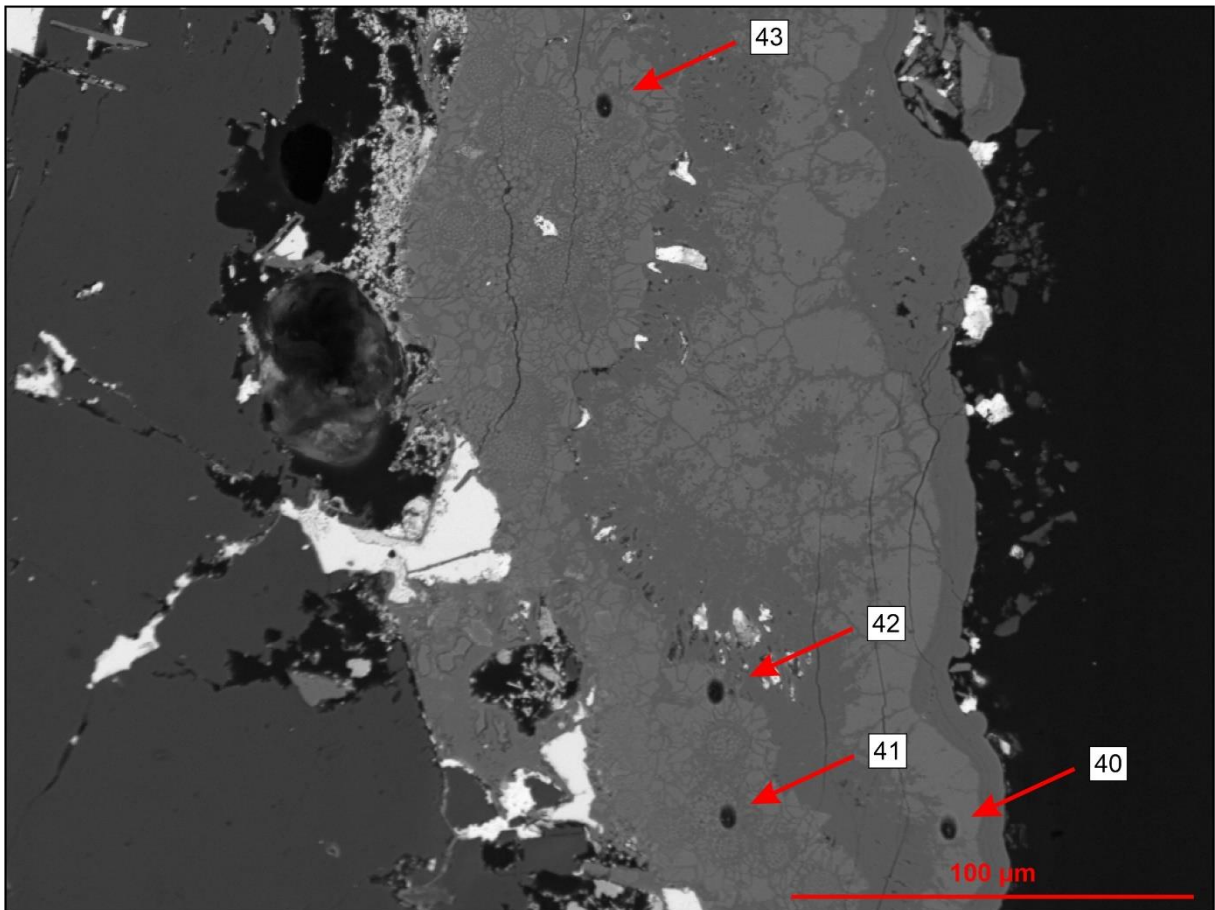

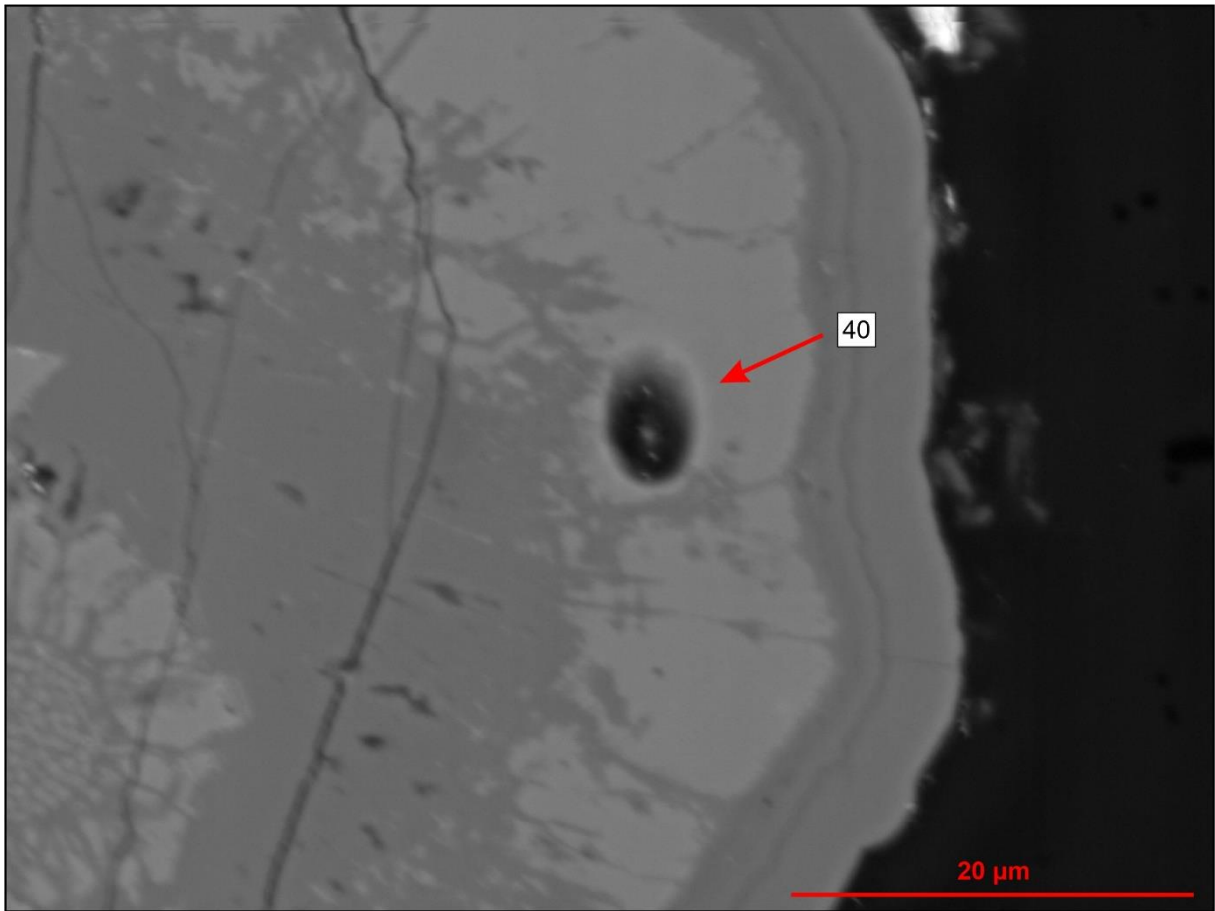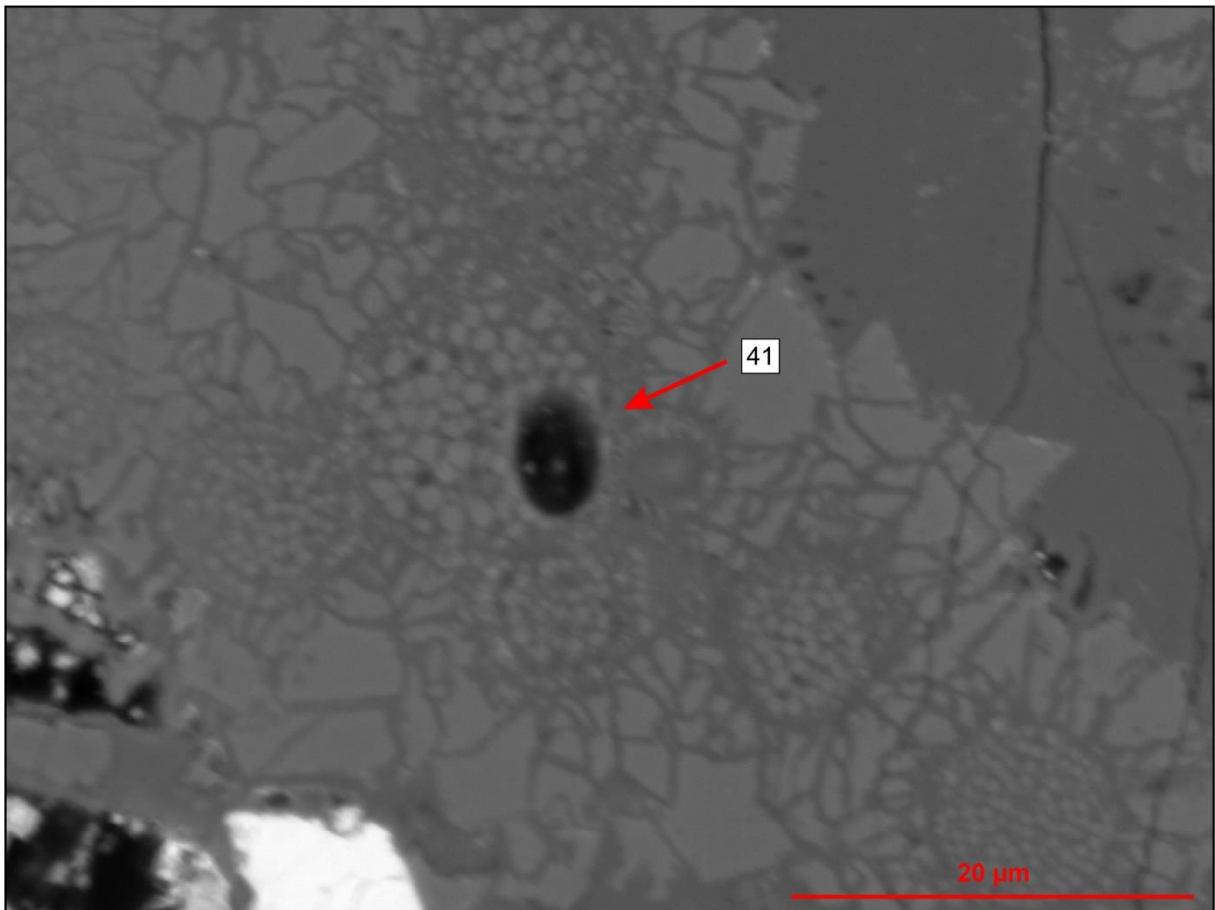

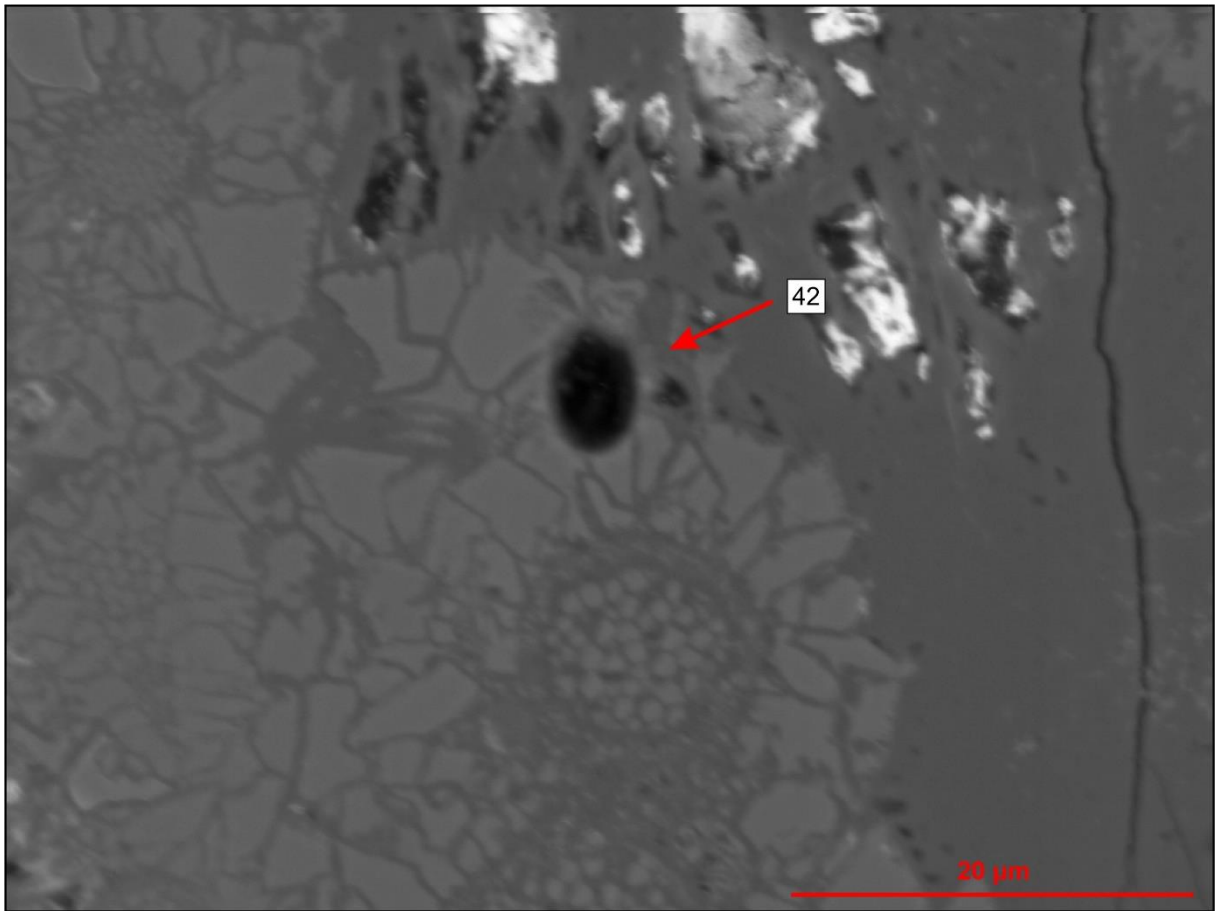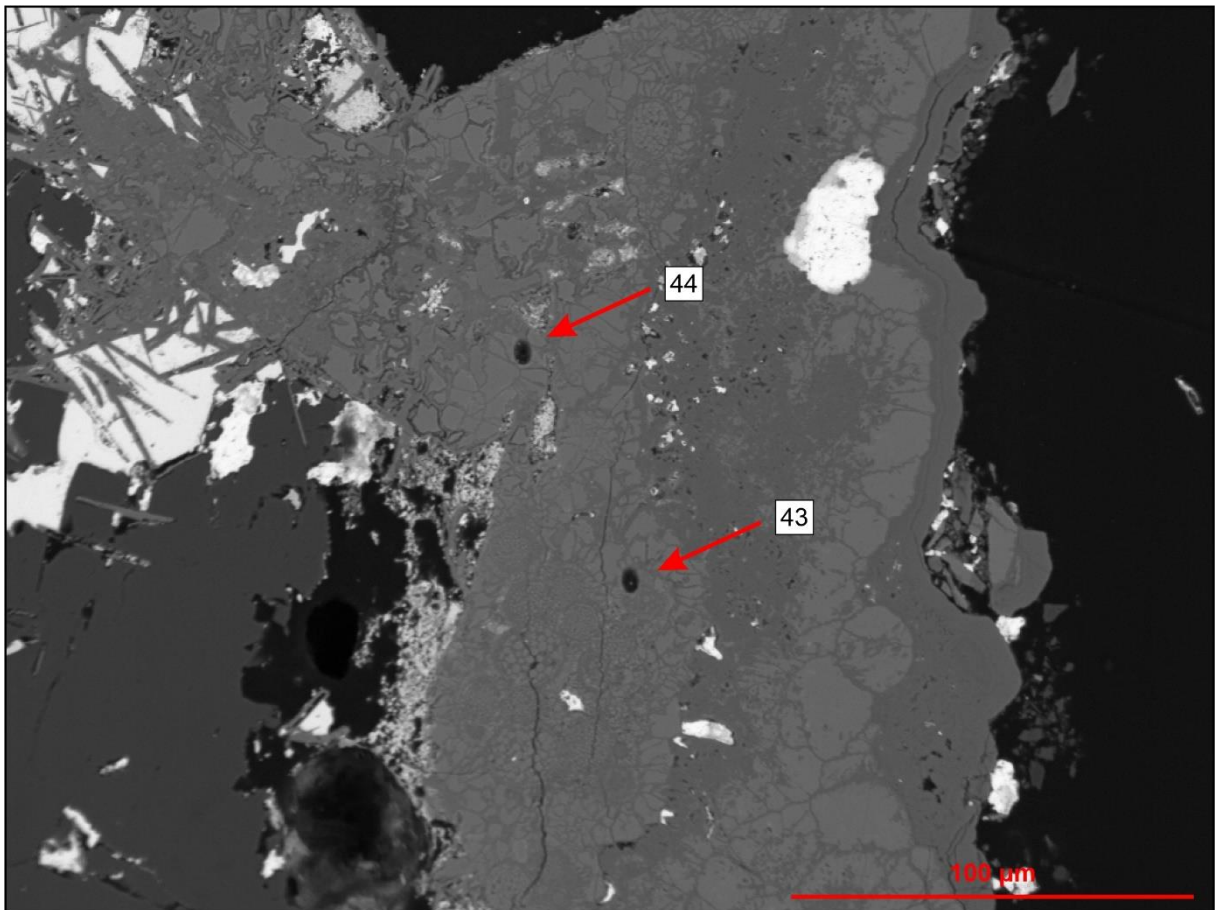

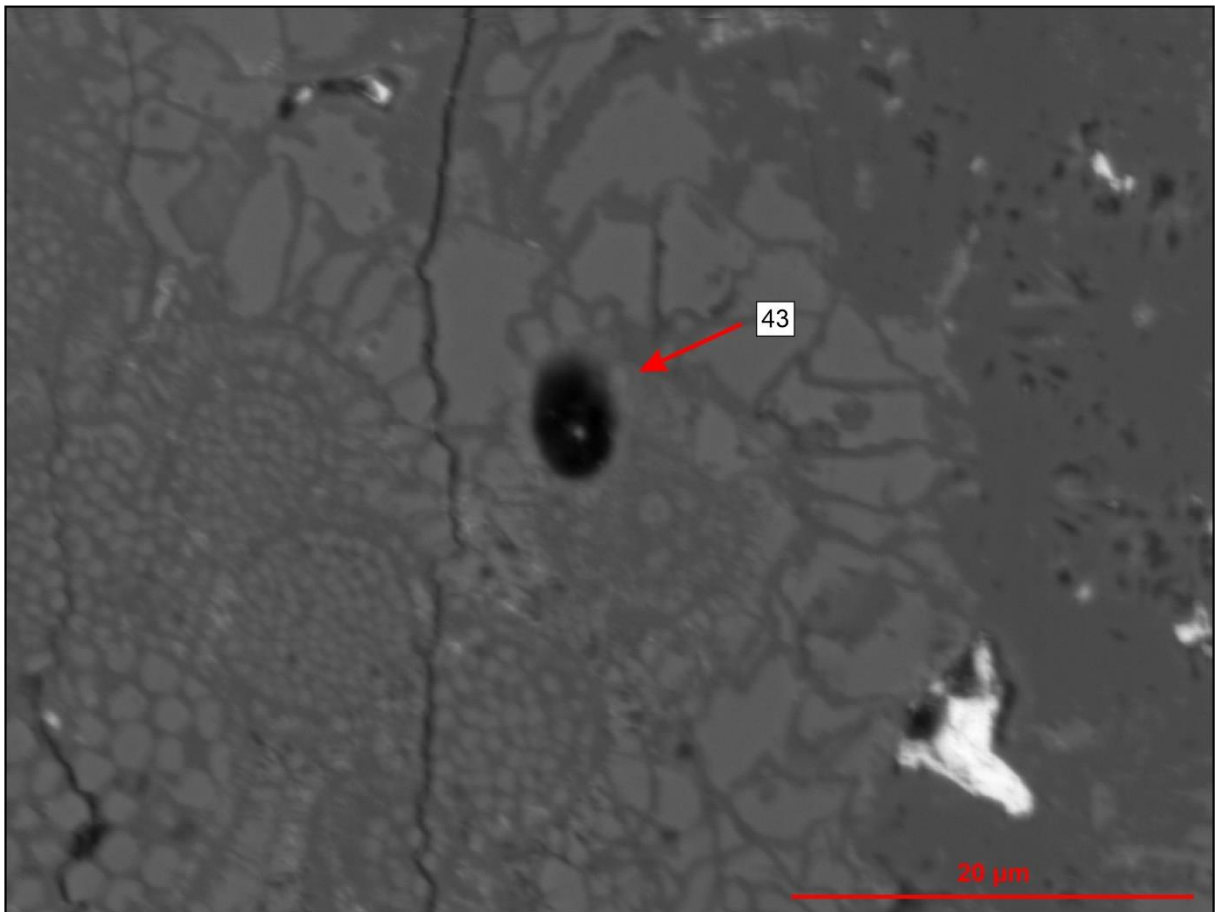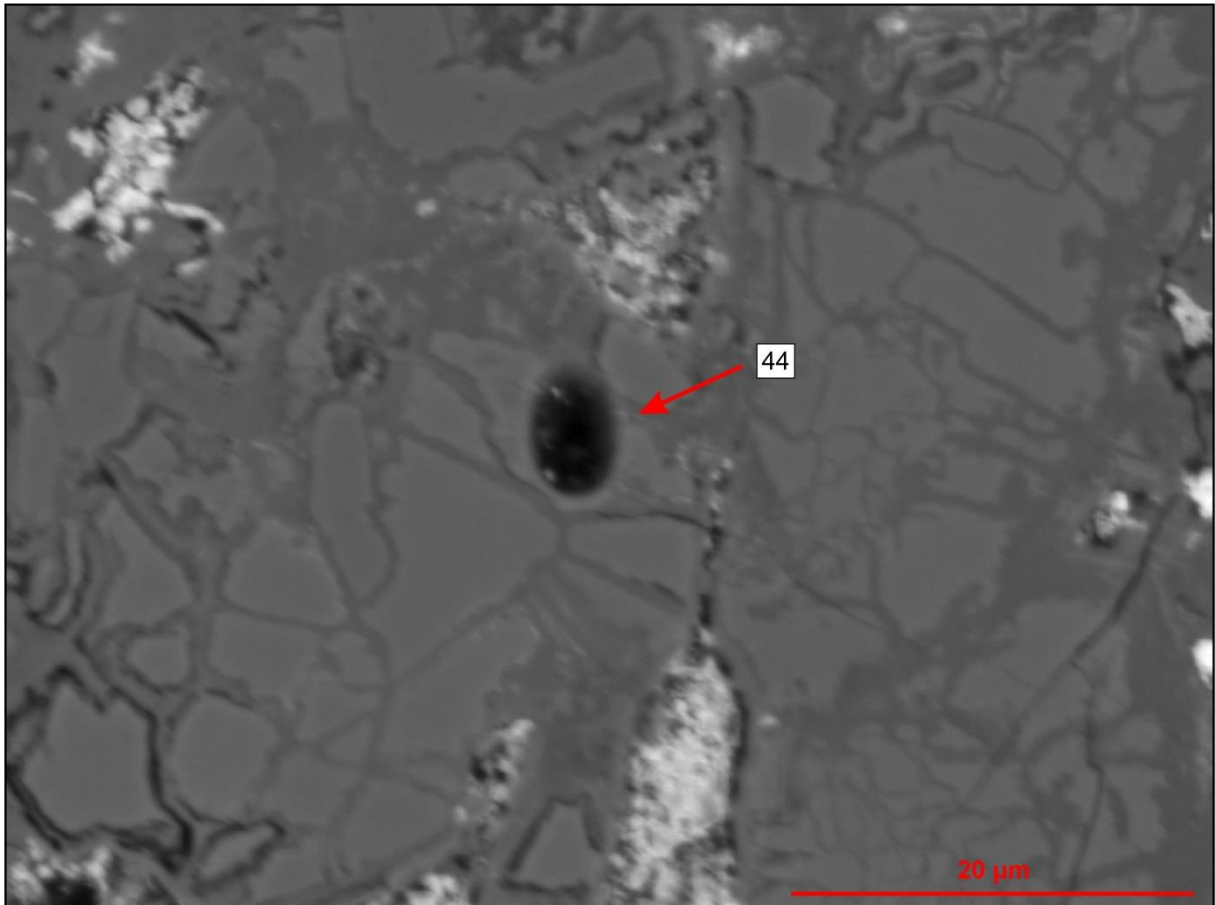

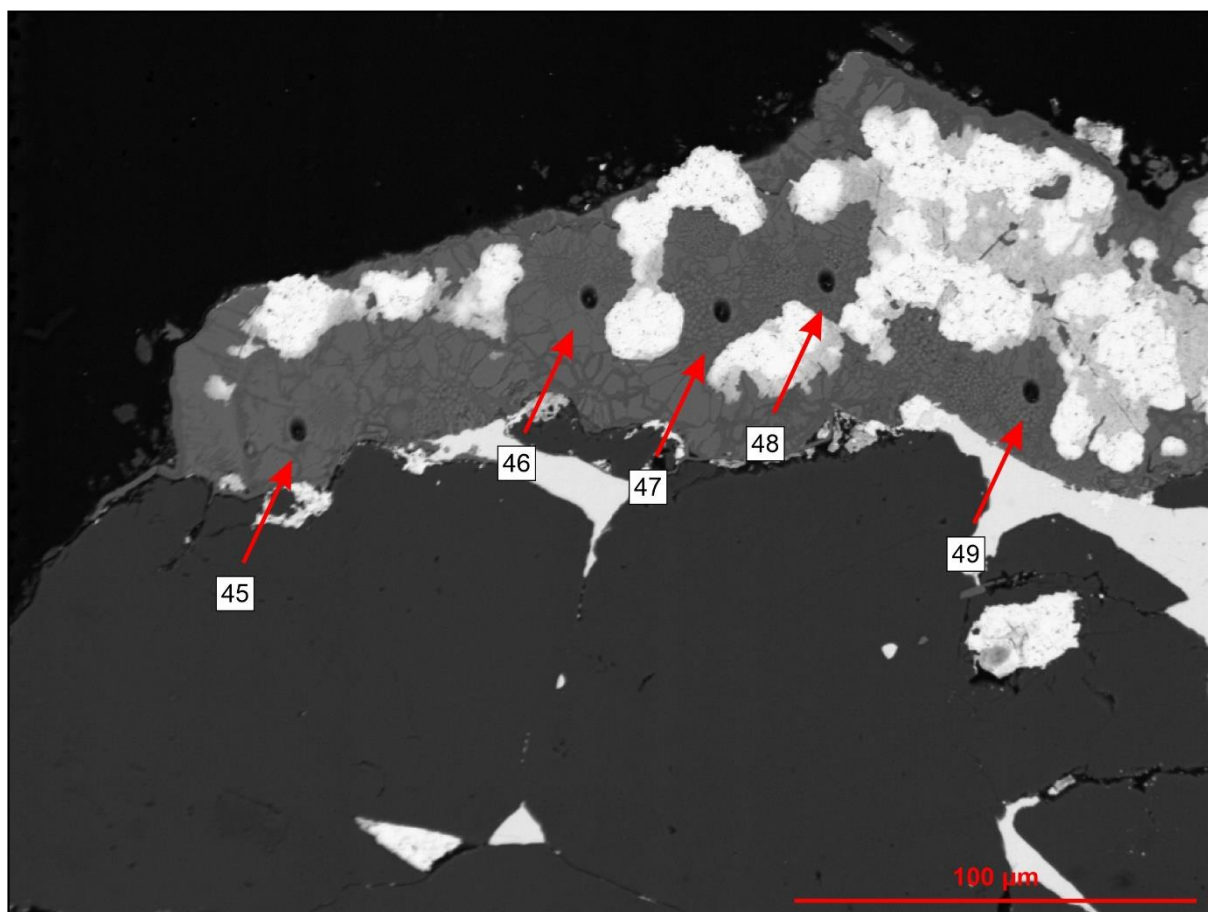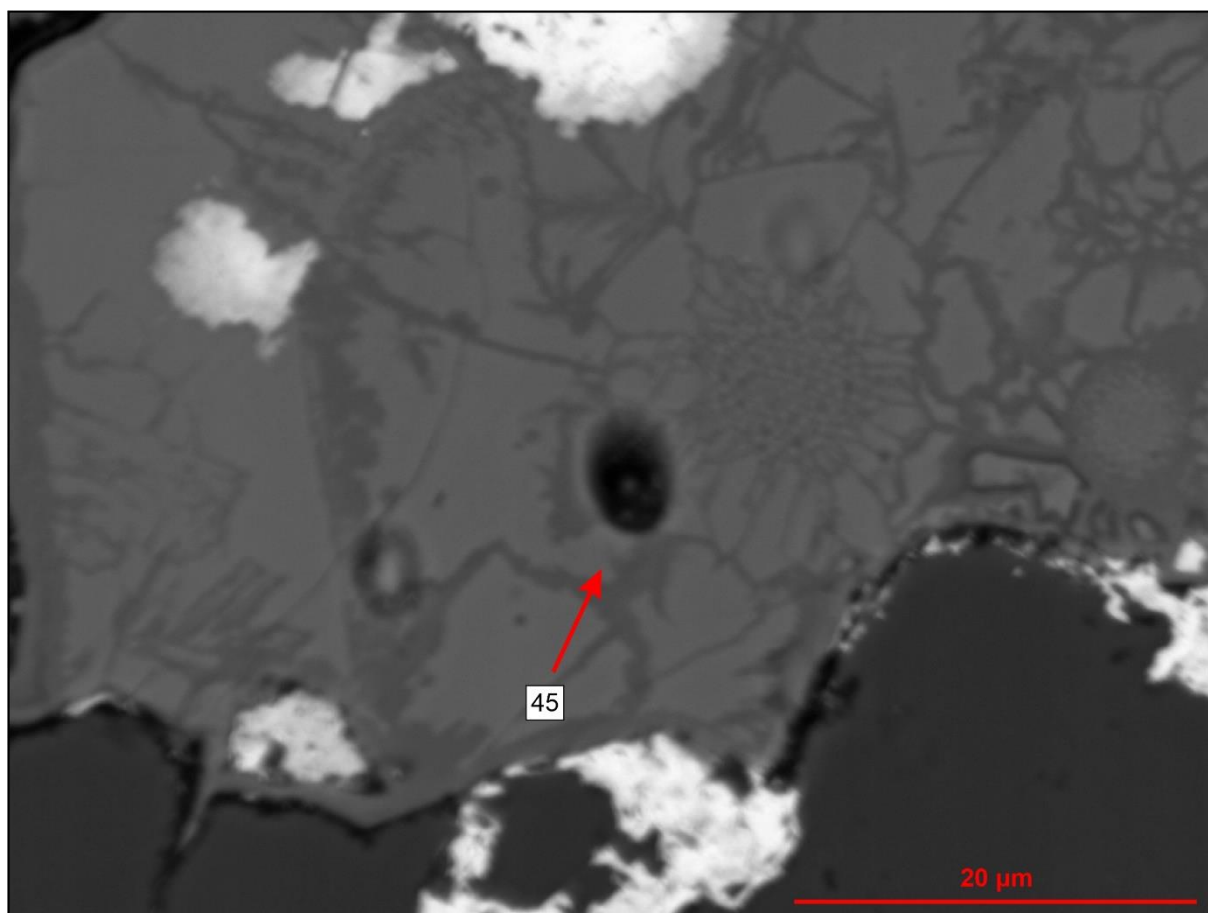

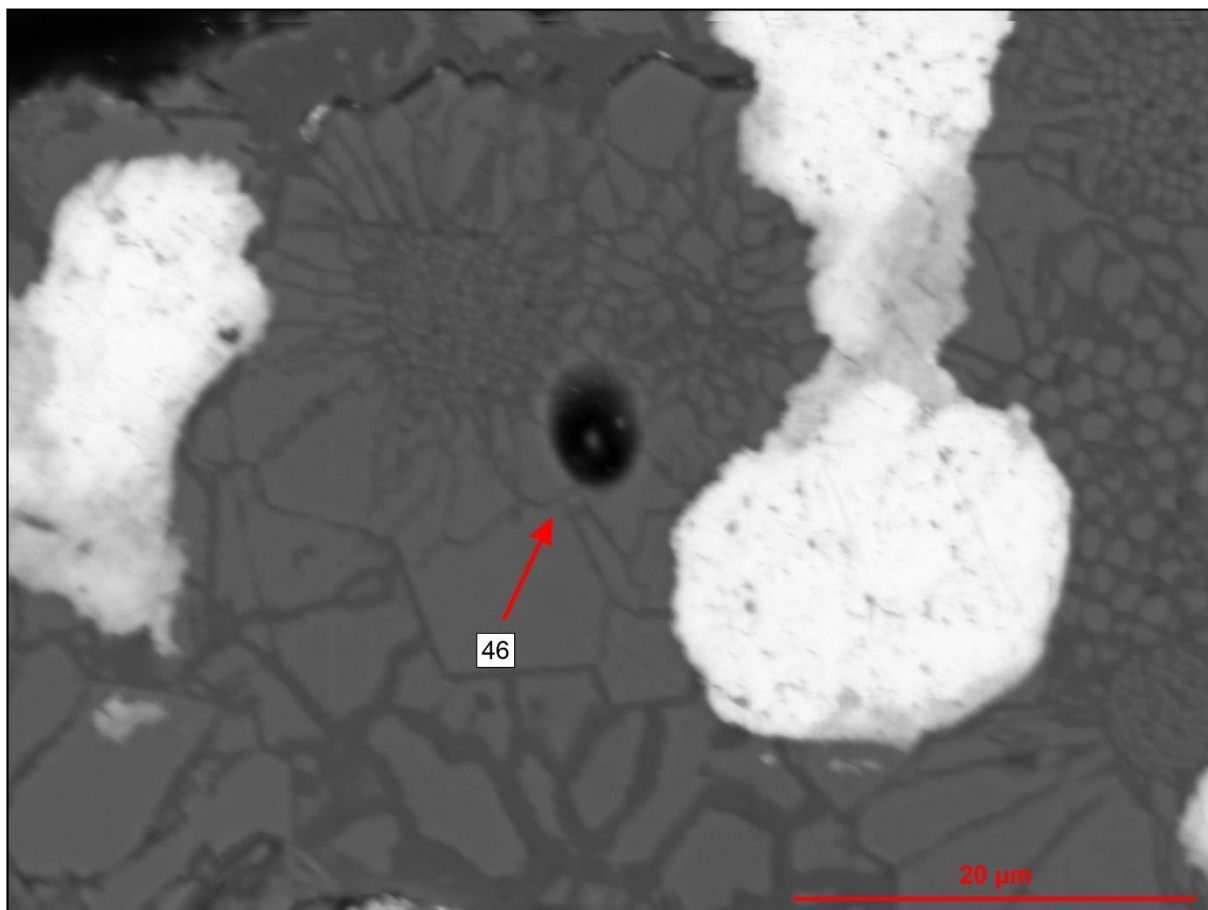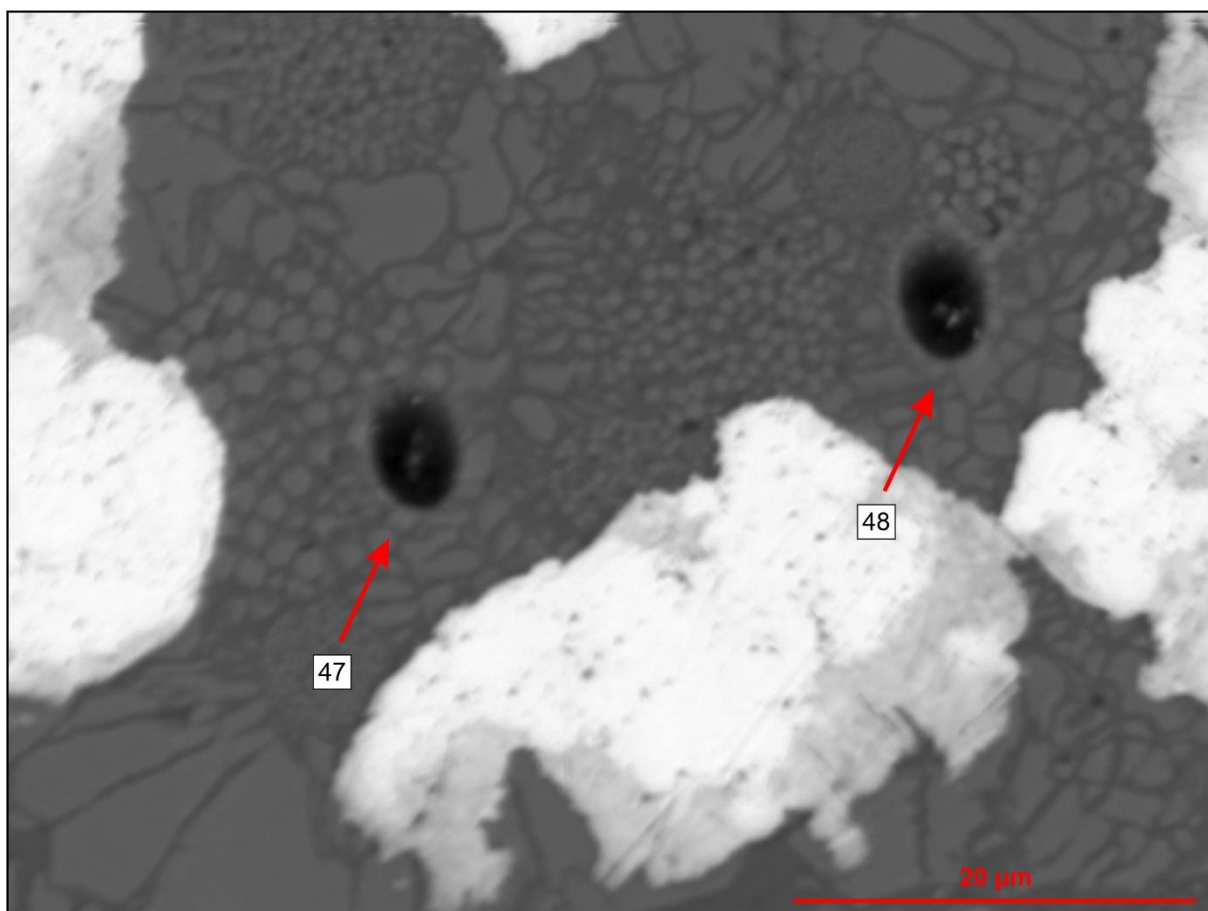

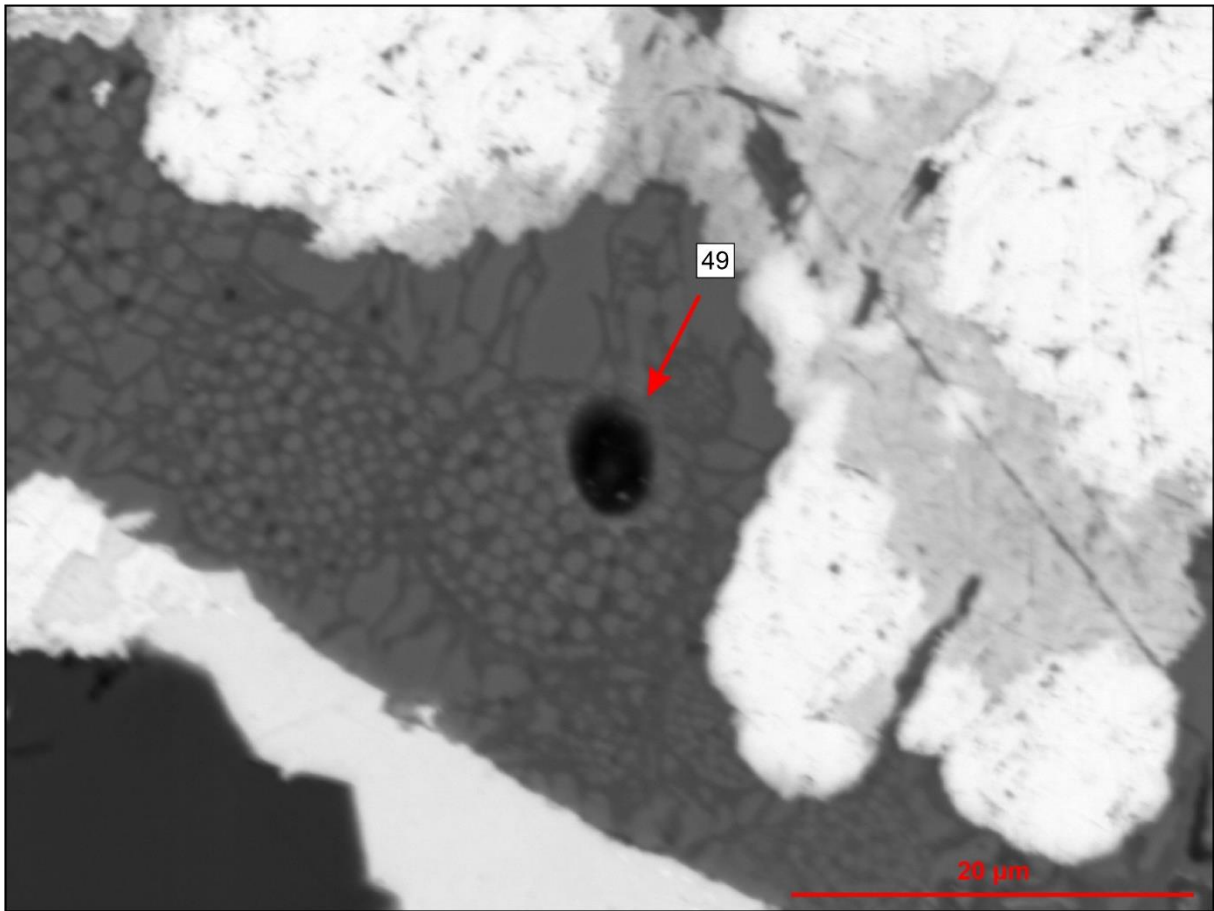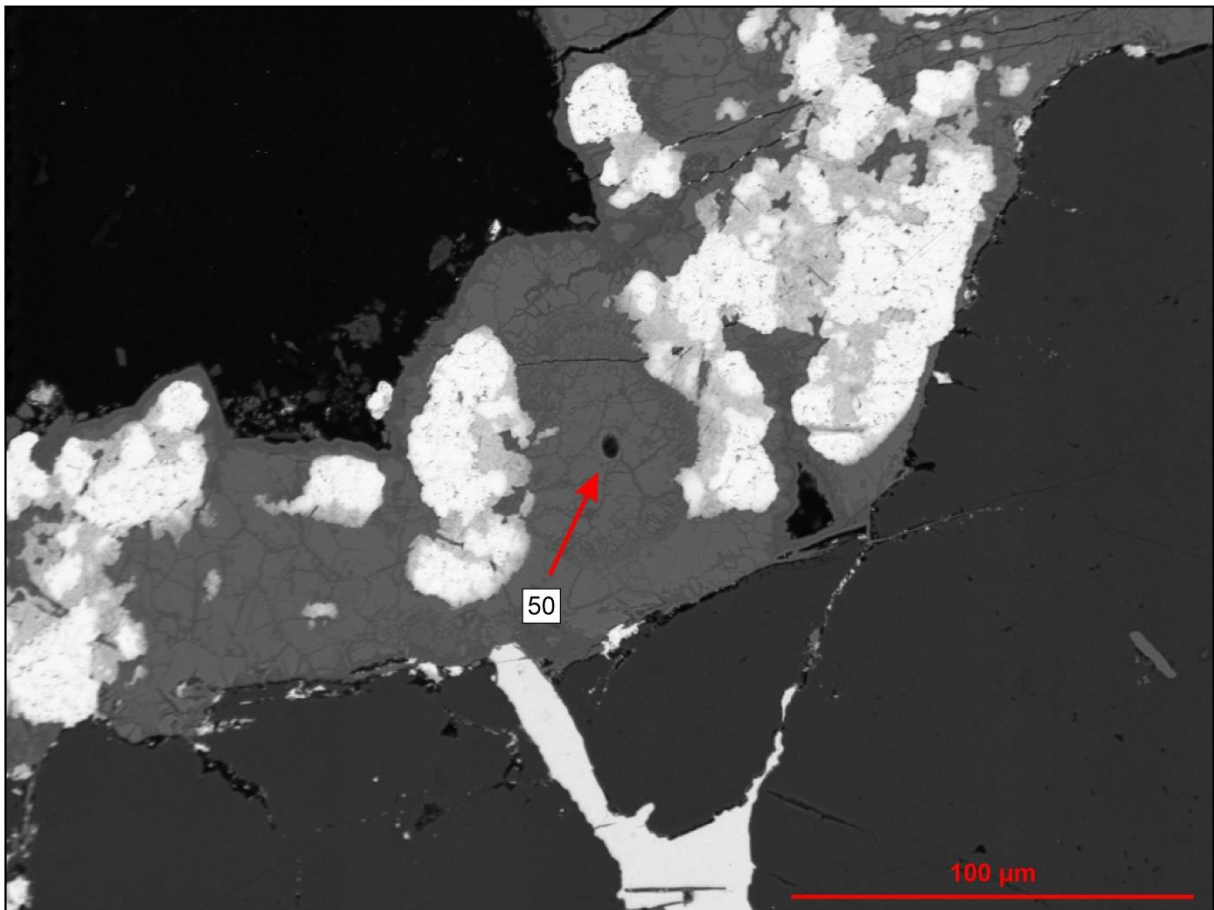

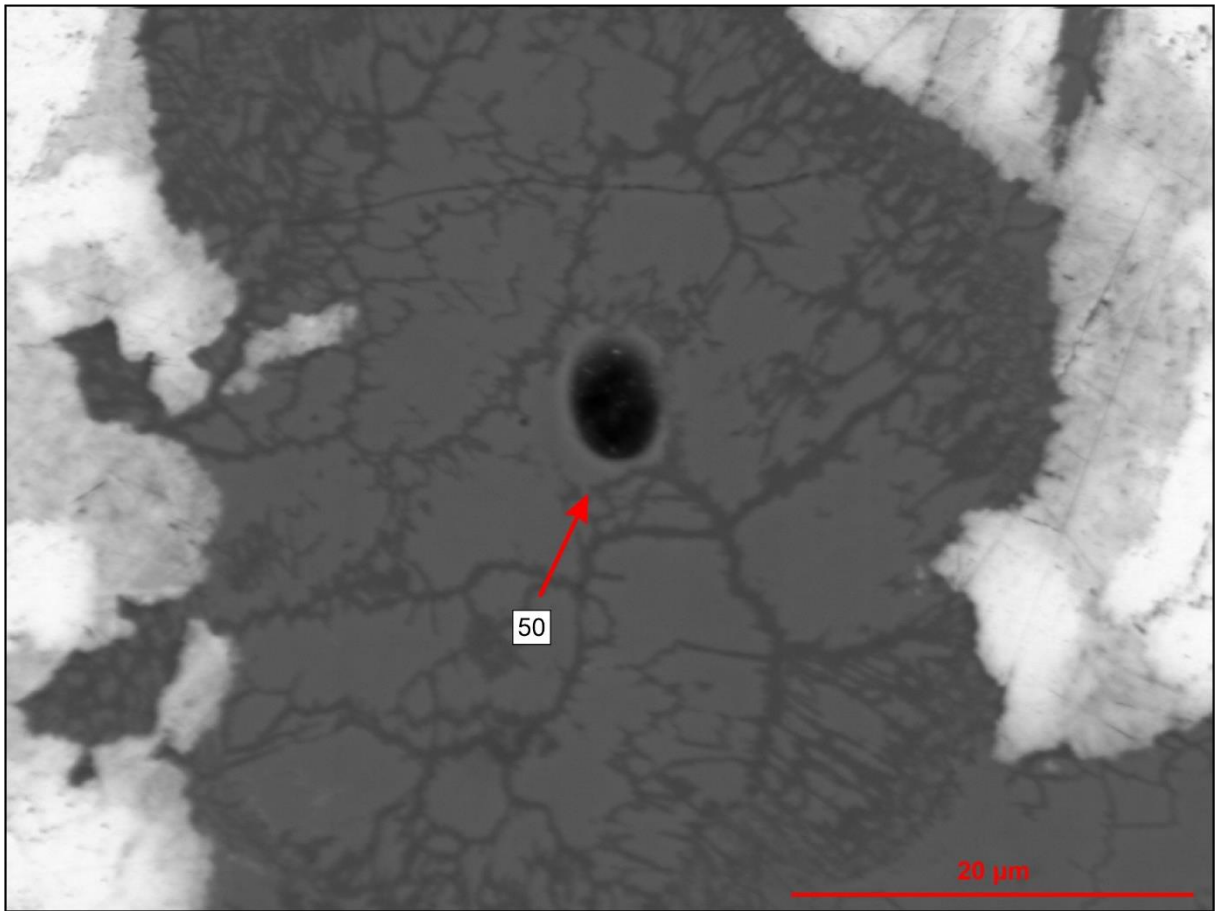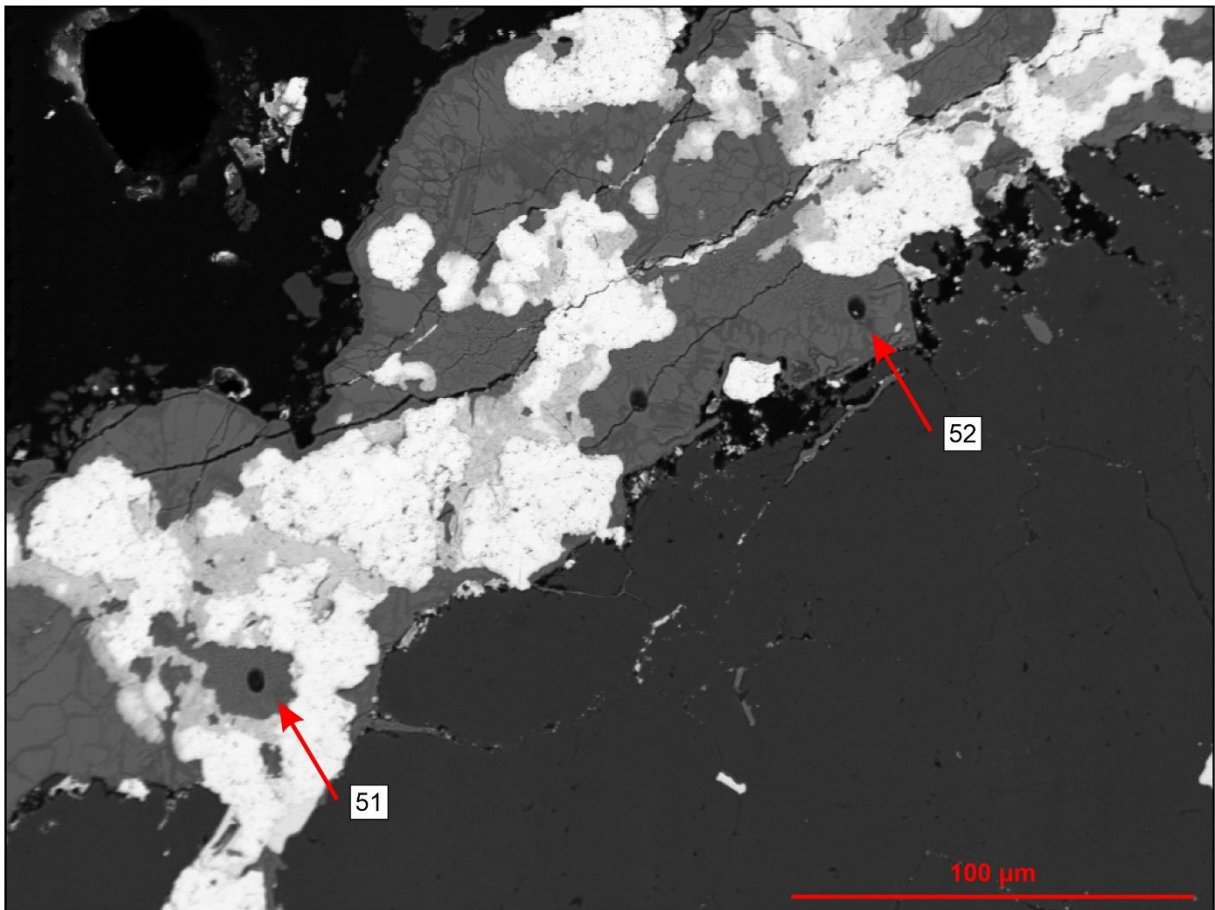

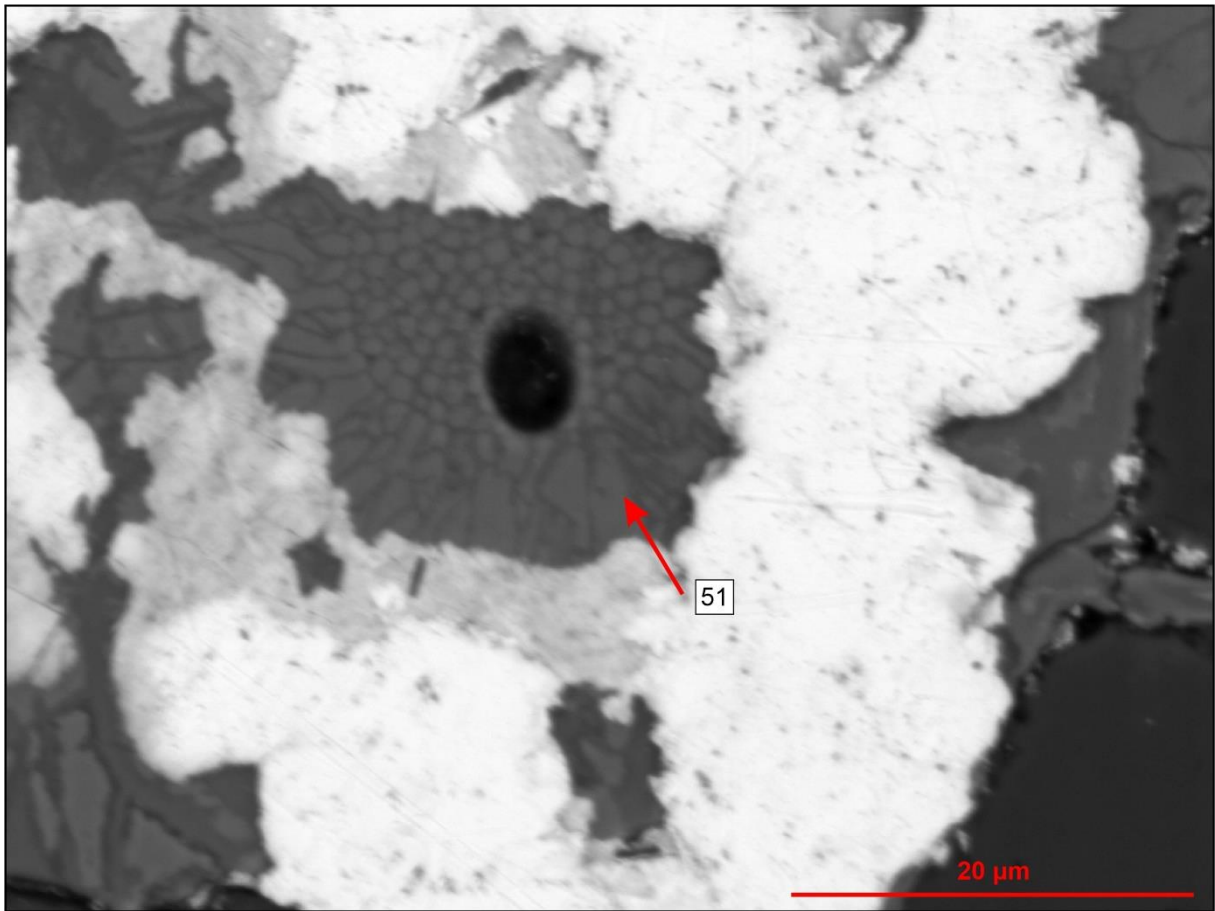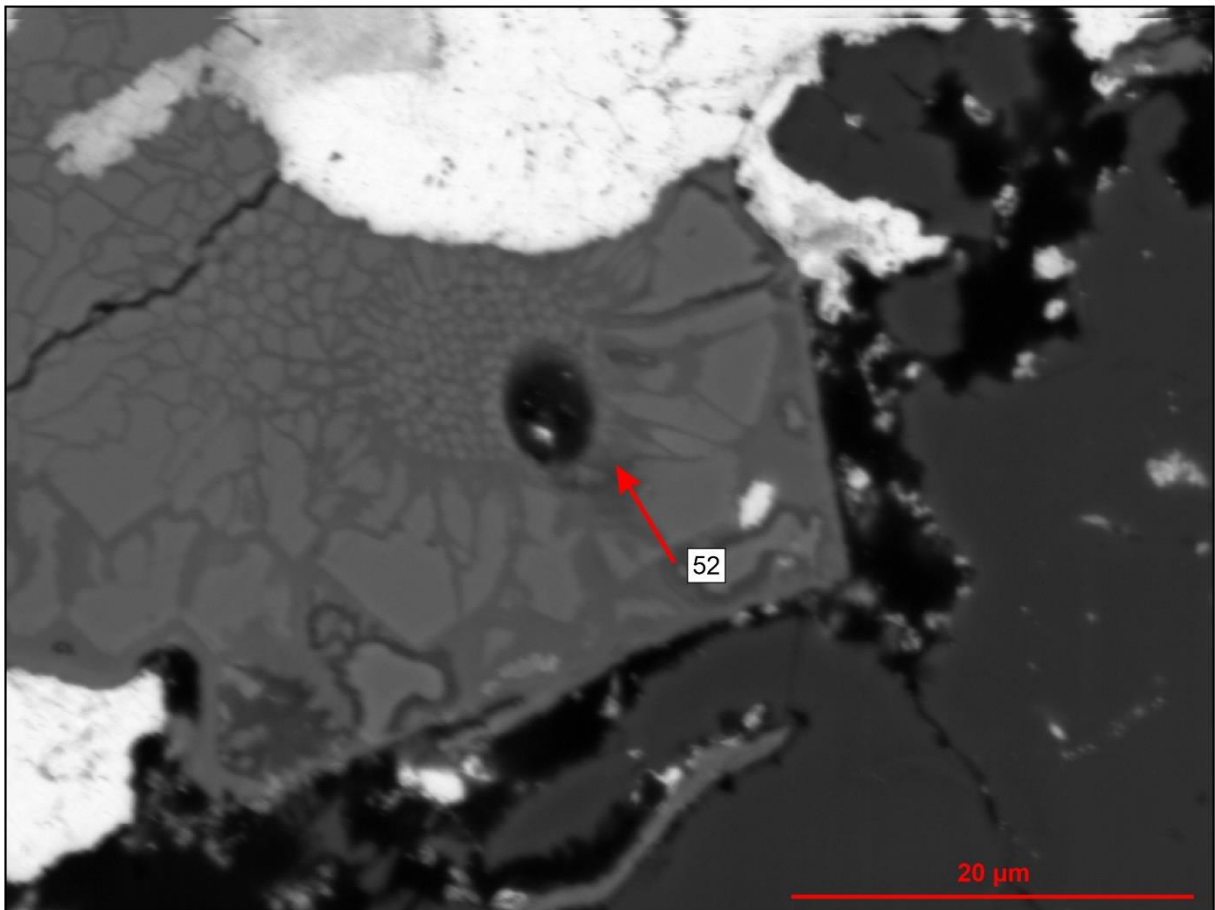

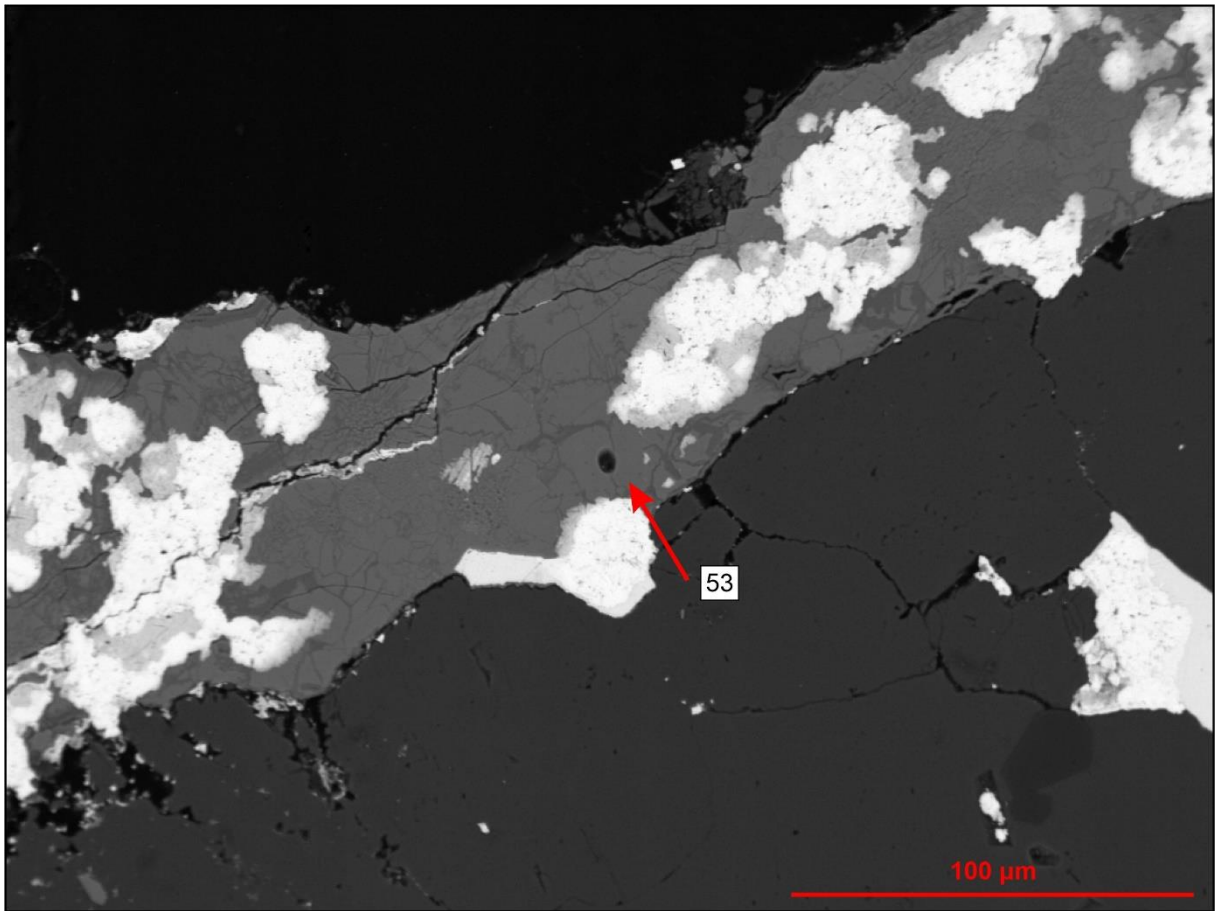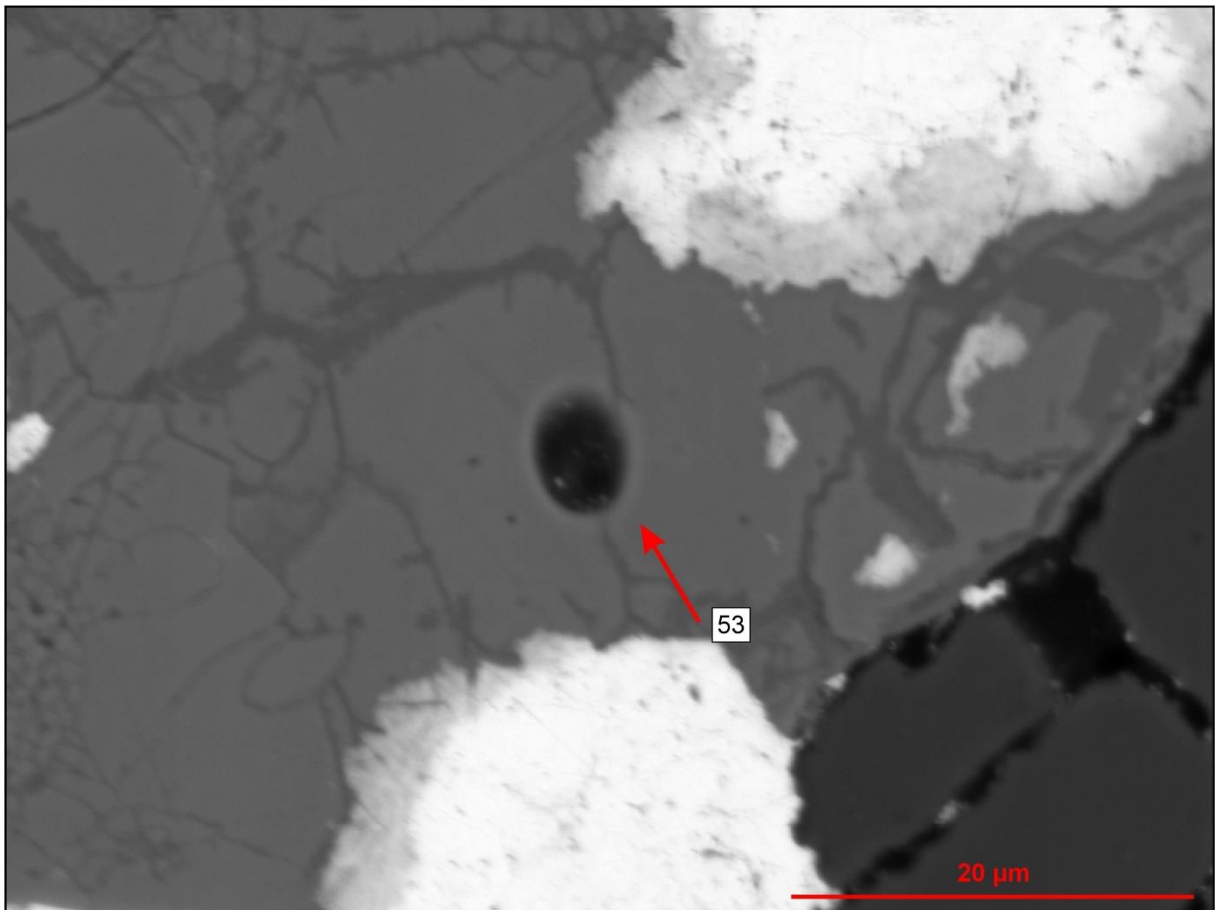

Supplement: Supplementary file 2 — Supplementary Material 2 [file 41598_2026_59857_MOESM2_ESM.pdf]

## Slide 1
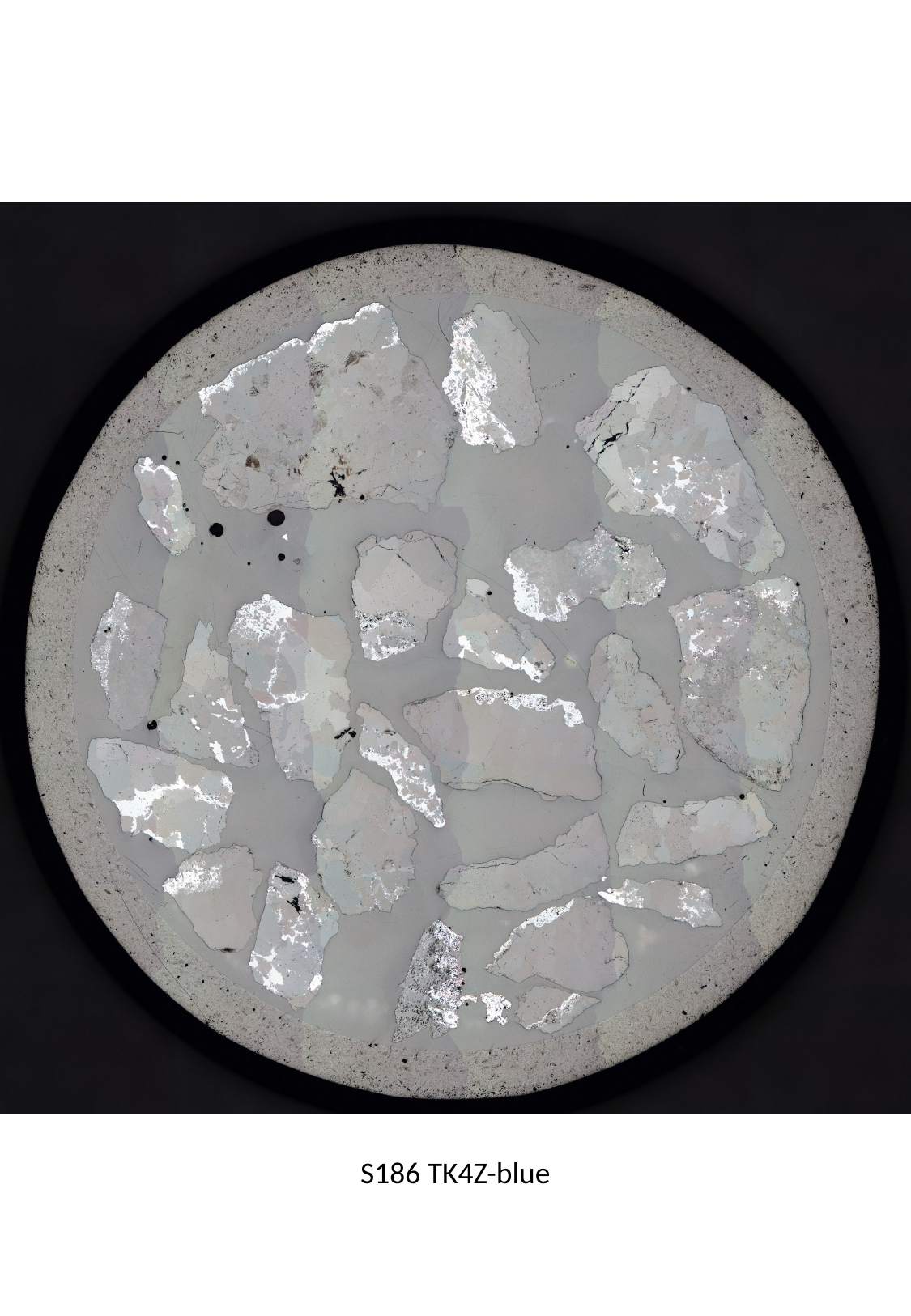

S186 TK4Z-blue

## Slide 2
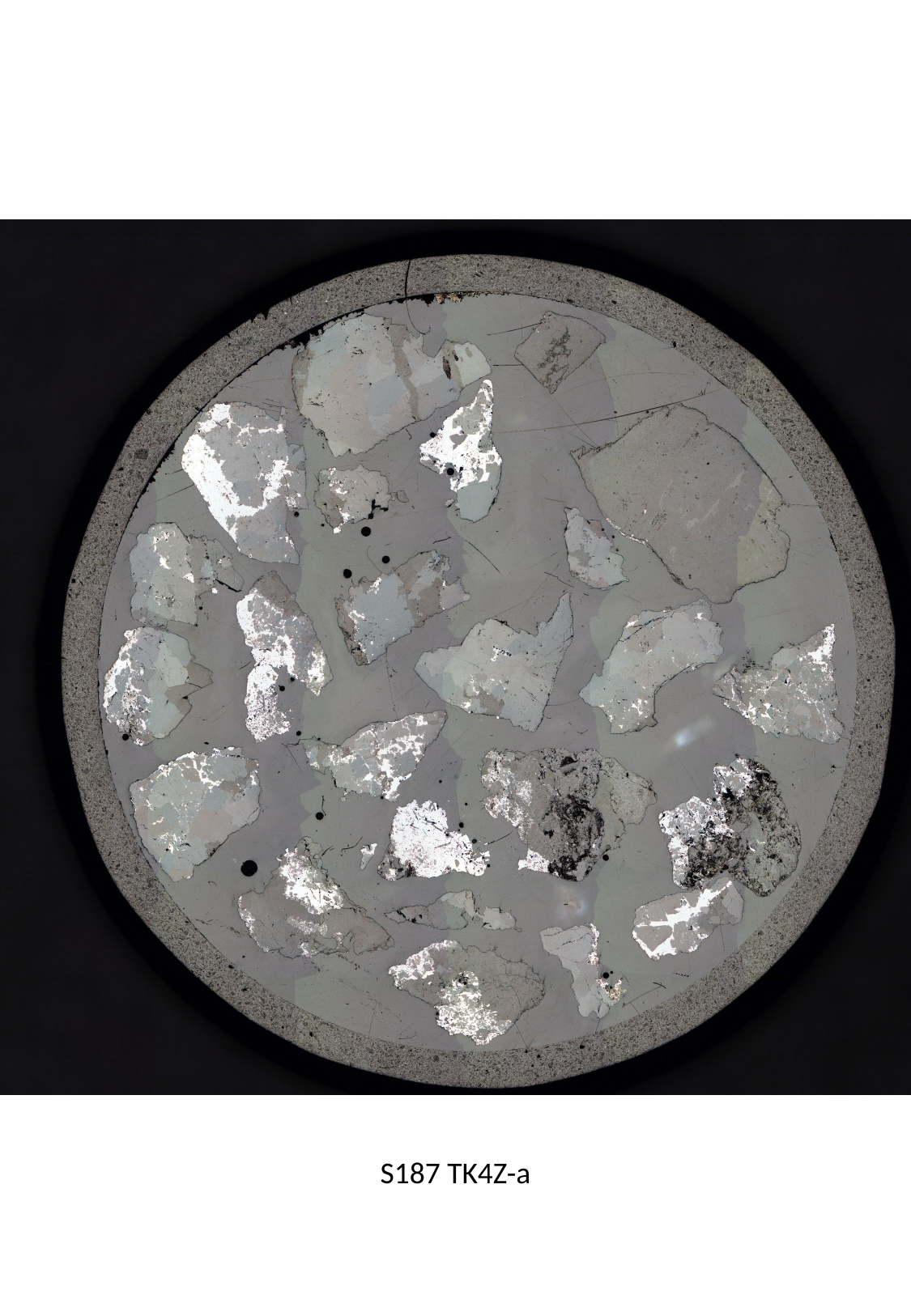

S187 TK4Z-a

## Slide 3
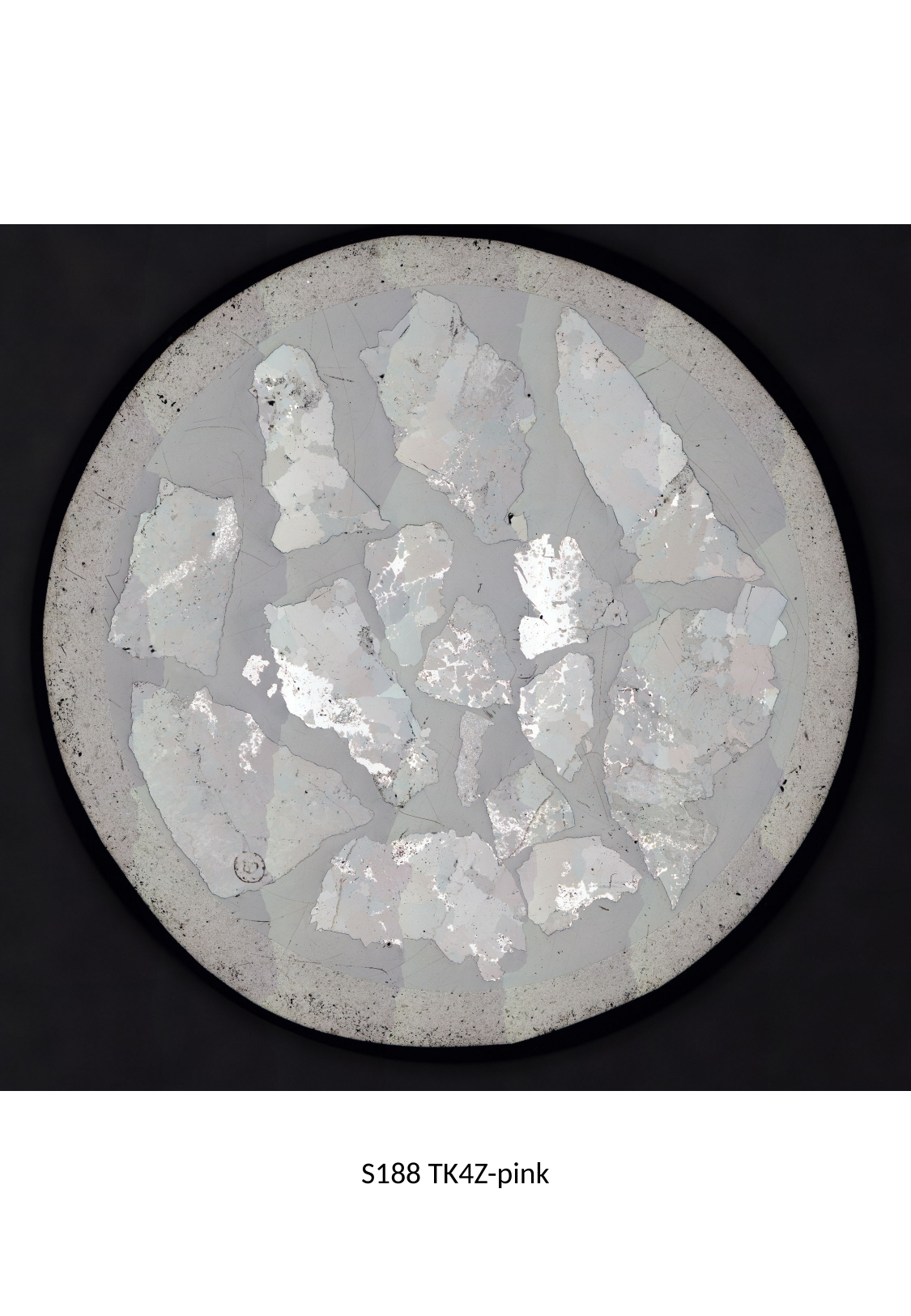

S188 TK4Z-pink

## Slide 4
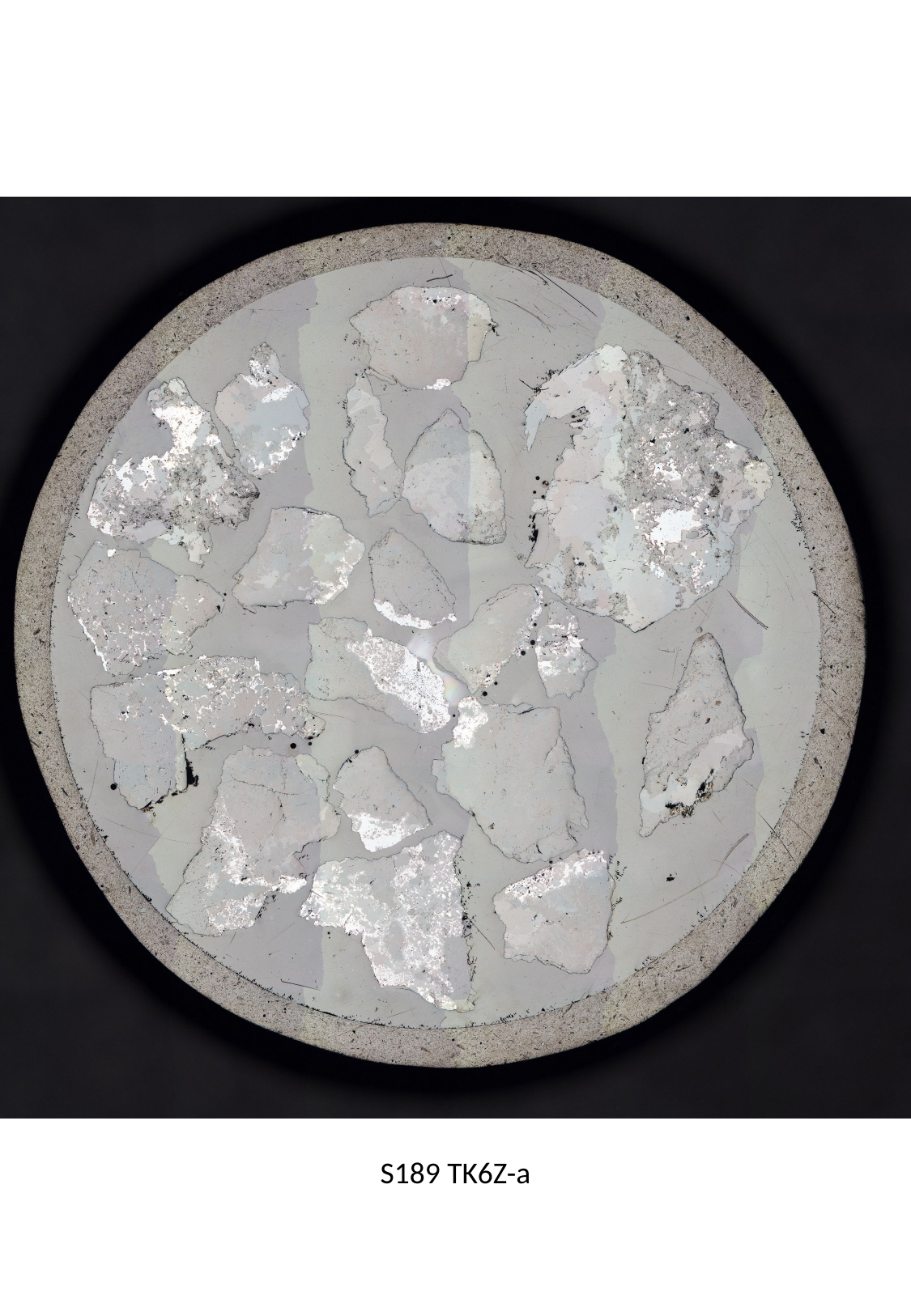

S189 TK6Z-a

Supplement: Supplementary file 3 — Supplementary Material 3 [file 41598_2026_59857_MOESM3_ESM.pptx]
